# Optimization of Microchannels and Application of Basic Activation Functions of Deep Neural Network for Accuracy Analysis of Microfluidic Parameter Data

**DOI:** 10.3390/mi13081352

**Published:** 2022-08-20

**Authors:** Feroz Ahmed, Masashi Shimizu, Jin Wang, Kenji Sakai, Toshihiko Kiwa

**Affiliations:** Graduate School of Interdisciplinary Science and Engineering in Health Systems, Department of Medical Bioengineering, Okayama University, 3-1-1, Tsushima-naka, Kita-ku, Okayama 700-8530, Japan

**Keywords:** microfluidics, fluid dynamics, 3D simulation, ReLU dense layers, Leaky ReLU, swish activation functions, deep learning model

## Abstract

The fabrication of microflow channels with high accuracy in terms of the optimization of the proposed designs, minimization of surface roughness, and flow control of microfluidic parameters is challenging when evaluating the performance of microfluidic systems. The use of conventional input devices, such as peristaltic pumps and digital pressure pumps, to evaluate the flow control of such parameters cannot confirm a wide range of data analysis with higher accuracy because of their operational drawbacks. In this study, we optimized the circular and rectangular-shaped microflow channels of a 100 μm microfluidic chip using a three-dimensional simulation tool, and analyzed concentration profiles of different regions of the microflow channels. Then, we applied a deep learning (DL) algorithm for the dense layers of the rectified linear unit (ReLU), Leaky ReLU, and Swish activation functions to train and test 1600 experimental and interpolation of data samples which obtained from the microfluidic chip. Moreover, using the same DL algorithm, we configured three models for each of these three functions by changing the internal middle layers of these models. As a result, we obtained a total of 9 average accuracy values of ReLU, Leaky ReLU, and Swish functions for a defined threshold value of $6\times 10^{-5}$ using the trial-and-error method. We applied single-to-five-fold cross-validation technique of deep neural network to avoid overfitting and reduce noises from data-set to evaluate better average accuracy of data of microfluidic parameters.

## 1. Introduction

Microfluidics technology is used to manipulate liquids within microfluidic networks of microchannels, where flow-field patterns of fluids are exhibited in the form of monophasic and multiphasic continuous flows or discrete microdroplets within the femto-to-microliter scale ($10^{-15}$–$10^{-6}$ L). Owing to the small dimensions of microfluidic systems, their unique and significant physical properties [1,2] make them versatile for a wide range of applications in material science, ecological screening, microscale physics, in vitro diagnostics using organ-on-chips [3], drug discovery [4], high throughput screening of cancer cells for cell therapy [5], food safety [6], automated blood cell detection and counting [7], blood sample testing [8], and biotechnology process control [9]. Moreover, moderate, precise, cheap, and accessible microfabrication techniques have been developed and applied in the microfluidics field over the last two decades. These techniques involve manipulating fluid mixtures with precise control to satisfy the requirements, making them applicable to fluid mechanics and interdisciplinary research. External input devices such as peristaltic pumps, digital pressure machines, and digitally modulated pressure controllers are probably the simplest conventional mechanisms for generating set values of microfluidic parameters (such as the fluid flow rate and pressure drop) [10] and analyzing the manipulation of different fluid mixtures through microflow channels of microfluidic systems. Moreover, these input devices generate pulsatile and oscillating fluid flow [11] through the microflow channels. The parameters can vary from their original generating values to instantaneous effective values of different microfluidic parameters, such as the pressure drop, velocity, volumetric flow rate, and diffusivity. Readings of microfluidic parameters, such as the volumetric flow rate and pressure drop, can be selected from random calibration using these devices as inputs to obtain a limited number of experimental measurement data [10], laser scanning image data [12] and microparticle image velocimetry data [13]. However, the output readings obtained using the trial-and-error method through the random calibration of such input devices do not confirm the full accuracy of the microfluidic system performance. For the known measured output values, the input values generated using the devices cannot reveal the corresponding effective input values of microfluidic parameters experimentally. Moreover, real-time experimental measurements using the trial-and-error method are time-consuming and associated with human-controlled errors if high precision of the analyzed data is required.

Deep learning [14,15,16] and Machine learning (ML) algorithms [17,18] improve the accuracy of microfluidic system performance because they process large amounts of data from experiments, field measurements, and large-scale numerical simulations [19] in fluid dynamics. Therefore, DL and ML facilitate modular and agile modeling frameworks for solving many problems in fluid mechanics, such as experimental data processing, reduced-order modeling, shape optimization, turbulence closure modeling, and control. Moreover, DL and ML can significantly reduce the measurement time by determining the exact input values corresponding to the measured output values of the microfluidic parameters of the designed microfluidic chip. Zheng, Jiahao et al. [20] summarized the state-of-art review of artificial intelligence (AI) in microfluidics using tables 1–3 of their article.

In this study, we proposed five-to-seven layers-based a DL algorithm to configure three different models for each of three basic activation functions of deep neural networks (DNNs), called dense layers of rectified linear unit (ReLU) [21,22], Swish [23,24] and, Leaky ReLU [25,26] activation functions. We configured the DL model as simple as possible. Because we employed to train up to 1600 experimental and interpolated data samples from capillary-design-based microflow channels of a microfluidic chip. For such small data-set, we did not employ any other complex DL algorithm. The advantages of using the above-mentioned three basic functions of DNN over others AI-based microfluidic technology are to reduce the computational complexity, cost and operational congestion. Before applying the DL model, the designed microfluidic chip was optimized using the simulation scale of a three-dimensional (3D) simulation tool. We analyzed the concentration profiles of different regions of the microflow channels to clarify the flow dynamics of the mixture of two buffer solutions with pH values of 4 and 10. Such an analysis is significant for optimizing the microflow channels and determining the data measurement range, specifically in terms of velocity and volumetric fluid flow rate. Therefore, we selected each set value of the volumetric fluid flow rates as inputs, ranging from 0.001 to 4.2 mL/min. We measured the corresponding outputs of the volumetric fluid flow rates at the outlet well of our designed a 100 μm height of the rectangular and circular channels-based microfluidic chip, and made the interpolation of data samples. Initially, in case of ReLU function, we applied a single train-validation-test split technique of a simple DL algorithm to obtain training and validation losses with respect to 20 epochs for the defined threshold value of $10^{-5}$. However, they caused overfitting during the data-learning process. Therefore, we applied another DL algorithm of dense layers of ReLU, Swish and Leaky ReLU to test the data by configuring three different models for each of them. However, we kept each model same except the change in internal middle layers of the DL algorithm. As a result, total 9 average accuracy values obtained for the defined threshold value of approximately $6\times 10^{-5}$ using the trial-and-error method. It evaluated percentages of average accuracy values for all basic functions using the k-fold cross-validation technique [27,28,29]. This k-fold cross-validation technique reduced the noise and avoided overfitting from data samples. In this study, we used a single-to-five-fold cross-validation technique. Here, sample data trained with epochs of 20 and batch size of 50.

## 2. Materials and Methods

### 2.1. Simulation for Microfluidic Channel Optimization

**Figure 1:**
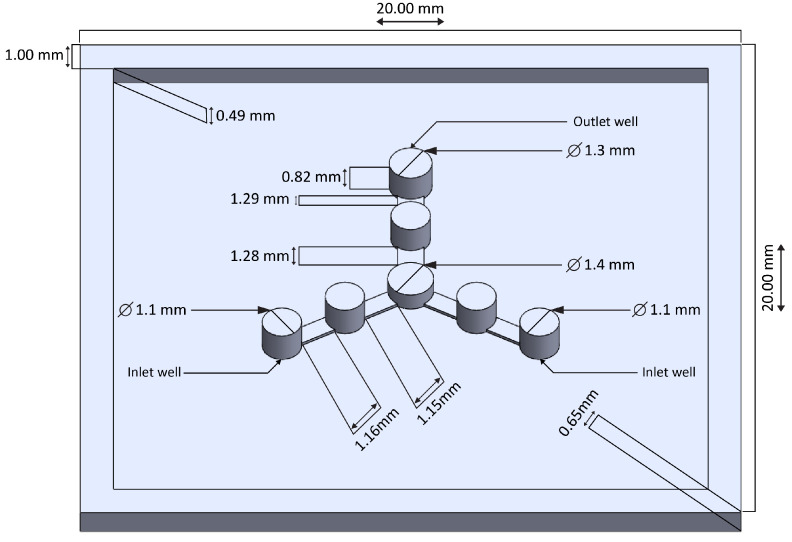
Using a 3D tool, geometric details of the designed 3D structure drafted to prepare a replica mold of PDMS-made microfluidic chip.

A 3D simulation tool was used to optimize the dimensions of the microflow channels of the microfluidic chip. We followed the similar steps of the simulation algorithm of sub-section A of the article of Ahmed et al. [10] where, the geometric details of the designed structure (Figure 1) were used as input data to simulate the multiphase flow of the two pH buffer fluids. From Table II of the article of Ahmed et al. [10], we took viscosities and densities of individual and mixed pH 4 and 10 buffer solutions. We activated all governing equations associated with the mass, momentum, and energy conservation of fluid dynamics, followed by the sub-sections B and C (governing equations and numerical settings) and Appendix C of the article of Ahmed et al. [10]. We considered surface roughness (0.001 µm) value inside the microfluidic channels before running the simulator. Then, using 3D simulation tool, we made cross-sections of different regions of flow patterns of two pH fluids to reveal concentration profiles of microflow channels to clarify the behavior of flow dynamics. Moreover, from the simulation, we observed the distribution of charge particles within the inner regions of microflow channels of the microfluidic chip.

### 2.2. Fabrication of Microfluidic Chip and Experiments for Data Measurements

**Figure 2:**
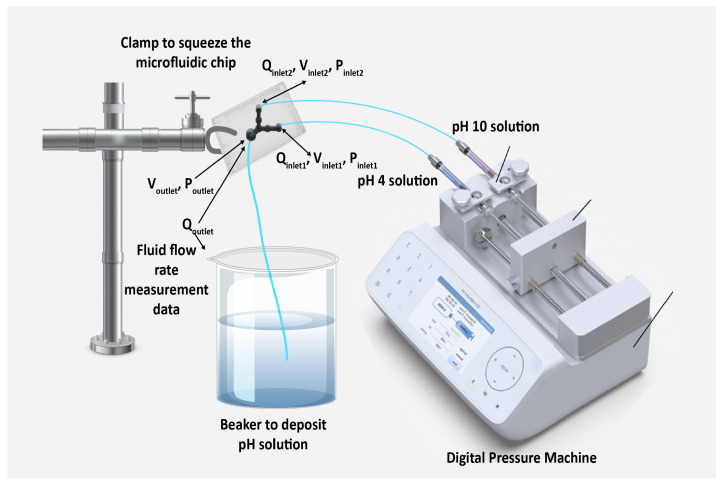
Using digital pressure machine, experimental measurement set up of a PDMS-made microfluidic chip to determine data of microfluidic parameters.

In this study, polydimethylsiloxane (PDMS)-made circular and rectangular microflow channels with a height of 100 μm rectangular channels-based a microfluidic chip of two inlet wells and an outlet well were designed. The channels drafted using a 3D tool and printed using a 3D printer (Agilista-3100; serial no.: 96M14458). Next, the 3D-printed structure was poured with a Sylgard® 184 silicone elastomer base agent and a Sylgard®184 silicone elastomer curing agent (manufactured by Dow Corning (Midland, MD, USA)) at a ratio of 10:1 to prepare the replica mold of the PDMS-made microfluidic chip for performing the laboratory experiments. The 3D structure drafted using the 3D tool is depicted in Figure 1. A digital pressure machine (Model fusion 710, Chemyx Inc., Stafford, TX, USA) employed to generate two buffer solutions with pH values of 4 and 10 to measure the volumetric fluid flow rates at the outlet well of the microfluidic chip. For the micro-fabrication process of the microfluidic chip, we employed similar procedure of sub-section B of method section [10] and experiments section [12]. Using Equation (A1) of appendix A of the article of Ahmed et al. [10] and different dimensions of wells of Figure 1, the microfluidic resistances of two inlet wells and a outlet well calculated. From the set values of fluid flow rates, $Q_{inlet1}$ and $Q_{inlet2}$ (mL/min) of the digital pressure machine, we first calculated pressure drop across two inlet wells, $P_{inlet1}$ and $P_{inlet2}$ using Equation (1) of the article of Ahmed et al. [10]. In addition, average velocities at two inlet wells, $V_{inlet1}$ and $V_{inlet2}$ calculated from Equation (A4) of appendix A of the article of Ahmed et al. [10] and diameters of two inlet wells of Figure 1. During the measurement, the mixed two pH solutions were flushed-out automatically into a beaker through a tube from the outlet well for a certain estimated total measurement time of 32 min. Subsequently, for each output reading, we calculated the volume of the flushed-out pH solutions from the total occupied volume of mixed solutions within the beaker in milliliter. As a result, we obtained volumetric fluid flow rates, $Q_{outlet}$ (mL/min) at the only outlet well from the experimental measurement data (Figure 2). Then, from Equation (A4) of appendix A of the article of Ahmed et al. [10], we calculated average velocities, $V_{outlet}$ and pressure drops across the only outlet well, $P_{outlet}$ using Equation (1) of the article of Ahmed et al. [10]. At the final step, we made the interpolation of data samples to obtain 1600 data of corresponding microfluidic parameters. The calculated values of microfluidic resistances and other parameters are available in the excel file with the processed data used for DNN analysis (see Supplementary Materials).

### 2.3. Dense Layers of ReLU, Swish, and Leaky ReLU of DL Model

**Figure 3:**
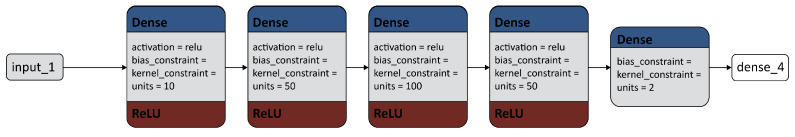
The algorithm of the internal architecture of the dense layers of the ReLU basic activation function of DL model.

**Figure 4:**
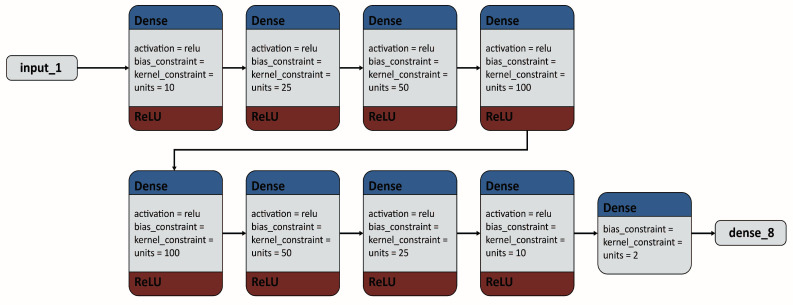
The algorithm of the internal architecture of the dense layers of the ReLU, leaky ReLU, and Swish basic functions applied to analyze the microfluidic parameter data.

Dense layers of ReLU are widely used for training processed data and testing data samples with the predicted DL model. As an activation function similar to other functions, the ReLU can determine the output of the designed model, computational efficiency, and accuracy. In contrast, Swish is an alternative and newly developed scalar activation function, which adopts a scalar as the input and outputs the scalar because scalar activation functions can be utilized instead of the ReLU basic function without changing the main network architecture. The Leaky ReLU is another activation function, an improved version of ReLU. Leaky ReLU exhibits all ReLU properties except the dying problem of the ReLU function. Generally, the training data-set for the DL approach contains tens of thousands of sample data. A larger data-set made the DL model grow deeper, and thus, the prediction accuracy increased. At the Preliminary step, a DL model of a single train-validation-test split technique applied to obtain training and validation losses with respect to 20 epochs for the defined threshold value of $10^{-5}$ in case of ReLU function. The designed DL algorithm’s internal architecture of dense layers of the ReLU function is depicted in Figure 3. However, our running DL model on small data-set could cause overfitting [30,31,32]. We had a sample size of only 1600 data; therefore, we maintained the model as simple as possible. In the next step, we attempted a new DL model, which contained only nine layers of densely connected deep neural networks. The ReLU, Leaky ReLU, and Swish functions used as basic and simple activation functions in this model. Figure 4 shows the newly-designed algorithm of the internal architecture of the dense layers of the ReLU, Leaky ReLU, and Swish basic functions to analyze the microfluidic parameter data. Keras was used for the implementation. Among the data, 90% was used for training, and 10% was used for testing. The Adam optimizer was used, and each loss function was calculated using the mean squared error.

Based on the continuity equation and Hagen’s law of fluid dynamics, we used the same set values of the volumetric fluid flow rates at each of the two inlet wells and volumetric fluid flow rate at the outlet well as input parameters. In addition, we adopted the velocities at the two inlet wells and an outlet well and the pressure drop at each of the two inlets and one outlet as output parameters. That is, we considered two input parameters and five output parameters for the DNN. Our aim is to predict the possible inputs for the desired output parameters. Therefore, real-time input was selected as the output ($V_{outlet}$, $V_{inlet}$, $P_{inlet1}$, $P_{inlet2}$ and $P_{outlet}$), and real-time output was adopted as the input ($Q_{inlet}$ and $Q_{outlet}$) to set up the DNN. The steps to implement the microfluidic data-set analysis from the perspective of DL analysis are as follows.

#### 2.3.1. Prediction of Possible Inputs

As previously mentioned, the objective is to assume possible inputs for the desired output parameters. For this purpose, the real-time input was adopted as the output and the real-time output as the input for the designed DNN algorithm. In the computer coding, $Q_{inlet}$ and $Q_{outlet}$ denoted as C1 and Cout respectively [33].

#### 2.3.2. Establishment of Deep Neural Network Model

Each of DL models (Figure 3 and Figure 4) was generated using Keras and TensorFlow [34,35]. We split the dataset into a training set (90%) and a test set (10%) of the input and output parameters using the train_test_split function, called from the sklearn library [36]. This indicated that our data were ready for setting up a DL model [33]. It is mentioned already that real-time input data were the output, and real-time output data were used as the input for the DL model. Therefore, to generate this model, we used five input data and two output data for the DL approach. In this case, we used a sequential model as the linear stack of layers, where we passed a list of layers to the constructor. The designed model satisfied the following technical specifications.

- The first layer, called the input layer, has an input shape to set an input size of 5 that matches the training data.
- Next, weights of the output shape of dense layers of 10, 50, 100, and 50 of the ReLU activation function are updated (Figure 3).
- In another DL model, weights of the output shape of dense layers of 10, 25, 50, 100, 100, 50, 25, and 10 of the ReLU, Swish, and Leaky ReLU activation functions are updated (Figure 4).
- Finally, the output layer has a weight update of two units of the ReLU dense layers.

#### 2.3.3. Training of Model

We trained a classifier on the trained data, tuned the data using the validation set, and evaluated the final performance on the test data-set. Initially, by training the data with 20 epochs, we obtained training and validation losses from the single train-validation-test split technique of training and test data for the threshold value of $10^{-5}$ (in case of the ReLU function). We used a simple algorithm to develop a DL model (Figure 3). However, this single-splitting technique caused overfitting and noise inside data samples. As a result, we designed another DL algorithm (Figure 4) for the dense layers of ReLU, Leaky ReLU and Swish using single-to-five-fold cross validation technique. For all three different basic activation functions, we used the same DL algorithm. However, if the middle layers are varied, each model is different. These middle layers either removed or added to change the depth of the model and, tested the performance of each model with the effect of mid-size DL model. Moreover, we established each model to consider a small number of layers (five-to-seven layers) to avoid overfitting in data-set. Otherwise, it might show biased results due to overfitting. Therefore, we applied k-fold cross-validation to avoid overfitting and improve the average accuracy values by repeating the cross-validation of the single-fold to up to five-fold. We generated three models for each of three basic activation functions by varying internal middle layers of the proposed algorithm and, got total nine average accuracy values from them (using the trial-and-error method). The test data were compared with the predicted DL model within the set threshold value of $6\times 10^{-5}$ in each case. We trained our model with epochs [37,38] of 20 and a batch size [39,40] of 50.

## 3. Results

### 3.1. Concentration Profiles of Different Microflow Regions Revealed from Simulations

**Figure 5:**
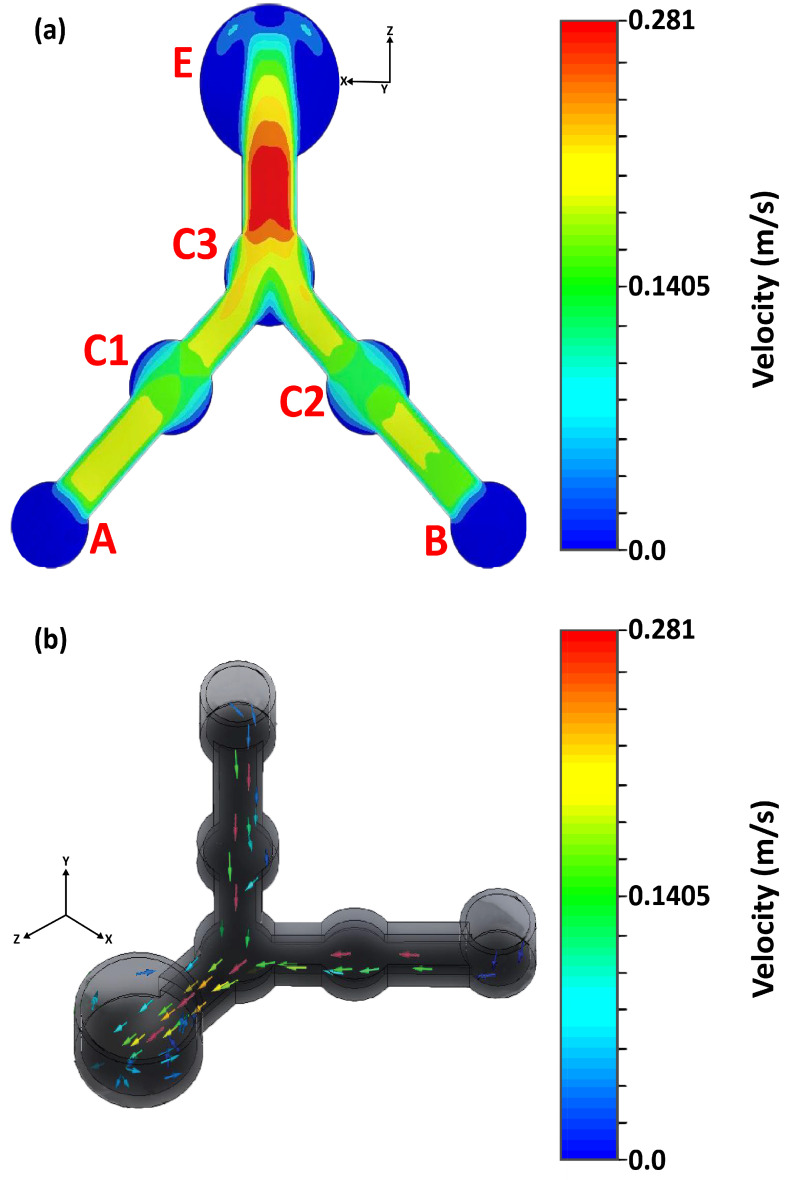
(**a**) Contour image of velocity field of two multi-phases pH 4 and 10 buffer solutions for the 100 μm microchannels. (**b**) Isometric three-dimensional flow dynamics of distributed charged particles through the microchannels path.

At the two inlet wells, the same set values of volumetric fluid flow rates (for example, 1.82 mL/min at the two inlets) were applied as inputs to determine the mixture of the multiphase flow of fluid patterns in terms of the velocity profile through 100 µm rectangular microflow channels. The objective of this optimization is to determine the charge particle distribution in different regions of microflow channels by revealing concentration profiles in terms of the velocities of the mixture of the two pH buffer fluids. A slight chaotic mixing that started at the end of the junction point, C3, towards the gateway of outlet, E, was observed (Figure 5a). Nevertheless, the two pH buffer fluids exhibited gentle behavior up to the middle of C3 from the two inlet points. At the junction point, the two pH buffer fluids transferred heat, temperature, energy, and other parameters according to thermodynamics principles, resulting in a somewhat complex mixture of both fluids. The flow dynamics of the charged particle distribution obtained from the simulation results are shown in Figure 5b.

**Figure 6:**
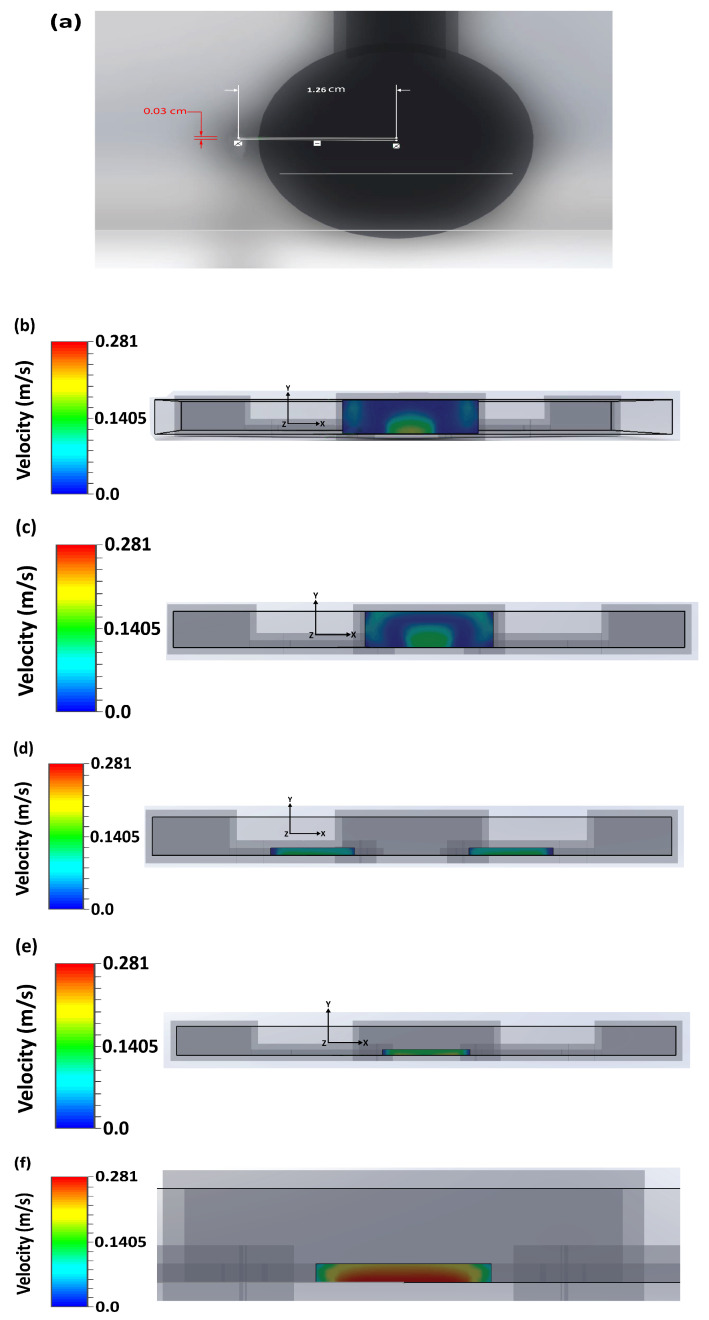
(**a**) The origin (0, 0) location for reference from the center point of the outlet well of the simulated 3D microfluidic structure to determine distances of different cross-sectional portions of microchannels. Directions away from and toward this origin mean positive and negative distances respectively. (**b**) (0, 0) origin point- cross-section of the portion of outlet. (**c**) 0.04 cm positive distance with respect to the origin- cross-section of the portion of outlet. (**d**) 0.28 cm negative distance with respect to the origin- cross-section of the rectangular common path. (**e**) 0.35 cm negative distance with respect to the origin- cross-section of rectangular two inlet paths. (**f**) 0.155 cm positive distance with respect to the origin- cross-section of the portion of the outlet.

In the simulations, the center point of the outlet well of the microfluidic structure was indicated as the origin location for reference. From this reference, different local microchannel regions were cross-sectioned to produce different flow-field concentrations in terms of the mixture velocity of the two multiphase pH buffer fluids. The cross-sectional diagrams of different regions of the microflow channels with the reference point are shown in Figure 6.

### 3.2. Dense Layers of ReLU, Swish, and Leaky ReLU Functions to Test Training Data Accuracy with Applied k-fold Cross-Validation

**Figure 7:**
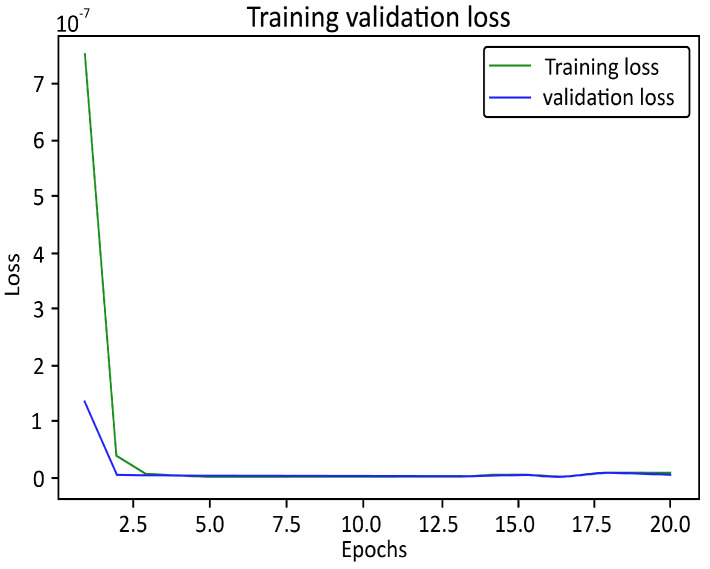
The Graph of training and validation losses with respect to twenty (20) number of epochs (Applying single train-validation-test split technique to DL model of Figure 3) in case of ReLU basic function.

At the development of small epochs, the losses for the test data exhibited a difference for the single train-validation-test split technique (Figure 7). Here, we applied DL model of Figure 3. However, in our generated model, with the progress of larger epochs up to 20, the plot of training and validation losses started to reach stability points with a narrow or no gap between them. This confirmed that the plot of these two learning curves showed a good fit. However, continuous training of a good fit led to overfitting, generated noise in the data-set. It might have drawbacks in model fitting or data-set which obtained from the measurement and interpolation of data. Therefore, we decided to apply the k-fold cross-validation technique to increase the maximum average accuracy of 99.81% to prevent overfitting and minimize noise in the data-set. In this technique, we trained and tested three different models for each of ReLU, Leaky ReLU and Swish of the same DL algorithm (Figure 4), by varying (adding or removing) internal middle layers of that designed algorithm. As a result, using trial-and-error method, we obtained total 9 average accuracy values of the above-mentioned three basic activation functions for the set threshold value of $6\times 10^{-5}$. The comparison chart of ReLU, Leaky ReLU and Swish activation functions with the obtained average accuracy from three different models is given in Table 1.

The chart suggests that the generated third model of Leaky ReLU achieved maximum average accuracy of 99.81% among all basic functions. Moreover, Leaky ReLU showed good percentage of average accuracy for each of model 2 and 3. While ReLU and Swish had the worst average accuracy values for the third model. Overall, Leaky ReLU showed satisfactory results for all generated models.

The computer code is shown in the reference [33] using a URL link and in the Supplementary Materials section too. In addition, raw measurement and interpolation of data samples with the processed data of microfluidic parameters and some of graphs "loss versus epochs" with their corresponding data are available. Moreover, 9 average accuracy values data are also available (see Supplementary Materials).

## 4. Discussion

**Figure 8:**
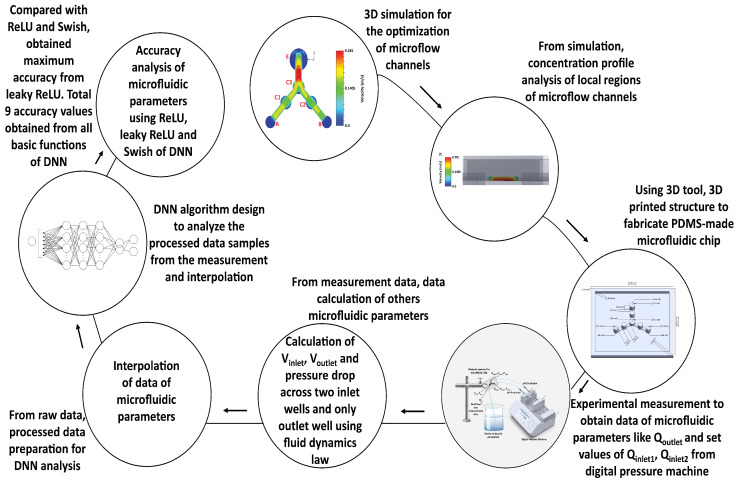
Flow diagram of simulation, optimization, 3D-printing, experimental measurement, calculation of data of microfluidic parameters and DNN algorithm design to analyze the average accuracy of such data of microfluidic parameters.

We illustrated the flow diagram of procedures of this work to help the readers to understand the simulation, experiments and programming steps for evaluating the average accuracy of a microfluidic system (Figure 8). For the 100 µm microflow channels of the designed microfluidic chip, we simulated the flow-field patterns in terms of the velocities of two multiphase pH buffer fluids. Both fluids were mixed at a junction with a transfer of temperature, energy, chemical kinetics, and other properties. The simulations were run in an intensive 3D environment free from external atmospheric pressure. We found that the maximum gained velocity at the end of junction point C3 towards the gateway of outlet well E resulted from the mixture of the two buffer fluids (Figure 5a). Moreover, the simulations help clarify the isometric 3D flow dynamics of the charged particle distribution within different internal regions of the microflow channels (Figure 5b). In addition, we analyzed the concentration profiles of different local regions of the internal microflow channels of the microfluidic chip, providing guidelines for optimizing microflow channels during the final design and printing processes.

Next, we measured the volumetric fluid flow rates at the outlet well and determined microfluidic resistances, average velocities and pressure drops across two inlets and the outlet well of the designed microfluidic chip. Then, the raw measurement data processed with the interpolation of data samples. Consequently, our processed data-set consisted of 1600 samples for the analysis of three basic activation functions (ReLU, Leaky ReLU, and Swish) of DNN. Initially, a single train-validation-test split technique utilized to design a simplified DL model (Figure 3) for the ReLU basic activation function. However, using that data-split technique, for such a limited data-set, a large amount of noise generated with overfitting, which depended on how the training and test data were partitioned. To overcome this difficulty, we applied k-fold cross-validation in which the entire data-set was partitioned into two or more folds. In this study, single-to-five-folds approach applied. We evaluated the best-fit model for the test data-set using the single-to-five fold cross-validation process. Using trial-and-error method, we changed or removed internal middle dense layers of the same applied DL algorithm (Figure 4) for the setting threshold value of $6\times 10^{-5}$. By such internal changing, we found 3 average accuracy values from three different models for each of ReLU, Leaky ReLU and Swish activation functions. Thus, after training the data samples with the tested data samples within our designed DL model, we obtained 9 average accuracy values for all activation functions, from which Leaky ReLU showed comparatively maximum accuracy values for third established model. The maximum accuracy of Leaky ReLU was 99.81%. Moreover, Leaky ReLU generated comparatively good percentages of average accuracy values for all three generated models. Such an investigation is crucial for revealing the exact input data-set corresponding to the output data-set of microfluidic parameters based on the applied DL model. Despite the limited number of data-set, the DL approach can provide future guidelines to train and test more data samples to compare the accuracy, validation and training losses for evaluating data of microfluidic parameters.

## 5. Conclusions

Microfluidics technology is driven by the application of different ML and DL neural network algorithms. In this study, based on the data analysis of microfluidic parameters for a defined threshold value of $6\times 10^{-5}$, we obtained a maximum average accuracy of 99.81% for all single fold to five folds of the Leaky ReLU function from the third generated model among all three models. In contrast, ReLU and Swish showed poor accuracy in case of each of third generated models. Overall, the performance of Leaky ReLU for such evaluation of data of microfluidic parameters was excellent. The above DL analysis of microfluidics will promote high-throughput screening of cancer cells for cell therapy and regenerative medicine, point-of-care analysis of automated blood-cell detection, in vivo and in vitro counting using organ-on-chips, contamination assessment of pH-controlled food samples, and pH-level testing of blood samples in biomedical and chemical research. In future studies, we plan to perform high-throughput sequencing of different biological and chemical live-saving samples using microflow channels and analyze their flow dynamics. Moreover, different images of flow-field patterns will be captured using a 3D simulation tool and terahertz laser scanning to analyze several images of fluid flow patterns with different DL model algorithms. Thus, we hope to develop imaging flow cytometry-directed microfluidic technology to analyze a wide range of laser-generated imaging data using a deep neural network to classify human and animal cells in the biomedical industry.

## Figures and Tables

**Table 1:** Average Accuracy chart of ReLU, Leaky ReLU and Swish functions of DNN, obtained from the addition or removal of internal layers of the same applied DL algorithm.

Defined Threshold Value	Basic Activation Functions	Average Accuracy (Model 1)	Average Accuracy (Model 2)	Average Accuracy (Model 3)
	ReLU	91.68%	88.74%	62.06%
$6\times 10^{-5}$	Leaky ReLU	94.56%	99.44%	99.81%
	Swish	86.80%	94.62%	51.25%

## Data Availability

Data are contained within the article or Supplementary Materials. The data presented in this study are available as Supplementary Materials according to Research Data Policies” at https://www.mdpi.com/ethics.

## Supplementary Materials

The following are available online at https://www.mdpi.com/article/10.3390/mi13081352/s1, Excel document S1: First data file Raw masurement data of Microfluidic parameters, PDF document S1: second data file Processed Data of Microfluidic parameters, PDF document S2: Computer Code-ML in microfludics simulations ipynb-Colaboratory, PDF document S3: data and corresponding graphical analysis of loss versus epochs of ReLU, Swish and leaky ReLU, Folder S1: Leaky ReLU data of generated three models, Folder S2: Leaky ReLU data of generated three models, Folder S3: Swiss model data of generated three models.

## Abbreviations

The following abbreviations are used in this manuscript:

ReLU	Rectified Linear Unit
Leaky ReLU	Leaky Rectified Linear Unit
DL	Deep Learning
ML	Machine Learning
DNN	Deep Neural Network
PDMS	Polydimethylsiloxane
