# Supplementary material for: Optimization of Microchannels and Application of Basic Activation Functions of Deep Neural Network for Accuracy Analysis of Microfluidic Parameter Data
Source: Micromachines (Basel). 2022 Aug 20;13(8):1352. doi: 10.3390/mi13081352 (PMC9413860; doi:10.3390/mi13081352)
Supplement: Supplementary file 1 [file micromachines-13-01352-s001.zip › Computer Code-ML in microfludics simulations ipynb-Colaboratory.pdf]

```
import os
import numpy as np
import pandas as pd

import tensorflow as tf
from tensorflow.python.keras.layers import Input, Dense                        # for model generation
from tensorflow.python.keras.models import Model                               # for model generation
from sklearn.model_selection import train_test_split                           # for test data and train data separation
from sklearn.model_selection import KFold, StratifiedKFold                     # For cross validation check
from tensorflow.random import set_seed                                         # for setting seed
#set_seed(1234)
import matplotlib.pyplot as plt
from sklearn.model_selection import KFold                                      # For k-fold cross validation


import time

def current_milli_time():
    return round(time.time() * 1000)
```

Now our aim is to predict the possible inputs for desired output parameters.

Therefore, real_time_input will be the output for this ML system and real_time_output will be the input.

```
#data = pd.read_csv('ML_in_microfludics_data.csv') # importing the data. Already in the drive folder. Just add into to files
data = pd.read_csv('400_new_formatted_data.csv') # importing the data. Already in the drive folder. Just add into to files

data.dtypes                                        #Checking data types of the columns

     C1                      float64
     Cout                    float64
     v (Inlets)              float64
     Pressure at Inlet1      float64
     pressure at Inlet2      float64
```

```
v(outlet)             float64
pressure drop         float64
dtype: object
```

```
data.head()
```

	C1	Cout	v (Inlets)	Pressure at Inlet1	pressure at Inlet2	v(outlet)	pressure drop
**0**	1.667000e-08	3.167300e-08	0.000003	2.318000e-13	2.737000e-13	0.000005	2.619000e-13
**1**	3.334000e-08	6.334600e-08	0.000006	4.637000e-13	5.474000e-13	0.000009	5.238000e-13
**2**	5.001000e-08	9.501900e-08	0.000009	6.956000e-13	8.211000e-13	0.000014	7.858000e-13
**3**	6.668000e-08	1.300260e-07	0.000012	9.275000e-13	1.094800e-12	0.000019	1.075300e-12
**4**	8.335000e-08	1.633660e-07	0.000016	1.159300e-12	1.368600e-12	0.000024	1.351000e-12

```
real_time_output = data[['v (Inlets)' , 'Pressure at Inlet1' ,  'pressure at Inlet2' , 'v(outlet)', 'pressure drop']].copy()
#real_time_output.head()
real_time_input = data[['C1' ,  'Cout' ]].copy()
#real_time_input.head()
```

```
seed_value = 100
threshold_value = 6*10**-5     #threshold value to compate the prediction and actual value


#my_activation_func = 'LeakyReLU'
my_activation_func = 'ReLU'
my_optimizer = 'adam'

#Implementing cross validation

k = 5 #Number of folds
kf = KFold(n_splits=k, shuffle = True, random_state=1)
```

```
my_epoch = 20 # number of epochs in training
my_batch_size = 50 # number of batch in training

input1 = Input(shape=(5,))
l1 = Dense(10, activation = my_activation_func)(input1)
l2 = Dense(25, activation = my_activation_func)(l1)
l3 = Dense(50, activation = my_activation_func)(l2)
l4 = Dense(100, activation = my_activation_func)(l3)
l5 = Dense(100, activation = my_activation_func)(l4)
l6 = Dense(50, activation = my_activation_func)(l5)
l7 = Dense(25, activation = my_activation_func)(l6)
l8 = Dense(10, activation = my_activation_func)(l7)
out = Dense(2)(l8)

def custom_loss_function(y_true, y_pred):
   squared_difference = tf.abs(tf.square(y_true - y_pred))
   return tf.reduce_mean(squared_difference,  axis=-1)

#model.compile(optimizer='adam', loss=custom_loss_function)



model = Model(inputs=input1, outputs=[out])

model.compile(optimizer = my_optimizer,
              loss=custom_loss_function)




acc_score = []
epoch_loss =[]
for train_index , test_index in kf.split(real_time_output):
    x_train , x_test = real_time_output.iloc[train_index,:],real_time_output.iloc[test_index,:]
    y_train , y_test = real_time_input.iloc[train_index,:] , real_time_input.iloc[test_index,:]

    test_x_dim = x_test.shape[0]
    print('Test sample size {}'.format(test_x_dim))
    history = model.fit(x_train, y_train, epochs = my_epoch, batch_size = my_batch_size)
```

```
        pred = model.predict(x_test)

        abs_diff = np.absolute(np.array(y_test) - np.array(pred)) #final accuracy is calculated based on absolute difference betw
        comparison_result = abs_diff <= threshold_value
        df = pd.DataFrame(comparison_result) #, dtype='float64')

        count = 0
        for i in range(test_x_dim):
    #    if np.sum(df[0][i]) + np.sum(df[0][i]) == 2:
            if np.sum(df[0][i]) + np.sum(df[1][i]) == 2: # both the input parameters deviation must be within threshold value
                #print(df[0][i],df[0][i])
                count = count + 1
        accuray = (count/test_x_dim)*100
        acc_score.append(accuray)
        epoch_loss.append(history.history['loss'])

    avg_acc_score = sum(acc_score)/k
    print('Activation functions: {}'.format(my_activation_func))
    print('Optimizer: {}'.format(my_optimizer))
    print('Epochs = {}, Batch size = {}'.format(my_epoch, my_batch_size))
    print('Threshold value = {}'.format(threshold_value))
    print('Number of folds = {}'.format(k))
    print('Accuracy of each fold : {}'.format(acc_score))
    print('Avg accuracy : {0:7.2f} %'.format(avg_acc_score))
    epoch_loss_array = np.array(epoch_loss)
    epoch_loss_array.shape
    #print(epoch_loss_array)

    text_file_name = my_activation_func + '_' + my_optimizer + '_epoch_' + str(my_epoch) + '_batch_' + str(my_batch_size)+'__'+ s
    with open(text_file_name, "w") as variable_file:
        variable_file.write('Activation functions: {}\n'.format(my_activation_func))
        variable_file.write('Optimizer: {}\n'.format(my_optimizer))
        variable_file.write('Epochs = {}, Batch size = {}\n'.format(my_epoch, my_batch_size))
        variable_file.write('Threshold value = {}\n'.format(threshold_value))
        variable_file.write('Number of folds = {}\n'.format(k))
        variable_file.write('Accuracy of each fold : {}\n'.format(acc_score))
        variable_file.write('Avg accuracy : {0:7.2f} %\n'.format(avg_acc_score))
```

```
    variable_file.write('Epoch loss : \n')
    variable_file.write(str(epoch_loss_array))
```

```
Test sample size 320
Epoch 1/20
26/26 [==============================] - 1s 2ms/step - loss: 2.2394e-07
Epoch 2/20
26/26 [==============================] - 0s 2ms/step - loss: 8.2417e-09
Epoch 3/20
26/26 [==============================] - 0s 2ms/step - loss: 1.1342e-09
Epoch 4/20
26/26 [==============================] - 0s 2ms/step - loss: 5.4757e-10
Epoch 5/20
26/26 [==============================] - 0s 2ms/step - loss: 3.4211e-10
Epoch 6/20
26/26 [==============================] - 0s 2ms/step - loss: 3.4015e-10
Epoch 7/20
26/26 [==============================] - 0s 3ms/step - loss: 9.5877e-10
Epoch 8/20
26/26 [==============================] - 0s 3ms/step - loss: 3.9606e-10
Epoch 9/20
26/26 [==============================] - 0s 2ms/step - loss: 4.4072e-10
Epoch 10/20
26/26 [==============================] - 0s 3ms/step - loss: 2.7964e-10
Epoch 11/20
26/26 [==============================] - 0s 2ms/step - loss: 1.0374e-09
Epoch 12/20
26/26 [==============================] - 0s 2ms/step - loss: 1.5685e-09
Epoch 13/20
26/26 [==============================] - 0s 3ms/step - loss: 7.8917e-10
Epoch 14/20
26/26 [==============================] - 0s 2ms/step - loss: 6.8454e-09
Epoch 15/20
26/26 [==============================] - 0s 3ms/step - loss: 3.2274e-10
Epoch 16/20
26/26 [==============================] - 0s 2ms/step - loss: 3.0709e-10
Epoch 17/20
26/26 [==============================] - 0s 2ms/step - loss: 5.7347e-10
Epoch 18/20
26/26 [==============================] - 0s 2ms/step - loss: 2.0712e-09
Epoch 19/20
```

```
26/26 [==============================] - 0s 2ms/step - loss: 3.6267e-09
Epoch 20/20
26/26 [==============================] - 0s 2ms/step - loss: 1.2191e-09
Test sample size 320
Epoch 1/20
26/26 [==============================] - 0s 3ms/step - loss: 4.4411e-09
Epoch 2/20
26/26 [==============================] - 0s 2ms/step - loss: 2.5467e-09
Epoch 3/20
26/26 [==============================] - 0s 2ms/step - loss: 3.4242e-09
Epoch 4/20
26/26 [==============================] - 0s 2ms/step - loss: 2.2612e-09
Epoch 5/20
26/26 [==============================] - 0s 2ms/step - loss: 1.0601e-09
Epoch 6/20
26/26 [==============================] - 0s 2ms/step - loss: 9.7217e-09
Epoch 7/20
26/26 [==============================] - 0s 3ms/step - loss: 1.1915e-09
Epoch 8/20
26/26 [==============================] - 0s 2ms/step - loss: 1.2770e-09
```

```
def custom_loss_function(y_true, y_pred):
   squared_difference = tf.abs(tf.square(y_true - y_pred))
   return tf.reduce_mean(squared_difference, axis=-1)
```
