# Supplementary material for: Optimization of Microchannels and Application of Basic Activation Functions of Deep Neural Network for Accuracy Analysis of Microfluidic Parameter Data
Source: Micromachines (Basel). 2022 Aug 20;13(8):1352. doi: 10.3390/mi13081352 (PMC9413860; doi:10.3390/mi13081352)
Supplement: Supplementary file 1 [file micromachines-13-01352-s001.zip › data and corresponding graphical analysis of loss versus epochs of ReLU, Swish and leaky ReLU.pdf]

Activation functions: ReLU
Optimizer: adam
Epochs = 20, Batch size = 50
Threshold value = 6.000000000000001e-05
Number of folds = 5
Accuracy of each fold : [94.0625, 95.625, 90.0, 83.125, 80.87774294670847]
Avg accuracy :   88.74 %
Epoch loss :

```
[[1.43912644e-07 2.30881159e-09 8.20735147e-10 7.32679362e-10
  6.95755620e-10 7.52224116e-10 7.18429150e-10 7.36637862e-10
  9.87575022e-10 8.11523737e-10 7.57603869e-10 7.80097209e-10
  8.71913319e-10 8.02208466e-10 8.38822123e-10 9.81887904e-10
  8.44503856e-10 7.98915989e-10 9.59670454e-10 8.79881001e-10]
 [9.20226340e-10 1.02897035e-09 9.53670032e-10 1.02933162e-09
  8.93908558e-10 8.51695103e-10 9.08418563e-10 9.93159333e-10
  9.95044491e-10 8.14046275e-10 8.10681022e-10 9.50450829e-10
  8.85170881e-10 1.01984821e-09 1.81295623e-09 9.56080326e-10
  8.82722229e-10 1.18189891e-09 1.43924450e-09 1.72634362e-09]
 [1.00694586e-09 1.57514513e-09 1.41166290e-09 9.80392212e-10
  1.24042532e-09 1.27969746e-09 1.11848952e-09 9.60430402e-10
  8.30464308e-10 1.04449660e-09 1.33514866e-09 1.80004811e-09
  1.96936267e-09 1.38289336e-09 9.87489202e-10 1.13652443e-09
  1.11720089e-09 1.82218651e-09 1.25352240e-09 1.17322563e-09]
 [1.64835923e-09 1.32522671e-09 9.80119208e-10 1.59290336e-09
  1.08791653e-09 9.91194238e-10 1.41636036e-09 8.69000427e-10
  1.35736800e-09 1.69866909e-09 9.15724385e-10 1.58547375e-09
  1.33924682e-09 1.04781217e-09 1.67258374e-09 1.15662391e-09
  2.06503215e-09 1.82651794e-09 1.28788735e-09 1.31795919e-09]
 [1.42979939e-09 1.92637661e-09 1.89518135e-09 1.30904332e-09
  1.34280953e-09 2.00823869e-09 2.33372033e-09 1.51275492e-09
  2.49071030e-09 2.40126652e-09 1.64365344e-09 2.26182029e-09
  1.35852463e-09 2.72981082e-09 1.36309464e-09 9.88669924e-10
  3.45107098e-09 1.06873410e-09 1.32895706e-09 1.64148362e-09]]
```

Activation functions: swish
Optimizer: adam
Epochs = 20, Batch size = 50
Threshold value = 6.000000000000001e-05
Number of folds = 5
Accuracy of each fold : [85.9375, 90.9375, 96.25, 100.0, 100.0]
Avg accuracy :   94.62 %
Epoch loss :

```
[[1.74692536e-06 1.07744839e-07 1.15971748e-08 3.45135254e-09
  2.30695729e-09 2.16227103e-09 2.08314099e-09 2.09102602e-09
  1.99504302e-09 2.02423589e-09 1.87756033e-09 1.74836245e-09
  1.92813809e-09 1.75382986e-09 1.80870086e-09 1.57884994e-09
  1.63424052e-09 1.77464321e-09 2.28709496e-09 2.06270445e-09]
 [1.66249159e-09 1.86933602e-09 1.47093726e-09 1.71003223e-09
  1.27030897e-09 1.11931764e-09 1.35133105e-09 1.21673893e-09
  1.38716338e-09 1.23186306e-09 9.74307302e-10 9.48186418e-10
  8.69935568e-10 9.58515711e-10 8.47374337e-10 7.90227161e-10
  9.20618581e-10 6.68934796e-10 8.56800464e-10 6.56770083e-10]
 [6.96704028e-10 6.47408904e-10 8.18665469e-10 1.32740119e-09
  8.07701184e-10 7.19313553e-10 6.21430574e-10 7.33309136e-10
  5.88168403e-10 4.14517948e-10 4.94349894e-10 6.22199958e-10
  6.89675317e-10 5.56615143e-10 1.24629229e-09 1.75233605e-08
  1.43617296e-08 1.78312354e-09 6.35654085e-10 6.18392559e-10]
 [2.83368640e-09 3.25942757e-08 2.96304759e-09 1.42383028e-09
  1.08118614e-09 4.40575398e-09 3.29207883e-09 4.83579541e-08
  4.78155515e-09 3.08012699e-10 6.19412410e-10 1.27321964e-09
  1.28314426e-09 2.24587566e-08 5.16183052e-09 1.71034265e-09
  1.80438420e-09 4.21632471e-08 4.36889769e-09 7.57979846e-10]
 [6.42944531e-10 1.99916572e-09 1.62801967e-08 4.69098893e-09
  6.83487977e-09 8.73709904e-09 7.37049444e-09 1.62079914e-08
  7.94390442e-10 1.38879797e-08 2.88294952e-08 1.59656488e-09
  4.82717533e-10 6.95485780e-10 1.13187382e-09 4.50803341e-08
  6.56674093e-09 4.92028696e-10 2.23689178e-10 6.63232691e-10]]
```

Activation functions: LeakyReLU
Optimizer: adam
Epochs = 20, Batch size = 50
Threshold value = 6.000000000000001e-05
Number of folds = 5
Accuracy of each fold : [97.1875, 100.0, 100.0, 100.0, 100.0]
Avg accuracy :   99.44 %
Epoch loss :

```
[[2.09846485e-06 5.46298864e-08 5.27129806e-09 1.40650624e-09
  9.38074507e-10 7.40518591e-10 6.11458606e-10 5.27373256e-10
  4.98341868e-10 4.33248798e-10 4.60301158e-10 3.82671367e-10
  3.41886935e-10 3.10995868e-10 2.98574387e-10 2.71462769e-10
  2.40231696e-10 2.27829713e-10 2.37672770e-10 2.02082684e-10]
 [1.87991164e-10 1.62504510e-10 1.44311008e-10 1.34997805e-10
  1.24038002e-10 1.46883200e-10 1.37843334e-10 1.03320359e-10
  9.44508846e-11 9.20776164e-11 9.84781770e-11 8.43122377e-11
  6.97836650e-11 7.46765358e-11 6.71865411e-11 7.13883536e-11
  5.67014942e-11 6.00941033e-11 5.37779855e-11 5.49194822e-11]
 [5.13886433e-11 4.54023590e-11 4.43406353e-11 4.29045098e-11
  3.85974690e-11 4.45658649e-11 3.66850821e-11 3.91320240e-11
  3.39113147e-11 3.63653344e-11 3.62440078e-11 2.90825194e-11
  3.33062987e-11 2.55102918e-11 2.20971106e-11 2.28434407e-11
  2.03287786e-11 2.05787210e-11 1.67919446e-11 1.61974722e-11]
 [1.54291319e-11 1.22793381e-11 1.20901717e-11 1.22746483e-11
  1.27270200e-11 1.64973972e-11 1.06695833e-11 1.10341380e-11
  1.62917891e-11 9.96122369e-12 1.08182525e-11 1.04707831e-11
  8.26819082e-12 7.68472092e-12 7.12959553e-12 1.29121653e-11
  8.53801231e-12 1.13565190e-11 9.16899109e-12 6.22415791e-12]
 [1.08354358e-11 9.19755071e-12 1.17280118e-11 1.65852106e-11
  8.75476497e-11 5.22069610e-11 2.08130446e-09 2.16234586e-09
  3.17143201e-10 6.26422358e-10 1.81220494e-09 3.89428445e-09
  1.56716098e-10 1.73736650e-10 6.70213973e-09 6.70541234e-10
  2.84806206e-10 1.90574709e-10 9.11140141e-09 2.32632869e-09]]
```

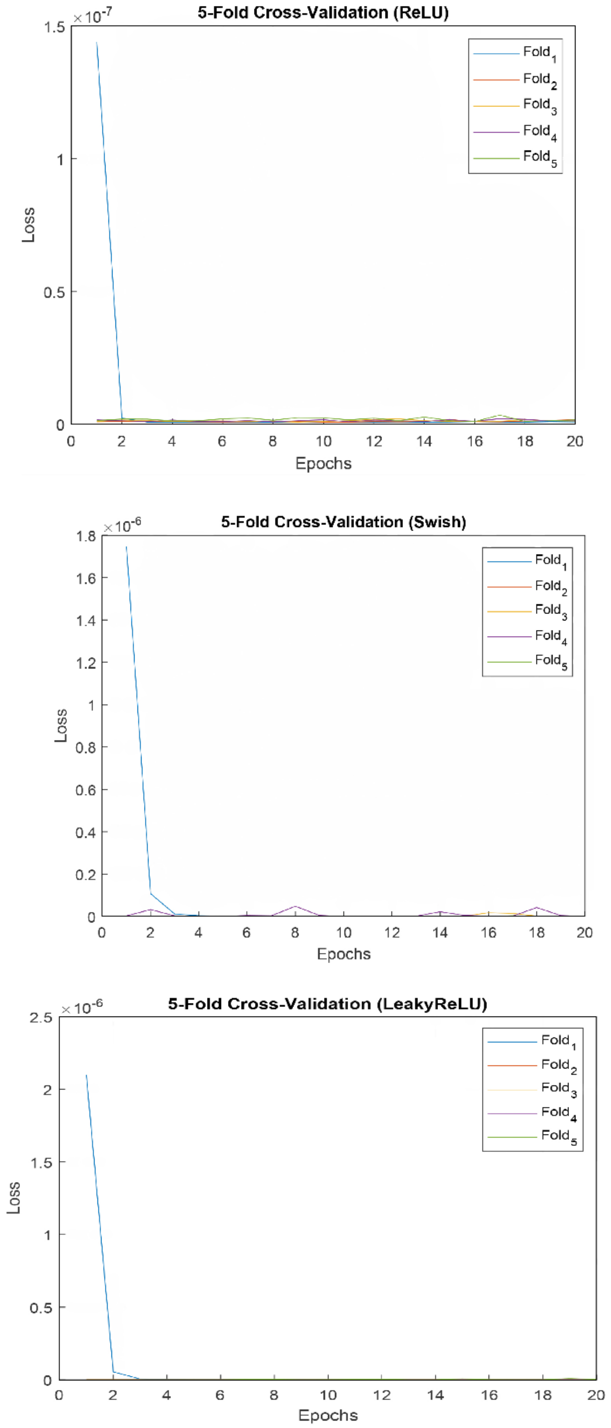

Fig. S1. By the application of single-to-five-fold cross-validation, for training and test data samples, each validation applied for twenty (20) epochs. Validation loss curves for the dense layers of (a) the ReLU with an accuracy of 88.74% (b) Swish with an accuracy of 94.62%, and (c) leaky ReLU with an accuracy of 99.44%, three basic activation functions

Based on the obtained results for the percentages of average accuracy values of the training data, the performance was significant in case of the applied single-to-five-fold cross-validation without overfitting, achieving accuracies of 88.74%, 94.62%, and 99.44% for ReLU, Swish, and leaky ReLU, respectively (Figure S1(a), (b), and (c)). These results suggest that the configured model satisfactorily learned the problem and generalized the solution. The sample data used in this study required high floating-point precision; otherwise, the results might have varied significantly. With an increasing number of epochs up to 20 from single to five-folds, all folds showed slight variations or almost coincided in several positions in the loss calculation, except when some small epochs developed for the first fold (Figure S1(a)). Except for the start of a few epochs in the first fold, only the fourth fold showed several variations compared to other folds with an increasing number of epochs, and the third fold showed variation in one position (Figure S1(b)). The remaining folds showed no gap or error as the number of epochs increased (Figure S1(b)). Except for the start of a small number of epochs in the first fold, all folds attained stability without any gap or error with the applied leaky ReLU functions as the number of epochs increased (Figure S1(c)). In all cases, losses tended to decrease during the start of a few epochs in the first fold. Overall, the leaky ReLU functions showed maximum accuracy with increasing the number of epochs without any gaps or errors.
