# Supplementary material for: Optimization of Microchannels and Application of Basic Activation Functions of Deep Neural Network for Accuracy Analysis of Microfluidic Parameter Data
Source: Micromachines (Basel). 2022 Aug 20;13(8):1352. doi: 10.3390/mi13081352 (PMC9413860; doi:10.3390/mi13081352)
Supplement: Supplementary file 1 [file micromachines-13-01352-s001.zip › ML_MODEL_2_LeakyReLU_adam_epoch_20_batch_50__threshold 6e-5.pdf]

Activation functions: LeakyReLU
Optimizer: adam
Epochs = 20, Batch size = 50
Threshold value = 6.000000000000001e-05
Number of folds = 5
Accuracy of each fold : [97.1875, 100.0, 100.0, 100.0, 100.0]
Avg accuracy :   99.44 %
Epoch loss :

```
[[2.09846485e-06 5.46298864e-08 5.27129806e-09 1.40650624e-09
  9.38074507e-10 7.40518591e-10 6.11458606e-10 5.27373256e-10
  4.98341868e-10 4.33248798e-10 4.60301158e-10 3.82671367e-10
  3.41886935e-10 3.10995868e-10 2.98574387e-10 2.71462769e-10
  2.40231696e-10 2.27829713e-10 2.37672770e-10 2.02082684e-10]
 [1.87991164e-10 1.62504510e-10 1.44311008e-10 1.34997805e-10
  1.24038002e-10 1.46883200e-10 1.37843334e-10 1.03320359e-10
  9.44508846e-11 9.20776164e-11 9.84781770e-11 8.43122377e-11
  6.97836650e-11 7.46765358e-11 6.71865411e-11 7.13883536e-11
  5.67014942e-11 6.00941033e-11 5.37779855e-11 5.49194822e-11]
 [5.13886433e-11 4.54023590e-11 4.43406353e-11 4.29045098e-11
  3.85974690e-11 4.45658649e-11 3.66850821e-11 3.91320240e-11
  3.39113147e-11 3.63653344e-11 3.62440078e-11 2.90825194e-11
  3.33062987e-11 2.55102918e-11 2.20971106e-11 2.28434407e-11
  2.03287786e-11 2.05787210e-11 1.67919446e-11 1.61974722e-11]
 [1.54291319e-11 1.22793381e-11 1.20901717e-11 1.22746483e-11
  1.27270200e-11 1.64973972e-11 1.06695833e-11 1.10341380e-11
  1.62917891e-11 9.96122369e-12 1.08182525e-11 1.04707831e-11
  8.26819082e-12 7.68472092e-12 7.12959553e-12 1.29121653e-11
  8.53801231e-12 1.13565190e-11 9.16899109e-12 6.22415791e-12]
 [1.08354358e-11 9.19755071e-12 1.17280118e-11 1.65852106e-11
  8.75476497e-11 5.22069610e-11 2.08130446e-09 2.16234586e-09
  3.17143201e-10 6.26422358e-10 1.81220494e-09 3.89428445e-09
  1.56716098e-10 1.73736650e-10 6.70213973e-09 6.70541234e-10
  2.84806206e-10 1.90574709e-10 9.11140141e-09 2.32632869e-09]]
```
