# Supplementary material for: Optimization of Microchannels and Application of Basic Activation Functions of Deep Neural Network for Accuracy Analysis of Microfluidic Parameter Data
Source: Micromachines (Basel). 2022 Aug 20;13(8):1352. doi: 10.3390/mi13081352 (PMC9413860; doi:10.3390/mi13081352)
Supplement: Supplementary file 1 [file micromachines-13-01352-s001.zip › ML_MODEL_2_ReLU_adam_epoch_20_batch_50__threshold 6e-5.pdf]

Activation functions: ReLU
Optimizer: adam
Epochs = 20, Batch size = 50
Threshold value = 6.000000000000001e-05
Number of folds = 5
Accuracy of each fold : [94.0625, 95.625, 90.0, 83.125, 80.87774294670847]
Avg accuracy :   88.74 %
Epoch loss :

```
[[1.43912644e-07 2.30881159e-09 8.20735147e-10 7.32679362e-10
  6.95755620e-10 7.52224116e-10 7.18429150e-10 7.36637862e-10
  9.87575022e-10 8.11523737e-10 7.57603869e-10 7.80097209e-10
  8.71913319e-10 8.02208466e-10 8.38822123e-10 9.81887904e-10
  8.44503856e-10 7.98915989e-10 9.59670454e-10 8.79881001e-10]
 [9.20226340e-10 1.02897035e-09 9.53670032e-10 1.02933162e-09
  8.93908558e-10 8.51695103e-10 9.08418563e-10 9.93159333e-10
  9.95044491e-10 8.14046275e-10 8.10681022e-10 9.50450829e-10
  8.85170881e-10 1.01984821e-09 1.81295623e-09 9.56080326e-10
  8.82722229e-10 1.18189891e-09 1.43924450e-09 1.72634362e-09]
 [1.00694586e-09 1.57514513e-09 1.41166290e-09 9.80392212e-10
  1.24042532e-09 1.27969746e-09 1.11848952e-09 9.60430402e-10
  8.30464308e-10 1.04449660e-09 1.33514866e-09 1.80004811e-09
  1.96936267e-09 1.38289336e-09 9.87489202e-10 1.13652443e-09
  1.11720089e-09 1.82218651e-09 1.25352240e-09 1.17322563e-09]
 [1.64835923e-09 1.32522671e-09 9.80119208e-10 1.59290336e-09
  1.08791653e-09 9.91194238e-10 1.41636036e-09 8.69000427e-10
  1.35736800e-09 1.69866909e-09 9.15724385e-10 1.58547375e-09
  1.33924682e-09 1.04781217e-09 1.67258374e-09 1.15662391e-09
  2.06503215e-09 1.82651794e-09 1.28788735e-09 1.31795919e-09]
 [1.42979939e-09 1.92637661e-09 1.89518135e-09 1.30904332e-09
  1.34280953e-09 2.00823869e-09 2.33372033e-09 1.51275492e-09
  2.49071030e-09 2.40126652e-09 1.64365344e-09 2.26182029e-09
  1.35852463e-09 2.72981082e-09 1.36309464e-09 9.88669924e-10
  3.45107098e-09 1.06873410e-09 1.32895706e-09 1.64148362e-09]]
```
