# Supplementary material for: Optimization of Microchannels and Application of Basic Activation Functions of Deep Neural Network for Accuracy Analysis of Microfluidic Parameter Data
Source: Micromachines (Basel). 2022 Aug 20;13(8):1352. doi: 10.3390/mi13081352 (PMC9413860; doi:10.3390/mi13081352)
Supplement: Supplementary file 1 [file micromachines-13-01352-s001.zip › ML_MODEL_2_swish_adam_epoch_20_batch_50__1651857057257.pdf]

Activation functions: swish
Optimizer: adam
Epochs = 20, Batch size = 50
Threshold value = 6.000000000000001e-05
Number of folds = 5
Accuracy of each fold : [85.9375, 90.9375, 96.25, 100.0, 100.0]
Avg accuracy :   94.62 %
Epoch loss :

```
[[1.74692536e-06 1.07744839e-07 1.15971748e-08 3.45135254e-09
  2.30695729e-09 2.16227103e-09 2.08314099e-09 2.09102602e-09
  1.99504302e-09 2.02423589e-09 1.87756033e-09 1.74836245e-09
  1.92813809e-09 1.75382986e-09 1.80870086e-09 1.57884994e-09
  1.63424052e-09 1.77464321e-09 2.28709496e-09 2.06270445e-09]
 [1.66249159e-09 1.86933602e-09 1.47093726e-09 1.71003223e-09
  1.27030897e-09 1.11931764e-09 1.35133105e-09 1.21673893e-09
  1.38716338e-09 1.23186306e-09 9.74307302e-10 9.48186418e-10
  8.69935568e-10 9.58515711e-10 8.47374337e-10 7.90227161e-10
  9.20618581e-10 6.68934796e-10 8.56800464e-10 6.56770083e-10]
 [6.96704028e-10 6.47408904e-10 8.18665469e-10 1.32740119e-09
  8.07701184e-10 7.19313553e-10 6.21430574e-10 7.33309136e-10
  5.88168403e-10 4.14517948e-10 4.94349894e-10 6.22199958e-10
  6.89675317e-10 5.56615143e-10 1.24629229e-09 1.75233605e-08
  1.43617296e-08 1.78312354e-09 6.35654085e-10 6.18392559e-10]
 [2.83368640e-09 3.25942757e-08 2.96304759e-09 1.42383028e-09
  1.08118614e-09 4.40575398e-09 3.29207883e-09 4.83579541e-08
  4.78155515e-09 3.08012699e-10 6.19412410e-10 1.27321964e-09
  1.28314426e-09 2.24587566e-08 5.16183052e-09 1.71034265e-09
  1.80438420e-09 4.21632471e-08 4.36889769e-09 7.57979846e-10]
 [6.42944531e-10 1.99916572e-09 1.62801967e-08 4.69098893e-09
  6.83487977e-09 8.73709904e-09 7.37049444e-09 1.62079914e-08
  7.94390442e-10 1.38879797e-08 2.88294952e-08 1.59656488e-09
  4.82717533e-10 6.95485780e-10 1.13187382e-09 4.50803341e-08
  6.56674093e-09 4.92028696e-10 2.23689178e-10 6.63232691e-10]]
```
