# Supplementary material for: Optimization of Microchannels and Application of Basic Activation Functions of Deep Neural Network for Accuracy Analysis of Microfluidic Parameter Data
Source: Micromachines (Basel). 2022 Aug 20;13(8):1352. doi: 10.3390/mi13081352 (PMC9413860; doi:10.3390/mi13081352)
Supplement: Supplementary file 1 [file micromachines-13-01352-s001.zip › ML_MODEL_3_LeakyReLU_adam_epoch_20_batch_50__1651857330684.pdf]

Activation functions: LeakyReLU
Optimizer: adam
Epochs = 20, Batch size = 50
Threshold value = 6.000000000000001e-05
Number of folds = 5
Accuracy of each fold : [100.0, 100.0, 100.0, 100.0, 99.05956112852664]
Avg accuracy :   99.81 %
Epoch loss :

```
[[1.42531223e-06 4.41535235e-08 3.88240196e-09 7.35417449e-10
  5.21226173e-10 4.59449367e-10 4.24361268e-10 3.86952886e-10
  3.62127606e-10 3.43690965e-10 3.09678116e-10 2.68183170e-10
  2.44245152e-10 2.34855746e-10 2.16402160e-10 1.89556537e-10
  1.78521350e-10 1.67432762e-10 1.47312038e-10 1.36383210e-10]
 [1.19119742e-10 1.09012355e-10 9.89620053e-11 8.94610080e-11
  8.44252723e-11 7.10595541e-11 6.90381849e-11 7.35360453e-11
  5.37146265e-11 5.18714481e-11 4.80346735e-11 4.81630742e-11
  4.05791095e-11 3.78758934e-11 3.67528993e-11 3.25522699e-11
  3.43710511e-11 2.92308296e-11 2.47065597e-11 2.44718672e-11]
 [2.15228408e-11 2.16277968e-11 1.65979609e-11 1.38893272e-11
  1.25700457e-11 1.25557204e-11 9.21217703e-12 9.24169942e-12
  7.16156735e-12 7.20234246e-12 5.59521006e-12 6.08703322e-12
  4.86726544e-12 5.51656767e-12 4.57609688e-12 3.88068440e-12
  3.28974521e-12 3.29867557e-12 4.12370528e-12 2.97296346e-12]
 [2.88002846e-12 3.17949118e-12 3.10692162e-12 2.75537882e-12
  2.52141407e-12 2.23354473e-12 2.09150037e-12 2.05975493e-12
  2.01757729e-12 2.13617318e-12 3.22798212e-12 2.32801972e-12
  2.75481482e-12 2.35040828e-12 1.82555598e-12 2.08548608e-12
  2.57686971e-12 1.56907928e-12 1.47914710e-12 2.30812201e-12]
 [1.51468681e-12 1.47828679e-12 2.37417985e-12 2.13412578e-12
  1.32434287e-12 1.41218027e-12 3.45244146e-12 8.02243515e-12
  7.73225841e-12 1.64268078e-11 1.39769221e-11 1.27948320e-11
  1.60800373e-09 1.51609889e-08 9.98938376e-10 1.62707528e-10
  3.55603429e-11 1.69409667e-10 2.88628677e-08 2.79260304e-09]]
```
