# Supplementary material for: Optimization of Microchannels and Application of Basic Activation Functions of Deep Neural Network for Accuracy Analysis of Microfluidic Parameter Data
Source: Micromachines (Basel). 2022 Aug 20;13(8):1352. doi: 10.3390/mi13081352 (PMC9413860; doi:10.3390/mi13081352)
Supplement: Supplementary file 1 [file micromachines-13-01352-s001.zip › ML_MODEL_3_ReLU_adam_epoch_20_batch_50__1651857449560.pdf]

Activation functions: ReLU
Optimizer: adam
Epochs = 20, Batch size = 50
Threshold value = 6.000000000000001e-05
Number of folds = 5
Accuracy of each fold : [90.9375, 29.6875, 0.0, 100.0, 89.65517241379311]
Avg accuracy :   62.06 %
Epoch loss :

```
[[3.52605497e-07 9.63519575e-09 1.28825761e-09 4.96961750e-10
  4.18115126e-10 3.58460595e-10 3.35634354e-10 3.28528205e-10
  3.13436554e-10 3.31728728e-10 3.12250087e-10 3.04146569e-10
  3.06129705e-10 3.34523187e-10 2.88650992e-10 4.30524505e-10
  5.29290722e-10 1.23732713e-09 5.36156675e-09 1.80208215e-09]
 [3.12671578e-09 1.87908640e-08 1.18904842e-09 3.79223819e-10
  3.37684769e-10 4.88692808e-10 1.33253062e-08 5.12865883e-09
  6.87568724e-10 4.19948160e-10 3.08341852e-10 2.61364548e-08
  5.62677238e-09 8.91855312e-10 3.66963154e-10 3.65062508e-10
  3.77471582e-10 8.27960089e-10 1.46824526e-08 7.44801687e-09]
 [7.93241139e-10 4.13350909e-10 3.98814759e-10 3.62772479e-10
  2.23455618e-08 6.08027628e-09 8.14971535e-10 5.09855824e-10
  3.37909978e-10 2.72666473e-10 2.04160822e-09 1.20562094e-08
  1.54851676e-09 5.56816981e-10 1.16090693e-08 7.70833974e-09
  8.50577109e-10 3.66277536e-10 5.09367992e-10 1.88482936e-08]
 [6.85548729e-09 8.94653462e-10 3.32996408e-10 3.07346454e-10
  4.81490958e-10 1.15006360e-08 1.51303468e-08 1.45122059e-09
  4.95920582e-10 2.89176016e-10 2.57204591e-10 8.35286895e-10
  1.65164273e-08 4.52466242e-09 1.19937671e-09 6.84262702e-10
  8.57053983e-10 1.16699217e-09 5.55283597e-10 9.37740108e-10]
 [2.26283992e-09 4.60969662e-09 1.66826286e-09 2.65457922e-09
  8.17338552e-09 1.69286829e-09 1.44557633e-09 6.35946762e-09
  3.27028427e-09 1.02755116e-09 1.32341893e-09 1.98717931e-08
  1.71814263e-09 7.38872352e-10 5.12942633e-10 6.47518983e-10
  1.28068822e-09 1.13860228e-08 2.21990382e-09 5.93196714e-10]]
```
