# Supplementary material for: Optimization of Microchannels and Application of Basic Activation Functions of Deep Neural Network for Accuracy Analysis of Microfluidic Parameter Data
Source: Micromachines (Basel). 2022 Aug 20;13(8):1352. doi: 10.3390/mi13081352 (PMC9413860; doi:10.3390/mi13081352)
Supplement: Supplementary file 1 [file micromachines-13-01352-s001.zip › ML_MODEL_3_swish_adam_epoch_20_batch_50__1651857555271.pdf]

Activation functions: swish
Optimizer: adam
Epochs = 20, Batch size = 50
Threshold value = 6.000000000000001e-05
Number of folds = 5
Accuracy of each fold : [91.875, 65.0, 4.0625, 0.0, 95.29780564263322]
Avg accuracy :   51.25 %
Epoch loss :

```
[[1.43878606e-06 8.20776620e-08 7.78705633e-09 9.23744914e-10
  6.35314579e-10 6.06919737e-10 6.27242480e-10 6.07619455e-10
  8.08579648e-10 7.26372129e-10 7.84146470e-10 8.30112867e-10
  1.00317143e-09 9.51867918e-10 7.04410252e-10 6.71603162e-10
  1.80210813e-09 1.78731732e-08 3.03372438e-09 1.04328102e-09]
 [2.63682800e-08 4.22838564e-09 1.09319109e-09 4.60720839e-09
  5.61170266e-09 1.57055258e-08 5.65867397e-09 9.21676602e-09
  1.20454917e-08 1.37406619e-08 2.68343769e-09 5.07549691e-09
  1.03126121e-08 1.16012506e-08 5.35949463e-09 1.44528505e-08
  1.32715683e-09 3.23365406e-08 1.17481926e-08 2.97970426e-09]
 [2.18443197e-09 2.40971998e-09 1.45661572e-08 8.34788860e-09
  1.22683010e-08 1.88119254e-09 1.78243518e-08 1.30404771e-08
  6.78222944e-09 5.27613464e-09 2.66454014e-09 4.56522429e-08
  1.32000508e-08 3.46867135e-09 1.06926479e-09 4.34941194e-09
  1.76758874e-08 3.12729820e-09 2.93912628e-08 7.30659888e-09]
 [1.94206091e-08 1.55784630e-08 1.10613261e-08 3.82966991e-09
  5.03393416e-08 2.82992829e-08 4.57844429e-09 4.47462156e-09
  1.55366919e-08 2.57872035e-09 7.15599802e-09 1.34675062e-08
  2.71023701e-08 1.62758145e-08 1.17587415e-08 1.09268745e-08
  3.35502359e-09 3.34574501e-08 1.41335947e-08 8.44941805e-09]
 [5.92188298e-09 2.54038675e-08 1.15597789e-08 5.02641573e-09
  1.15465220e-08 4.92092544e-09 1.52577115e-08 3.67030388e-08
  8.40225667e-09 2.73523248e-08 1.06278470e-08 8.71607586e-09
  1.85189575e-09 3.57539909e-09 1.75874226e-09 4.25893916e-08
  1.03105284e-08 1.49752584e-08 9.68315028e-09 7.21569560e-09]]
```
