# Supplementary material for: Optimization of Microchannels and Application of Basic Activation Functions of Deep Neural Network for Accuracy Analysis of Microfluidic Parameter Data
Source: Micromachines (Basel). 2022 Aug 20;13(8):1352. doi: 10.3390/mi13081352 (PMC9413860; doi:10.3390/mi13081352)
Supplement: Supplementary file 1 [file micromachines-13-01352-s001.zip › Model1_ReLU_adam_epoch_20_batch_50__1650269467669.pdf]

Activation functions: ReLU
Optimizer: adam
Epochs = 20, Batch size = 50
Threshold value = 6.000000000000001e-05
Number of folds = 5
Accuracy of each fold : [91.875, 94.0625, 96.25, 88.75, 87.46081504702194]
Avg accuracy :   91.68 %
Epoch loss :

```
[[9.40557641e-07 2.99303338e-08 2.83017165e-09 8.47299730e-10
  6.90350277e-10 6.84792778e-10 6.74832468e-10 6.85703272e-10
  6.92065016e-10 7.23352822e-10 7.20205284e-10 6.81237011e-10
  7.19849014e-10 7.08608283e-10 7.92214350e-10 7.51328832e-10
  6.99026559e-10 6.86605495e-10 6.79861223e-10 6.88609558e-10]
 [6.89962198e-10 6.95376090e-10 7.13714643e-10 7.59401875e-10
  7.57100549e-10 7.07492120e-10 7.67606423e-10 9.28104149e-10
  7.68132169e-10 7.45403517e-10 7.27376548e-10 7.89869947e-10
  7.55289220e-10 7.38461570e-10 7.59235896e-10 7.66906760e-10
  7.06849412e-10 7.06396330e-10 7.09228454e-10 8.33959957e-10]
 [7.82506671e-10 7.16785242e-10 6.91946000e-10 7.48316631e-10
  7.86433807e-10 7.80833176e-10 8.84394558e-10 7.53184792e-10
  7.02736480e-10 8.04278200e-10 7.83583309e-10 7.59104168e-10
  7.42389428e-10 7.64483032e-10 8.22789170e-10 8.45057468e-10
  8.12322209e-10 7.76900211e-10 7.08961279e-10 7.98841493e-10]
 [8.62990401e-10 7.10338510e-10 7.20950077e-10 6.96297575e-10
  7.05801140e-10 7.67547026e-10 7.81590792e-10 7.17257531e-10
  7.23884175e-10 8.42943437e-10 7.28996974e-10 8.27179991e-10
  8.67460992e-10 7.49646900e-10 9.82633863e-10 8.74901485e-10
  9.79420323e-10 1.10255127e-09 9.27065591e-10 7.13076709e-10]
 [7.74822317e-10 8.76247352e-10 7.78938081e-10 1.00282871e-09
  1.43158929e-09 1.34119615e-09 8.12203971e-10 8.15774670e-10
  9.76417391e-10 9.85203252e-10 8.80700124e-10 8.46024750e-10
  9.90406535e-10 7.93901833e-10 8.65600813e-10 1.13488441e-09
  9.38607414e-10 1.16282728e-09 1.26502009e-09 7.23364979e-10]]
```
