# Supplementary material for: Optimization of Microchannels and Application of Basic Activation Functions of Deep Neural Network for Accuracy Analysis of Microfluidic Parameter Data
Source: Micromachines (Basel). 2022 Aug 20;13(8):1352. doi: 10.3390/mi13081352 (PMC9413860; doi:10.3390/mi13081352)
Supplement: Supplementary file 1 [file micromachines-13-01352-s001.zip › Model_1_LeakyReLU_adam_epoch_20_batch_50__threshold 5e-5.pdf]

Activation functions: LeakyReLU
Optimizer: adam
Epochs = 20, Batch size = 50
Threshold value = 5e-05
Number of folds = 5
Accuracy of each fold : [93.125, 94.6875, 95.625, 92.1875, 97.17868338557993]
Avg accuracy :   94.56 %
Epoch loss :

```
[[4.66227812e-06 7.30785814e-08 6.65705624e-09 1.09688381e-09
  6.08723016e-10 5.80673398e-10 6.21950658e-10 5.77132009e-10
  5.63059654e-10 5.78495807e-10 6.13040119e-10 5.88513627e-10
  5.56172830e-10 5.42055345e-10 5.67753344e-10 5.48141421e-10
  5.22969168e-10 5.19597976e-10 5.19048748e-10 5.05231523e-10]
 [5.11282239e-10 5.18123322e-10 5.26317323e-10 5.03901698e-10
  5.12399845e-10 4.97132169e-10 5.07051956e-10 5.13876219e-10
  5.13600662e-10 4.90393448e-10 5.13178833e-10 4.97599961e-10
  4.77102302e-10 5.85257454e-10 4.83251050e-10 4.96789720e-10
  5.23013521e-10 4.63033861e-10 4.55722848e-10 4.54879440e-10]
 [4.52544197e-10 4.71233996e-10 5.24294164e-10 4.86378882e-10
  4.40337516e-10 4.36703312e-10 4.42706399e-10 5.79556625e-10
  4.54621923e-10 4.20258051e-10 4.25502938e-10 4.25192631e-10
  4.25334185e-10 4.01947947e-10 5.15118892e-10 4.83410811e-10
  3.95767252e-10 3.77716913e-10 3.76618986e-10 3.97976596e-10]
 [3.79875381e-10 3.49593687e-10 3.56187330e-10 3.74430487e-10
  4.26647856e-10 4.08911238e-10 3.47507717e-10 4.62212185e-10
  3.95508154e-10 3.66423697e-10 4.09857814e-10 3.25575317e-10
  3.20053539e-10 3.40191958e-10 3.07921827e-10 3.52647134e-10
  3.66163877e-10 6.23939123e-10 4.05092820e-10 3.36305039e-10]
 [3.33443995e-10 3.16270149e-10 3.32277428e-10 4.01970901e-10
  3.56291968e-10 4.38678788e-10 4.11057494e-10 3.65442537e-10
  3.33432643e-10 3.75154879e-10 2.69446687e-10 3.23334803e-10
  3.37245204e-10 3.01536740e-10 3.59539010e-10 2.89574503e-10
  3.77198661e-10 3.16455362e-10 2.93678776e-10 3.92304633e-10]]
```
