# Supplementary material for: Optimization of Microchannels and Application of Basic Activation Functions of Deep Neural Network for Accuracy Analysis of Microfluidic Parameter Data
Source: Micromachines (Basel). 2022 Aug 20;13(8):1352. doi: 10.3390/mi13081352 (PMC9413860; doi:10.3390/mi13081352)
Supplement: Supplementary file 1 [file micromachines-13-01352-s001.zip › Model_1_swish_adam_epoch_20_batch_50__1651856896173.pdf]

Activation functions: swish
Optimizer: adam
Epochs = 20, Batch size = 50
Threshold value = 6.000000000000001e-05
Number of folds = 5
Accuracy of each fold : [88.125, 80.9375, 93.4375, 88.4375, 83.07210031347962]
Avg accuracy :   86.80 %
Epoch loss :
[[1.54629242e-06 1.03695697e-07 7.59398944e-09 1.01555897e-09
  5.49584822e-10 5.64212566e-10 5.67265124e-10 5.79799930e-10
  6.93070323e-10 6.28420094e-10 5.65407055e-10 6.44953146e-10
  6.04076289e-10 5.79525428e-10 5.99990058e-10 5.60245184e-10
  5.57168478e-10 5.63588676e-10 5.90861970e-10 6.64371391e-10]
 [6.18952833e-10 6.09484851e-10 5.50978485e-10 5.78815940e-10
  6.13788020e-10 7.87210130e-10 7.14298731e-10 6.14603424e-10
  9.71635217e-10 7.70943975e-10 9.13705389e-10 1.15231835e-09
  8.32425406e-10 3.18140603e-09 1.82981736e-08 5.18518606e-09
  2.40173126e-08 2.68704081e-09 4.52029170e-08 1.19855033e-08]
 [1.14820342e-09 2.57360466e-09 1.52527893e-08 1.13532321e-08
  1.67215237e-08 1.71264212e-08 6.41220854e-09 2.83517365e-09
  7.43741140e-08 1.43234837e-08 1.93826510e-09 8.82726281e-10
  7.46103346e-10 2.06565653e-09 4.01900291e-09 4.55243976e-09
  8.67467804e-08 5.16623322e-09 1.70092040e-09 6.06386996e-10]
 [1.31322364e-09 1.61706859e-09 2.28351849e-09 1.17766561e-08
  2.75569096e-08 7.78371501e-09 1.04331233e-09 3.64583541e-09
  8.07444707e-08 6.92431668e-09 8.43240089e-10 1.58750901e-09
  4.95886532e-09 7.73576225e-09 1.62093694e-09 5.43295009e-09
  4.48601618e-08 1.00352748e-08 8.53709281e-09 1.32326594e-09]
 [1.03235109e-09 5.34443290e-09 9.17899285e-08 1.38224179e-08
  2.40060727e-09 7.89533217e-10 2.56315680e-09 3.90035026e-09
  4.42772219e-09 3.60018371e-09 1.21891777e-08 4.25354907e-09
  1.40803440e-08 2.89526518e-08 5.49897683e-09 3.13126636e-09
  4.10515248e-08 7.87568144e-09 5.78847459e-09 3.02649772e-09]]
