# Supplementary material for: Optimization of Microchannels and Application of Basic Activation Functions of Deep Neural Network for Accuracy Analysis of Microfluidic Parameter Data
Source: Micromachines (Basel). 2022 Aug 20;13(8):1352. doi: 10.3390/mi13081352 (PMC9413860; doi:10.3390/mi13081352)
Supplement: Supplementary file 1 [file micromachines-13-01352-s001.zip › second data file Processed Data of Microfluidic parameters.pdf]

C1	Cout	v (Inlets)	Pressure at Inlet1	pressure at Inlet2	v(outlet)	pressure drop
0.00000001667	0.000000031673	0.000003110074627	0	0	0.00000469925816	0
0.00000003334	0.000000063346	0.000006220149254	0	0	0.00000939851632	0
0.00000005001	0.000000095019	0.000009330223881	0	0	0.00001409777448	0
0.00000006668	0.000000130026	0.00001244029851	0	0	0.00001929169139	0
0.00000008335	0.000000163366	0.00001555037313	0	0	0.00002423827893	0
0.00000010002	0.0000001667	0.00001866044776	0	0	0.00002473293769	0
0.00000011669	0.00000021671	0.00002177052239	0	0	0.00003215281899	0
0.00000013336	0.00000025005	0.00002488059701	0	0	0.00003709940653	0
0.00000015003	0.00000026672	0.00002799067164	0	0	0.0000395727003	0
0.0000001667	0.00000031673	0.00003110074627	0	0	0.0000469925816	0
0.00000018337	0.00000035007	0.0000342108209	0	0	0.00005193916914	0
0.00000020004	0.00000036674	0.00003732089552	0	0	0.00005441246291	0
0.00000021671	0.00000040008	0.00004043097015	0	0	0.00005935905045	0
0.00000023338	0.00000045009	0.00004354104478	0	0	0.00006677893175	0
0.00000025005	0.00000046676	0.0000466511194	0	0	0.00006925222552	0
0.00000026672	0.00000051677	0.00004976119403	0	0	0.00007667210682	0
0.00000028339	0.00000055011	0.00005287126866	0	0	0.00008161869436	0
0.00000030006	0.00000058345	0.00005598134328	0	0	0.0000865652819	0
0.00000031673	0.00000061679	0.00005909141791	0	0	0.00009151186944	0
0.0000003334	0.00000063346	0.00006220149254	0	0	0.0000939851632	0
0.00000035007	0.0000006668	0.00006531156716	0	0	0.00009893175074	0
0.00000036674	0.00000070014	0.00006842164179	0	0	0.0001038783383	0
0.00000038341	0.00000075015	0.00007153171642	0	0	0.0001112982196	0
0.00000040008	0.00000076682	0.00007464179104	0	0	0.0001137715134	0
0.00000041675	0.00000081683	0.00007775186567	0	0	0.0001211913947	0
0.00000043342	0.00000085017	0.0000808619403	0	0	0.0001261379822	0
0.00000045009	0.00000086684	0.00008397201493	0	0	0.000128611276	0
0.00000046676	0.00000091685	0.00008708208955	0	0	0.0001360311573	0
0.00000048343	0.00000093352	0.00009019216418	0	0	0.000138504451	0
0.0000005001	0.00000095019	0.00009330223881	0	0	0.0001409777448	0
0.00000051677	0.0000010002	0.00009641231343	0	0	0.0001483976261	0
0.00000053344	0.00000105021	0.00009952238806	0	0	0.0001558175074	0
0.00000055011	0.00000106688	0.0001026324627	0	0	0.0001582908012	0
0.00000056678	0.00000110022	0.0001057425373	0	0	0.0001632373887	0
0.00000058345	0.00000111689	0.0001088526119	0	0	0.0001657106825	0
0.00000060012	0.0000011669	0.0001119626866	0	0	0.0001731305638	0
0.00000061679	0.00000118357	0.0001150727612	0	0	0.0001756038576	0
0.00000063346	0.00000125025	0.0001181828358	0	0	0.0001854970326	0
0.00000065013	0.00000128359	0.0001212929104	0	0	0.0001904436202	0
0.0000006668	0.00000130026	0.0001244029851	0	0	0.0001929169139	0

0.00000068347	0.0000013336	0.0001275130597	0	0	0.0001978635015	0
0.00000070014	0.00000138361	0.0001306231343	0	0	0.0002052833828	0
0.00000071681	0.00000140028	0.000133733209	0	0	0.0002077566766	0
0.00000073348	0.00000143362	0.0001368432836	0	0	0.0002127032641	0
0.00000075015	0.00000146696	0.0001399533582	0	0	0.0002176498516	0
0.00000076682	0.00000151697	0.0001430634328	0	0	0.0002250697329	0
0.00000078349	0.00000153364	0.0001461735075	0	0	0.0002275430267	0
0.00000080016	0.00000155031	0.0001492835821	0	0	0.0002300163205	0
0.00000081683	0.00000160032	0.0001523936567	0	0	0.0002374362018	0
0.0000008335	0.00000163366	0.0001555037313	0	0	0.0002423827893	0
0.00000085017	0.00000165033	0.000158613806	0	0	0.0002448560831	0
0.00000086684	0.00000168367	0.0001617238806	0	0	0.0002498026706	0
0.00000088351	0.00000173368	0.0001648339552	0	0	0.0002572225519	0
0.00000090018	0.00000176702	0.0001679440299	0	0	0.0002621691395	0
0.00000091685	0.000001667	0.0001710541045	0	0	0.0002473293769	0
0.00000093352	0.00000186704	0.0001741641791	0	0	0.0002770089021	0
0.00000095019	0.00000188371	0.0001772742537	0	0	0.0002794821958	0
0.00000096686	0.00000191705	0.0001803843284	0	0	0.0002844287834	0
0.00000098353	0.00000195039	0.000183494403	0	0	0.0002893753709	0
0.0000010002	0.00000198373	0.0001866044776	0	0	0.0002943219585	0
0.00000101687	0.0000020004	0.0001897145522	0	0	0.0002967952522	0
0.00000103354	0.00000203374	0.0001928246269	0	0	0.0003017418398	0
0.00000105021	0.00000205041	0.0001959347015	0	0	0.0003042151335	0
0.00000106688	0.00000208375	0.0001990447761	0	0	0.0003091617211	0
0.00000108355	0.00000213376	0.0002021548507	0	0	0.0003165816024	0
0.00000110022	0.0000021671	0.0002052649254	0	0	0.0003215281899	0
0.00000111689	0.00000220044	0.000208375	0	0	0.0003264747774	0
0.00000113356	0.00000225045	0.0002114850746	0	0	0.0003338946588	0
0.00000115023	0.00000226712	0.0002145951493	0	0	0.0003363679525	0
0.0000011669	0.00000230046	0.0002177052239	0	0	0.0003413145401	0
0.00000118357	0.0000023338	0.0002208152985	0	0	0.0003462611276	0
0.00000120024	0.00000236714	0.0002239253731	0	0	0.0003512077151	0
0.00000121691	0.00000241715	0.0002270354478	0	0	0.0003586275964	0
0.00000123358	0.00000243382	0.0002301455224	0	0	0.0003611008902	0
0.00000125025	0.00000248383	0.000233255597	0	0	0.0003685207715	0
0.00000126692	0.0000025005	0.0002363656716	0	0	0.0003709940653	0
0.00000128359	0.00000253384	0.0002394757463	0	0	0.0003759406528	0
0.00000130026	0.00000258385	0.0002425858209	0	0	0.0003833605341	0
0.00000131693	0.00000261719	0.0002456958955	0	0	0.0003883071217	0
0.0000013336	0.00000265053	0.0002488059701	0	0	0.0003932537092	0

0.00000135027	0.0000026672	0.0002519160448	0	0	0.000395727003	0
0.00000136694	0.00000270054	0.0002550261194	0	0	0.0004006735905	0
0.00000138361	0.00000273388	0.000258136194	0	0	0.000405620178	0
0.00000140028	0.00000278389	0.0002612462687	0	0	0.0004130400593	0
0.00000141695	0.00000281723	0.0002643563433	0	0	0.0004179866469	0
0.00000143362	0.0000028339	0.0002674664179	0	0	0.0004204599407	0
0.00000145029	0.00000286724	0.0002705764925	0	0	0.0004254065282	0
0.00000146696	0.00000291725	0.0002736865672	0	0	0.0004328264095	0
0.00000148363	0.00000295059	0.0002767966418	0	0	0.000437772997	0
0.0000015003	0.00000298393	0.0002799067164	0	0	0.0004427195846	0
0.00000151697	0.0000030006	0.000283016791	0	0	0.0004451928783	0
0.00000153364	0.00000303394	0.0002861268657	0	0	0.0004501394659	0
0.00000155031	0.00000308395	0.0002892369403	0	0	0.0004575593472	0
0.00000156698	0.00000311729	0.0002923470149	0	0	0.0004625059347	0
0.00000158365	0.00000315063	0.0002954570896	0	0	0.0004674525223	0
0.00000160032	0.0000031673	0.0002985671642	0	0	0.000469925816	0
0.00000161699	0.00000320064	0.0003016772388	0	0	0.0004748724036	0
0.00000163366	0.00000323398	0.0003047873134	0	0	0.0004798189911	0
0.00000165033	0.00000326732	0.0003078973881	0	0	0.0004847655786	0
0.000001667	0.00000330066	0.0003110074627	0	0	0.0004897121662	0
0.00000168367	0.000003334	0.0003141175373	0	0	0.0004946587537	0
0.00000170034	0.00000336734	0.0003172276119	0	0	0.0004996053412	0
0.00000171701	0.00000338401	0.0003203376866	0	0	0.000502078635	0
0.00000173368	0.00000343402	0.0003234477612	0	0	0.0005094985163	0
0.00000175035	0.00000348403	0.0003265578358	0	0	0.0005169183976	0
0.00000176702	0.0000035007	0.0003296679104	0	0	0.0005193916914	0
0.00000178369	0.00000355071	0.0003327779851	0	0	0.0005268115727	0
0.00000180036	0.00000356738	0.0003358880597	0	0	0.0005292848665	0
0.00000181703	0.00000358405	0.0003389981343	0	0	0.0005317581602	0
0.0000018337	0.00000361739	0.000342108209	0	0	0.0005367047478	0
0.00000185037	0.0000036674	0.0003452182836	0	0	0.0005441246291	0
0.00000186704	0.00000370074	0.0003483283582	0	0	0.0005490712166	0
0.00000188371	0.00000371741	0.0003514384328	0	0	0.0005515445104	0
0.00000190038	0.00000375075	0.0003545485075	0	0	0.0005564910979	0
0.00000191705	0.00000378409	0.0003576585821	0	0	0.0005614376855	0
0.00000193372	0.00000381743	0.0003607686567	0	0	0.000566384273	0
0.00000195039	0.0000038341	0.0003638787313	0	0	0.0005688575668	0
0.00000196706	0.00000386744	0.000366988806	0	0	0.0005738041543	0
0.00000198373	0.00000390078	0.0003700988806	0	0	0.0005787507418	0
0.0000020004	0.00000395079	0.0003732089552	0	0	0.0005861706231	0

0.00000201707	0.00000398413	0.0003763190299	0	0	0.0005911172107	0
0.00000203374	0.0000040008	0.0003794291045	0	0	0.0005935905045	0
0.00000205041	0.00000403414	0.0003825391791	0	0	0.000598537092	0
0.00000206708	0.00000406748	0.0003856492537	0	0	0.0006034836795	0
0.00000208375	0.00000411749	0.0003887593284	0	0	0.0006109035608	0
0.00000210042	0.00000413416	0.000391869403	0	0	0.0006133768546	0
0.00000211709	0.0000041675	0.0003949794776	0	0	0.0006183234421	0
0.00000213376	0.00000420084	0.0003980895522	0	0	0.0006232700297	0
0.00000215043	0.00000421751	0.0004011996269	0	0	0.0006257433234	0
0.0000021671	0.00000425085	0.0004043097015	0	0	0.000630689911	0
0.00000218377	0.00000428419	0.0004074197761	0	0	0.0006356364985	0
0.00000220044	0.00000431753	0.0004105298507	0	0	0.0006405830861	0
0.00000221711	0.00000435087	0.0004136399254	0	0	0.0006455296736	0
0.00000223378	0.00000440088	0.00041675	0	0	0.0006529495549	0
0.00000225045	0.00000443422	0.0004198600746	0	0	0.0006578961424	0
0.00000226712	0.00000446756	0.0004229701493	0	0	0.00066284273	0
0.00000228379	0.0000045009	0.0004260802239	0	0	0.0006677893175	0
0.00000230046	0.00000453424	0.0004291902985	0	0	0.000672735905	0
0.00000231713	0.00000456758	0.0004323003731	0	0	0.0006776824926	0
0.0000023338	0.00000461759	0.0004354104478	0	0	0.0006851023739	0
0.00000235047	0.00000465093	0.0004385205224	0	0	0.0006900489614	0
0.00000236714	0.00000468427	0.000441630597	0	0	0.000694995549	0
0.00000238381	0.00000471761	0.0004447406716	0	0	0.0006999421365	0
0.00000240048	0.00000475095	0.0004478507463	0	0	0.000704888724	0
0.00000241715	0.00000480096	0.0004509608209	0	0	0.0007123086053	0
0.00000243382	0.0000048343	0.0004540708955	0	0	0.0007172551929	0
0.00000245049	0.00000485097	0.0004571809701	0	0	0.0007197284866	0
0.00000246716	0.00000488431	0.0004602910448	0	0	0.0007246750742	0
0.00000248383	0.00000493432	0.0004634011194	0	0	0.0007320949555	0
0.0000025005	0.00000495099	0.000466511194	0	0	0.0007345682493	0
0.00000251717	0.00000496766	0.0004696212687	0	0	0.000737041543	0
0.00000253384	0.000005001	0.0004727313433	0	0	0.0007419881306	0
0.00000255051	0.00000505101	0.0004758414179	0	0	0.0007494080119	0
0.00000256718	0.00000508435	0.0004789514925	0	0	0.0007543545994	0
0.00000258385	0.00000513436	0.0004820615672	0	0	0.0007617744807	0
0.00000260052	0.00000515103	0.0004851716418	0	0	0.0007642477745	0
0.00000261719	0.0000051677	0.0004882817164	0	0	0.0007667210682	0
0.00000263386	0.00000523438	0.000491391791	0	0	0.0007766142433	0
0.00000265053	0.00000525105	0.0004945018657	0	0	0.0007790875371	0
0.0000026672	0.00000530106	0.0004976119403	0	0	0.0007865074184	0

0.00000268387	0.00000531773	0.0005007220149	0	0	0.0007889807122	0
0.00000270054	0.00000535107	0.0005038320896	0	0	0.0007939272997	0
0.00000271721	0.00000536774	0.0005069421642	0	0	0.0007964005935	0
0.00000273388	0.00000540108	0.0005100522388	0	0	0.000801347181	0
0.00000275055	0.00000545109	0.0005131623134	0	0	0.0008087670623	0
0.00000276722	0.00000546776	0.0005162723881	0	0	0.0008112403561	0
0.00000278389	0.00000548443	0.0005193824627	0	0	0.0008137136499	0
0.00000280056	0.00000551777	0.0005224925373	0	0	0.0008186602374	0
0.00000281723	0.00000555111	0.0005256026119	0	0	0.0008236068249	0
0.0000028339	0.00000560112	0.0005287126866	0	0	0.0008310267062	0
0.00000285057	0.00000561779	0.0005318227612	0	0	0.0008335	0
0.00000286724	0.00000565113	0.0005349328358	0	0	0.0008384465875	0
0.00000288391	0.00000568447	0.0005380429104	0	0	0.0008433931751	0
0.00000290058	0.00000571781	0.0005411529851	0	0	0.0008483397626	0
0.00000291725	0.00000575115	0.0005442630597	0	0	0.0008532863501	0
0.00000293392	0.00000578449	0.0005473731343	0	0	0.0008582329377	0
0.00000295059	0.00000581783	0.000550483209	0	0	0.0008631795252	0
0.00000296726	0.00000585117	0.0005535932836	0	0	0.0008681261128	0
0.00000298393	0.00000590118	0.0005567033582	0	0	0.0008755459941	0
0.0000030006	0.00000593452	0.0005598134328	0	0	0.0008804925816	0
0.00000301727	0.00000596786	0.0005629235075	0	0	0.0008854391691	0
0.00000303394	0.00000598453	0.0005660335821	0	0	0.0008879124629	0
0.00000305061	0.0000060012	0.0005691436567	0	0	0.0008903857567	0
0.00000306728	0.00000605121	0.0005722537313	0	0	0.000897805638	0
0.00000308395	0.00000610122	0.000575363806	0	0	0.0009052255193	0
0.00000310062	0.00000613456	0.0005784738806	0	0	0.0009101721068	0
0.00000311729	0.0000061679	0.0005815839552	0	0	0.0009151186944	0
0.00000313396	0.00000620124	0.0005846940299	0	0	0.0009200652819	0
0.00000315063	0.00000623458	0.0005878041045	0	0	0.0009250118694	0
0.0000031673	0.00000628459	0.0005909141791	0	0	0.0009324317507	0
0.00000318397	0.00000630126	0.0005940242537	0	0	0.0009349050445	0
0.00000320064	0.0000063346	0.0005971343284	0	0	0.000939851632	0
0.00000321731	0.00000635127	0.000600244403	0	0	0.0009423249258	0
0.00000323398	0.00000640128	0.0006033544776	0	0	0.0009497448071	0
0.00000325065	0.00000641795	0.0006064645522	0	0	0.0009522181009	0
0.00000326732	0.00000646796	0.0006095746269	0	0	0.0009596379822	0
0.00000328399	0.0000065013	0.0006126847015	0	0	0.0009645845697	0
0.00000330066	0.00000653464	0.0006157947761	0	0	0.0009695311573	0
0.00000331733	0.00000658465	0.0006189048507	0	0	0.0009769510386	0
0.000003334	0.00000661799	0.0006220149254	0	0	0.0009818976261	0

0.00000335067	0.00000663466	0.000625125	0	0	0.0009843709199	0
0.00000336734	0.00000668467	0.0006282350746	0	0	0.0009917908012	0
0.00000338401	0.00000671801	0.0006313451493	0	0	0.0009967373887	0
0.00000340068	0.00000675135	0.0006344552239	0	0	0.001001683976	0
0.00000341735	0.00000678469	0.0006375652985	0	0	0.001006630564	0
0.00000343402	0.00000681803	0.0006406753731	0	0	0.001011577151	0
0.00000345069	0.00000685137	0.0006437854478	0	0	0.001016523739	0
0.00000346736	0.00000686804	0.0006468955224	0	0	0.001018997033	0
0.00000348403	0.00000690138	0.000650005597	0	0	0.00102394362	0
0.0000035007	0.00000693472	0.0006531156716	0	0	0.001028890208	0
0.00000351737	0.00000696806	0.0006562257463	0	0	0.001033836795	0
0.00000353404	0.00000698473	0.0006593358209	0	0	0.001036310089	0
0.00000355071	0.0000070014	0.0006624458955	0	0	0.001038783383	0
0.00000356738	0.00000703474	0.0006655559701	0	0	0.00104372997	0
0.00000358405	0.00000708475	0.0006686660448	0	0	0.001051149852	0
0.00000360072	0.00000713476	0.0006717761194	0	0	0.001058569733	0
0.00000361739	0.0000071681	0.000674886194	0	0	0.00106351632	0
0.00000363406	0.00000720144	0.0006779962687	0	0	0.001068462908	0
0.00000365073	0.00000723478	0.0006811063433	0	0	0.001073409496	0
0.0000036674	0.00000728479	0.0006842164179	0	0	0.001080829377	0
0.00000368407	0.00000730146	0.0006873264925	0	0	0.001083302671	0
0.00000370074	0.0000073348	0.0006904365672	0	0	0.001088249258	0
0.00000371741	0.00000738481	0.0006935466418	0	0	0.001095669139	0
0.00000373408	0.00000741815	0.0006966567164	0	0	0.001100615727	0
0.00000375075	0.00000745149	0.000699766791	0	0	0.001105562315	0
0.00000376742	0.00000748483	0.0007028768657	0	0	0.001110508902	0
0.00000378409	0.00000751817	0.0007059869403	0	0	0.00111545549	0
0.00000380076	0.00000755151	0.0007090970149	0	0	0.001120402077	0
0.00000381743	0.00000760152	0.0007122070896	0	0	0.001127821958	0
0.0000038341	0.00000763486	0.0007153171642	0	0	0.001132768546	0
0.00000385077	0.0000076682	0.0007184272388	0	0	0.001137715134	0
0.00000386744	0.00000770154	0.0007215373134	0	0	0.001142661721	0
0.00000388411	0.00000771821	0.0007246473881	0	0	0.001145135015	0
0.00000390078	0.00000776822	0.0007277574627	0	0	0.001152554896	0
0.00000391745	0.00000780156	0.0007308675373	0	0	0.001157501484	0
0.00000393412	0.00000785157	0.0007339776119	0	0	0.001164921365	0
0.00000395079	0.00000788491	0.0007370876866	0	0	0.001169867953	0
0.00000396746	0.00000791825	0.0007401977612	0	0	0.00117481454	0
0.00000398413	0.00000793492	0.0007433078358	0	0	0.001177287834	0
0.0000040008	0.00000796826	0.0007464179104	0	0	0.001182234421	0

0.00000401747	0.0000080016	0.0007495279851	0	0	0.001187181009	0
0.00000403414	0.00000805161	0.0007526380597	0	0	0.00119460089	0
0.00000405081	0.00000806828	0.0007557481343	0	0	0.001197074184	0
0.00000406748	0.00000808495	0.000758858209	0	0	0.001199547478	0
0.00000408415	0.00000811829	0.0007619682836	0	0	0.001204494065	0
0.00000410082	0.00000815163	0.0007650783582	0	0	0.001209440653	0
0.00000411749	0.00000820164	0.0007681884328	0	0	0.001216860534	0
0.00000413416	0.00000823498	0.0007712985075	0	0	0.001221807122	0
0.00000415083	0.00000825165	0.0007744085821	0	0	0.001224280415	0
0.0000041675	0.00000828499	0.0007775186567	0	0	0.001229227003	0
0.00000418417	0.00000831833	0.0007806287313	0	0	0.001234173591	0
0.00000420084	0.00000835167	0.000783738806	0	0	0.001239120178	0
0.00000421751	0.00000838501	0.0007868488806	0	0	0.001244066766	0
0.00000423418	0.00000841835	0.0007899589552	0	0	0.001249013353	0
0.00000425085	0.00000846836	0.0007930690299	0	0	0.001256433234	0
0.00000426752	0.000008335	0.0007961791045	0	0	0.001236646884	0
0.00000428419	0.00000853504	0.0007992891791	0	0	0.001266326409	0
0.00000430086	0.00000856838	0.0008023992537	0	0	0.001271272997	0
0.00000431753	0.00000861839	0.0008055093284	0	0	0.001278692878	0
0.0000043342	0.00000865173	0.000808619403	0	0	0.001283639466	0
0.00000435087	0.0000086684	0.0008117294776	0	0	0.00128611276	0
0.00000436754	0.00000870174	0.0008148395522	0	0	0.001291059347	0
0.00000438421	0.00000871841	0.0008179496269	0	0	0.001293532641	0
0.00000440088	0.00000875175	0.0008210597015	0	0	0.001298479228	0
0.00000441755	0.00000878509	0.0008241697761	0	0	0.001303425816	0
0.00000443422	0.00000881843	0.0008272798507	0	0	0.001308372404	0
0.00000445089	0.00000885177	0.0008303899254	0	0	0.001313318991	0
0.00000446756	0.00000890178	0.0008335	0	0	0.001320738872	0
0.00000448423	0.00000893512	0.0008366100746	0	0	0.00132568546	0
0.0000045009	0.00000896846	0.0008397201493	0	0	0.001330632047	0
0.00000451757	0.0000090018	0.0008428302239	0	0	0.001335578635	0
0.00000453424	0.00000905181	0.0008459402985	0	0	0.001342998516	0
0.00000455091	0.00000906848	0.0008490503731	0	0	0.00134547181	0
0.00000456758	0.00000910182	0.0008521604478	0	0	0.001350418398	0
0.00000458425	0.00000913516	0.0008552705224	0	0	0.001355364985	0
0.00000460092	0.0000091685	0.000858380597	0	0	0.001360311573	0
0.00000461759	0.00000920184	0.0008614906716	0	0	0.00136525816	0
0.00000463426	0.00000923518	0.0008646007463	0	0	0.001370204748	0
0.00000465093	0.00000926852	0.0008677108209	0	0	0.001375151335	0
0.0000046676	0.00000930186	0.0008708208955	0	0	0.001380097923	0

0.00000468427	0.0000093352	0.0008739309701	0	0	0.00138504451	0
0.00000470094	0.00000936854	0.0008770410448	0	0	0.001389991098	0
0.00000471761	0.00000938521	0.0008801511194	0	0	0.001392464392	0
0.00000473428	0.00000941855	0.000883261194	0	0	0.001397410979	0
0.00000475095	0.00000945189	0.0008863712687	0	0	0.001402357567	0
0.00000476762	0.00000948523	0.0008894813433	0	0	0.001407304154	0
0.00000478429	0.00000951857	0.0008925914179	0	0	0.001412250742	0
0.00000480096	0.00000955191	0.0008957014925	0	0	0.001417197329	0
0.00000481763	0.00000958525	0.0008988115672	0	0	0.001422143917	0
0.0000048343	0.00000961859	0.0009019216418	0	0	0.001427090504	0
0.00000485097	0.00000965193	0.0009050317164	0	0	0.001432037092	0
0.00000486764	0.00000968527	0.000908141791	0	0	0.00143698368	0
0.00000488431	0.00000971861	0.0009112518657	0	0	0.001441930267	0
0.00000490098	0.00000975195	0.0009143619403	0	0	0.001446876855	0
0.00000491765	0.00000978529	0.0009174720149	0	0	0.001451823442	0
0.00000493432	0.00000981863	0.0009205820896	0	0	0.00145677003	0
0.00000495099	0.00000985197	0.0009236921642	0	0	0.001461716617	0
0.00000496766	0.00000990198	0.0009268022388	0	0	0.001469136499	0
0.00000498433	0.00000993532	0.0009299123134	0	0	0.001474083086	0
0.000005001	0.00000996866	0.0009330223881	0	0	0.001479029674	0
0.00000501767	0.000010002	0.0009361324627	0	0	0.001483976261	0
0.00000503434	0.00001003534	0.0009392425373	0	0	0.001488922849	0
0.00000505101	0.00001006868	0.0009423526119	0	0	0.001493869436	0
0.00000506768	0.00001010202	0.0009454626866	0	0	0.001498816024	0
0.00000508435	0.00001013536	0.0009485727612	0	0	0.001503762611	0
0.00000510102	0.00001015203	0.0009516828358	0	0	0.001506235905	0
0.00000511769	0.00001020204	0.0009547929104	0	0	0.001513655786	0
0.00000513436	0.00001025205	0.0009579029851	0	0	0.001521075668	0
0.00000515103	0.00001028539	0.0009610130597	0	0	0.001526022255	0
0.0000051677	0.00001031873	0.0009641231343	0	0	0.001530968843	0
0.00000518437	0.0000103354	0.000967233209	0	0	0.001533442136	0
0.00000520104	0.00001036874	0.0009703432836	0	0	0.001538388724	0
0.00000521771	0.00001040208	0.0009734533582	0	0	0.001543335312	0
0.00000523438	0.00001043542	0.0009765634328	0	0	0.001548281899	0
0.00000525105	0.00001046876	0.0009796735075	0	0	0.001553228487	0
0.00000526772	0.0000105021	0.0009827835821	0	0	0.001558175074	0
0.00000528439	0.00001053544	0.0009858936567	0	0	0.001563121662	0
0.00000530106	0.00001056878	0.0009890037313	0	0	0.001568068249	0
0.00000531773	0.00001060212	0.000992113806	0	0	0.001573014837	0
0.0000053344	0.00001063546	0.0009952238806	0	0	0.001577961424	0

0.00000535107	0.0000106688	0.0009983339552	0	0	0.001582908012	0
0.00000536774	0.00001071881	0.00100144403	0	0	0.001590327893	0
0.00000538441	0.00001073548	0.001004554104	0	0	0.001592801187	0
0.00000540108	0.00001076882	0.001007664179	0	0	0.001597747774	0
0.00000541775	0.00001078549	0.001010774254	0	0	0.001600221068	0
0.00000543442	0.00001081883	0.001013884328	0	0	0.001605167656	0
0.00000545109	0.00001085217	0.001016994403	0	0	0.001610114243	0
0.00000546776	0.00001088551	0.001020104478	0	0	0.001615060831	0
0.00000548443	0.00001091885	0.001023214552	0	0	0.001620007418	0
0.0000055011	0.00001095219	0.001026324627	0	0	0.001624954006	0
0.00000551777	0.00001098553	0.001029434701	0	0	0.001629900593	0
0.00000553444	0.00001101887	0.001032544776	0	0	0.001634847181	0
0.00000555111	0.00001105221	0.001035654851	0	0	0.001639793769	0
0.00000556778	0.00001108555	0.001038764925	0	0	0.001644740356	0
0.00000558445	0.00001113556	0.001041875	0	0	0.001652160237	0
0.00000560112	0.0000111689	0.001044985075	0	0	0.001657106825	0
0.00000561779	0.00001118557	0.001048095149	0	0	0.001659580119	0
0.00000563446	0.00001121891	0.001051205224	0	0	0.001664526706	0
0.00000565113	0.00001126892	0.001054315299	0	0	0.001671946588	0
0.0000056678	0.00001131893	0.001057425373	0	0	0.001679366469	0
0.00000568447	0.0000113356	0.001060535448	0	0	0.001681839763	0
0.00000570114	0.00001136894	0.001063645522	0	0	0.00168678635	0
0.00000571781	0.00001141895	0.001066755597	0	0	0.001694206231	0
0.00000573448	0.00001145229	0.001069865672	0	0	0.001699152819	0
0.00000575115	0.00001148563	0.001072975746	0	0	0.001704099407	0
0.00000576782	0.0000115023	0.001076085821	0	0	0.0017065727	0
0.00000578449	0.00001151897	0.001079195896	0	0	0.001709045994	0
0.00000580116	0.00001155231	0.00108230597	0	0	0.001713992582	0
0.00000581783	0.00001161899	0.001085416045	0	0	0.001723885757	0
0.0000058345	0.00001165233	0.001088526119	0	0	0.001728832344	0
0.00000585117	0.00001168567	0.001091636194	0	0	0.001733778932	0
0.00000586784	0.00001171901	0.001094746269	0	0	0.001738725519	0
0.00000588451	0.00001173568	0.001097856343	0	0	0.001741198813	0
0.00000590118	0.00001176902	0.001100966418	0	0	0.001746145401	0
0.00000591785	0.00001180236	0.001104076493	0	0	0.001751091988	0
0.00000593452	0.0000118357	0.001107186567	0	0	0.001756038576	0
0.00000595119	0.00001186904	0.001110296642	0	0	0.001760985163	0
0.00000596786	0.00001190238	0.001113406716	0	0	0.001765931751	0
0.00000598453	0.00001193572	0.001116516791	0	0	0.001770878338	0
0.0000060012	0.00001196906	0.001119626866	0	0	0.001775824926	0

0.00000601787	0.0000120024	0.00112273694	0	0	0.001780771513	0
0.00000603454	0.00001201907	0.001125847015	0	0	0.001783244807	0
0.00000605121	0.00001205241	0.00112895709	0	0	0.001788191395	0
0.00000606788	0.00001208575	0.001132067164	0	0	0.001793137982	0
0.00000608455	0.00001211909	0.001135177239	0	0	0.00179808457	0.0000000001002248743
0.00000610122	0.00001215243	0.001138287313	0	0.0000000001001820324	0.001803031157	0.0000000001005005961
0.00000611789	0.00001218577	0.001141397388	0	0.0000000001004557538	0.001807977745	0.0000000001007763179
0.00000613456	0.00001223578	0.001144507463	0	0.0000000001007294752	0.001815397626	0.0000000001011899006
0.00000615123	0.00001226912	0.001147617537	0	0.0000000001010031966	0.001820344214	0.0000000001014656224
0.0000061679	0.00001231913	0.001150727612	0	0.000000000101276918	0.001827764095	0.0000000001018792051
0.00000618457	0.0000123358	0.001153837687	0	0.0000000001015506394	0.001830237389	0.000000000102017066
0.00000620124	0.00001236914	0.001156947761	0	0.0000000001018243608	0.001835183976	0.0000000001022927878
0.00000621791	0.00001240248	0.001160057836	0	0.0000000001020980822	0.001840130564	0.0000000001025685096
0.00000623458	0.00001243582	0.00116316791	0	0.0000000001023718036	0.001845077151	0.0000000001028442314
0.00000625125	0.00001246916	0.001166277985	0	0.000000000102645525	0.001850023739	0.0000000001031199532
0.00000626792	0.0000125025	0.00116938806	0	0.0000000001029192464	0.001854970326	0.000000000103395675
0.00000628459	0.00001253584	0.001172498134	0	0.0000000001031929678	0.001859916914	0.0000000001036713968
0.00000630126	0.00001255251	0.001175608209	0	0.0000000001034666892	0.001862390208	0.0000000001038092577
0.00000631793	0.00001258585	0.001178718284	0	0.0000000001037404106	0.001867336795	0.0000000001040849795
0.0000063346	0.00001261919	0.001181828358	0	0.000000000104014132	0.001872283383	0.0000000001043607013
0.00000635127	0.00001265253	0.001184938433	0	0.0000000001042878534	0.00187722997	0.0000000001046364231
0.00000636794	0.00001268587	0.001188048507	0	0.0000000001045615748	0.001882176558	0.0000000001049121449
0.00000638461	0.00001271921	0.001191158582	0	0.0000000001048352962	0.001887123145	0.0000000001051878667
0.00000640128	0.00001275255	0.001194268657	0	0.0000000001051090176	0.001892069733	0.0000000001054635885
0.00000641795	0.00001280256	0.001197378731	0	0.000000000105382739	0.001899489614	0.0000000001058771712
0.00000643462	0.0000128359	0.001200488806	0	0.0000000001056564604	0.001904436202	0.000000000106152893
0.00000645129	0.00001288591	0.001203598881	0	0.0000000001059301818	0.001911856083	0.0000000001065664757
0.00000646796	0.00001290258	0.001206708955	0	0.0000000001062039032	0.001914329377	0.0000000001067043366
0.00000648463	0.00001293592	0.00120981903	0	0.0000000001064776246	0.001919275964	0.0000000001069800584
0.0000065013	0.00001296926	0.001212929104	0	0.000000000106751346	0.001924222552	0.0000000001072557802
0.00000651797	0.00001301927	0.001216039179	0	0.0000000001070250674	0.001931642433	0.0000000001076693629
0.00000653464	0.00001303594	0.001219149254	0	0.0000000001072987888	0.001934115727	0.0000000001078072238
0.00000655131	0.00001306928	0.001222259328	0	0.0000000001075725102	0.001939062315	0.0000000001080829456
0.00000656798	0.00001311929	0.001225369403	0	0.0000000001078462316	0.001946482196	0.0000000001084965283
0.00000658465	0.00001315263	0.001228479478	0	0.000000000108119953	0.001951428783	0.0000000001087722501
0.00000660132	0.00001318597	0.001231589552	0	0.0000000001083936744	0.001956375371	0.0000000001090479719
0.00000661799	0.00001321931	0.001234699627	0	0.0000000001086673958	0.001961321958	0.0000000001093236937
0.00000663466	0.00001323598	0.001237809701	0	0.0000000001089411172	0.001963795252	0.0000000001094615546
0.00000665133	0.00001328599	0.001240919776	0	0.0000000001092148386	0.001971215134	0.0000000001098751373
0.000006668	0.00001331933	0.001244029851	0	0.00000000010948856	0.001976161721	0.0000000001101508591

0.00000668467	0.000013336	0.001247139925	0	0.0000000001097622814	0.001978635015	0.00000000011028872
0.00000670134	0.00001336934	0.00125025	0	0.0000000001100360028	0.001983581602	0.0000000001105644418
0.00000671801	0.00001340268	0.001253360075	0	0.0000000001103097242	0.00198852819	0.0000000001108401636
0.00000673468	0.00001343602	0.001256470149	0	0.0000000001105834456	0.001993474777	0.0000000001111158854
0.00000675135	0.00001346936	0.001259580224	0	0.000000000110857167	0.001998421365	0.0000000001113916072
0.00000676802	0.00001348603	0.001262690299	0	0.0000000001111308884	0.002000894659	0.0000000001115294681
0.00000678469	0.00001351937	0.001265800373	0	0.0000000001114046098	0.002005841246	0.0000000001118051899
0.00000680136	0.00001356938	0.001268910448	0	0.0000000001116783312	0.002013261128	0.0000000001122187726
0.00000681803	0.00001361939	0.001272020522	0	0.0000000001119520526	0.002020681009	0.0000000001126323553
0.0000068347	0.00001365273	0.001275130597	0	0.000000000112225774	0.002025627596	0.0000000001129080771
0.00000685137	0.00001368607	0.001278240672	0	0.0000000001124994954	0.002030574184	0.0000000001131837989
0.00000686804	0.00001370274	0.001281350746	0	0.0000000001127732168	0.002033047478	0.0000000001133216598
0.00000688471	0.00001373608	0.001284460821	0	0.0000000001130469382	0.002037994065	0.0000000001135973816
0.00000690138	0.00001378609	0.001287570896	0	0.0000000001133206596	0.002045413947	0.0000000001140109643
0.00000691805	0.00001380276	0.00129068097	0	0.000000000113594381	0.00204788724	0.0000000001141488252
0.00000693472	0.0000138361	0.001293791045	0	0.0000000001138681024	0.002052833828	0.000000000114424547
0.00000695139	0.00001388611	0.001296901119	0	0.0000000001141418238	0.002060253709	0.0000000001148381297
0.00000696806	0.00001391945	0.001300011194	0	0.0000000001144155452	0.002065200297	0.0000000001151138515
0.00000698473	0.00001395279	0.001303121269	0	0.0000000001146892666	0.002070146884	0.0000000001153895733
0.0000070014	0.00001396946	0.001306231343	0	0.000000000114962988	0.002072620178	0.0000000001155274342
0.00000701807	0.0000140028	0.001309341418	0	0.0000000001152367094	0.002077566766	0.000000000115803156
0.00000703474	0.00001405281	0.001312451493	0	0.0000000001155104308	0.002084986647	0.0000000001162167387
0.00000705141	0.00001408615	0.001315561567	0	0.0000000001157841522	0.002089933234	0.0000000001164924605
0.00000706808	0.00001411949	0.001318671642	0	0.0000000001160578736	0.002094879822	0.0000000001167681823
0.00000708475	0.00001415283	0.001321781716	0	0.000000000116331595	0.002099826409	0.0000000001170439041
0.00000710142	0.0000141695	0.001324891791	0	0.0000000001166053164	0.002102299703	0.000000000117181765
0.00000711809	0.00001420284	0.001328001866	0	0.0000000001168790378	0.002107246291	0.0000000001174574868
0.00000713476	0.00001423618	0.00133111194	0	0.0000000001171527592	0.002112192878	0.0000000001177332086
0.00000715143	0.00001426952	0.001334222015	0	0.0000000001174264806	0.002117139466	0.0000000001180089304
0.0000071681	0.00001431953	0.00133733209	0	0.000000000117700202	0.002124559347	0.0000000001184225131
0.00000718477	0.0000143362	0.001340442164	0	0.0000000001179739234	0.002127032641	0.000000000118560374
0.00000720144	0.00001436954	0.001343552239	0.0000000001001720304	0.0000000001182476448	0.002131979228	0.0000000001188360958
0.00000721811	0.00001440288	0.001346662313	0.0000000001004039101	0.0000000001185213662	0.002136925816	0.0000000001191118176
0.00000723478	0.00001443622	0.001349772388	0.0000000001006357898	0.0000000001187950876	0.002141872404	0.0000000001193875394
0.00000725145	0.00001446956	0.001352882463	0.0000000001008676695	0.000000000119068809	0.002146818991	0.0000000001196632612
0.00000726812	0.00001451957	0.001355992537	0.0000000001010995492	0.0000000001193425304	0.002154238872	0.0000000001200768439
0.00000728479	0.00001455291	0.001359102612	0.0000000001013314289	0.0000000001196162518	0.00215918546	0.0000000001203525657
0.00000730146	0.00001458625	0.001362212687	0.0000000001015633086	0.0000000001198899732	0.002164132047	0.0000000001206282875
0.00000731813	0.00001461959	0.001365322761	0.0000000001017951883	0.0000000001201636946	0.002169078635	0.0000000001209040093
0.0000073348	0.00001465293	0.001368432836	0.000000000102027068	0.000000000120437416	0.002174025223	0.0000000001211797311

0.00000735147	0.00001468627	0.00137154291	0.0000000001022589477	0.0000000001207111374	0.00217897181	0.0000000001214554529
0.00000736814	0.00001470294	0.001374652985	0.0000000001024908274	0.0000000001209848588	0.002181445104	0.0000000001215933138
0.00000738481	0.00001475295	0.00137776306	0.0000000001027227071	0.0000000001212585802	0.002188864985	0.0000000001220068965
0.00000740148	0.00001478629	0.001380873134	0.0000000001029545868	0.0000000001215323016	0.002193811573	0.0000000001222826183
0.00000741815	0.00001481963	0.001383983209	0.0000000001031864665	0.000000000121806023	0.00219875816	0.0000000001225583401
0.00000743482	0.00001486964	0.001387093284	0.0000000001034183462	0.0000000001220797444	0.002206178042	0.0000000001229719228
0.00000745149	0.00001488631	0.001390203358	0.0000000001036502259	0.0000000001223534658	0.002208651335	0.0000000001231097837
0.00000746816	0.00001491965	0.001393313433	0.0000000001038821056	0.0000000001226271872	0.002213597923	0.0000000001233855055
0.00000748483	0.00001495299	0.001396423507	0.0000000001041139853	0.0000000001229009086	0.00221854451	0.0000000001236612273
0.0000075015	0.00001498633	0.001399533582	0.000000000104345865	0.00000000012317463	0.002223491098	0.0000000001239369491
0.00000751817	0.000015003	0.001402643657	0.0000000001045777447	0.0000000001234483514	0.002225964392	0.0000000001240748 1
0.00000753484	0.00001503634	0.001405753731	0.0000000001048096244	0.0000000001237220728	0.002230910979	0.0000000001243505318
0.00000755151	0.00001506968	0.001408863806	0.0000000001050415041	0.0000000001239957942	0.002235857567	0.0000000001246262536
0.00000756818	0.00001510302	0.001411973881	0.0000000001052733838	0.0000000001242695156	0.002240804154	0.0000000001249019754
0.00000758485	0.00001513636	0.001415083955	0.0000000001055052635	0.000000000124543237	0.002245750742	0.0000000001251776972
0.00000760152	0.0000151697	0.00141819403	0.0000000001057371432	0.0000000001248169584	0.002250697329	0.000000000125453419
0.00000761819	0.00001520304	0.001421304104	0.0000000001059690229	0.0000000001250906798	0.002255643917	0.0000000001257291408
0.00000763486	0.00001525305	0.001424414179	0.0000000001062009026	0.0000000001253644012	0.002263063798	0.0000000001261427235
0.00000765153	0.00001528639	0.001427524254	0.0000000001064327823	0.0000000001256381226	0.002268010386	0.0000000001264184453
0.0000076682	0.00001531973	0.001430634328	0.000000000106664662	0.000000000125911844	0.002272956973	0.0000000001266941671
0.00000768487	0.0000153364	0.001433744403	0.0000000001068965417	0.0000000001261855654	0.002275430267	0.000000000126832028
0.00000770154	0.00001536974	0.001436854478	0.0000000001071284214	0.0000000001264592868	0.002280376855	0.0000000001271077498
0.00000771821	0.00001540308	0.001439964552	0.0000000001073603011	0.0000000001267330082	0.002285323442	0.0000000001273834716
0.00000773488	0.00001543642	0.001443074627	0.0000000001075921808	0.0000000001270067296	0.00229027003	0.0000000001276591934
0.00000775155	0.00001545309	0.001446184701	0.0000000001078240605	0.000000000127280451	0.002292743323	0.0000000001277970543
0.00000776822	0.00001548643	0.001449294776	0.0000000001080559402	0.0000000001275541724	0.002297689911	0.0000000001280727761
0.00000778489	0.00001551977	0.001452404851	0.0000000001082878199	0.0000000001278278938	0.002302636499	0.0000000001283484979
0.00000780156	0.00001553644	0.001455514925	0.0000000001085196996	0.0000000001281016152	0.002305109792	0.0000000001284863588
0.00000781823	0.00001556978	0.001458625	0.0000000001087515793	0.0000000001283753366	0.00231005638	0.0000000001287620806
0.0000078349	0.00001561979	0.001461735075	0.000000000108983459	0.000000000128649058	0.002317476261	0.0000000001291756633
0.00000785157	0.00001565313	0.001464845149	0.0000000001092153387	0.0000000001289227794	0.002322422849	0.0000000001294513851
0.00000786824	0.00001570314	0.001467955224	0.0000000001094472184	0.0000000001291965008	0.00232984273	0.0000000001298649678
0.00000788491	0.00001575315	0.001471065299	0.0000000001096790981	0.0000000001294702222	0.002337262611	0.0000000001302785505
0.00000790158	0.00001578649	0.001474175373	0.0000000001099109778	0.0000000001297439436	0.002342209199	0.0000000001305542723
0.00000791825	0.00001580316	0.001477285448	0.0000000001101428575	0.000000000130017665	0.002344682493	0.0000000001306921332
0.00000793492	0.0000158365	0.001480395522	0.0000000001103747372	0.0000000001302913864	0.00234962908	0.000000000130967855
0.00000795159	0.00001588651	0.001483505597	0.0000000001106066169	0.0000000001305651078	0.002357048961	0.0000000001313814377
0.00000796826	0.00001591985	0.001486615672	0.0000000001108384966	0.0000000001308388292	0.002361995549	0.0000000001316571595
0.00000798493	0.00001595319	0.001489725746	0.0000000001110703763	0.0000000001311125506	0.002366942136	0.0000000001319328813
0.0000080016	0.00001596986	0.001492835821	0.000000000111302256	0.000000000131386272	0.00236941543	0.0000000001320707422

0.00000801827	0.00001601987	0.001495945896	0.0000000001115341357	0.0000000001316599934	0.002376835312	0.0000000001324843249
0.00000803494	0.00001603654	0.00149905597	0.0000000001117660154	0.0000000001319337148	0.002379308605	0.0000000001326221858
0.00000805161	0.00001608655	0.001502166045	0.0000000001119978951	0.0000000001322074362	0.002386728487	0.0000000001330357685
0.00000806828	0.00001610322	0.001505276119	0.0000000001122297748	0.0000000001324811576	0.00238920178	0.0000000001331736294
0.00000808495	0.00001611989	0.001508386194	0.0000000001124616545	0.000000000132754879	0.002391675074	0.0000000001333114903
0.00000810162	0.00001615323	0.001511496269	0.0000000001126935342	0.0000000001330286004	0.002396621662	0.0000000001335872121
0.00000811829	0.00001621991	0.001514606343	0.0000000001129254139	0.0000000001333023218	0.002406514837	0.0000000001341386557
0.00000813496	0.00001626992	0.001517716418	0.0000000001131572936	0.0000000001335760432	0.002413934718	0.0000000001345522384
0.00000815163	0.00001628659	0.001520826493	0.0000000001133891733	0.0000000001338497646	0.002416408012	0.0000000001346900993
0.0000081683	0.00001631993	0.001523936567	0.000000000113621053	0.000000000134123486	0.002421354599	0.0000000001349658211
0.00000818497	0.00001635327	0.001527046642	0.0000000001138529327	0.0000000001343972074	0.002426301187	0.0000000001352415429
0.00000820164	0.00001638661	0.001530156716	0.0000000001140848124	0.0000000001346709288	0.002431247774	0.0000000001355172647
0.00000821831	0.00001640328	0.001533266791	0.0000000001143166921	0.0000000001349446502	0.002433721068	0.0000000001356551256
0.00000823498	0.00001643662	0.001536376866	0.0000000001145485718	0.0000000001352183716	0.002438667656	0.0000000001359308474
0.00000825165	0.00001646996	0.00153948694	0.0000000001147804515	0.000000000135492093	0.002443614243	0.0000000001362065692
0.00000826832	0.00001648663	0.001542597015	0.0000000001150123312	0.0000000001357658144	0.002446087537	0.0000000001363444301
0.00000828499	0.00001653664	0.00154570709	0.0000000001152442109	0.0000000001360395358	0.002453507418	0.0000000001367580128
0.00000830166	0.00001658665	0.001548817164	0.0000000001154760906	0.0000000001363132572	0.0024609273	0.0000000001371715955
0.00000831833	0.00001661999	0.001551927239	0.0000000001157079703	0.0000000001365869786	0.002465873887	0.0000000001374473173
0.000008335	0.00001665333	0.001555037313	0.00000000011593985	0.0000000001368607	0.002470820475	0.0000000001377230391
0.00000835167	0.00001668667	0.001558147388	0.0000000001161717297	0.0000000001371344214	0.002475767062	0.0000000001379987609
0.00000836834	0.00001672001	0.001561257463	0.0000000001164036094	0.0000000001374081428	0.00248071365	0.0000000001382744827
0.00000838501	0.00001675335	0.001564367537	0.0000000001166354891	0.0000000001376818642	0.002485660237	0.0000000001385502045
0.00000840168	0.00001677002	0.001567477612	0.0000000001168673688	0.0000000001379555856	0.002488133531	0.0000000001386880654
0.00000841835	0.00001680336	0.001570587687	0.0000000001170992485	0.000000000138229307	0.002493080119	0.0000000001389637872
0.00000843502	0.00001685337	0.001573697761	0.0000000001173311282	0.0000000001385030284	0.0025005	0.0000000001393773699
0.00000845169	0.00001688671	0.001576807836	0.0000000001175630079	0.0000000001387767498	0.002505446588	0.0000000001396530917
0.00000846836	0.00001692005	0.00157991791	0.0000000001177948876	0.0000000001390504712	0.002510393175	0.0000000001399288135
0.00000848503	0.00001695339	0.001583027985	0.0000000001180267673	0.0000000001393241926	0.002515339763	0.0000000001402045353
0.0000085017	0.00001698673	0.00158613806	0.000000000118258647	0.000000000139597914	0.00252028635	0.0000000001404802571
0.00000851837	0.0000170034	0.001589248134	0.0000000001184905267	0.0000000001398716354	0.002522759644	0.000000000140618118
0.00000853504	0.00001703674	0.001592358209	0.0000000001187224064	0.0000000001401453568	0.002527706231	0.0000000001408938398
0.00000855171	0.00001707008	0.001595468284	0.0000000001189542861	0.0000000001404190782	0.002532652819	0.0000000001411695616
0.00000856838	0.00001712009	0.001598578358	0.0000000001191861658	0.0000000001406927996	0.0025400727	0.0000000001415831443
0.00000858505	0.00001715343	0.001601688433	0.0000000001194180455	0.000000000140966521	0.002545019288	0.0000000001418588661
0.00000860172	0.00001718677	0.001604798507	0.0000000001196499252	0.0000000001412402424	0.002549965875	0.0000000001421345879
0.00000861839	0.00001722011	0.001607908582	0.0000000001198818049	0.0000000001415139638	0.002554912463	0.0000000001424103097
0.00000863506	0.00001725345	0.001611018657	0.0000000001201136846	0.0000000001417876852	0.00255985905	0.0000000001426860315
0.00000865173	0.00001728679	0.001614128731	0.0000000001203455643	0.0000000001420614066	0.002564805638	0.0000000001429617533
0.0000086684	0.00001730346	0.001617238806	0.000000000120577444	0.000000000142335128	0.002567278932	0.0000000001430996142

0.00000868507	0.0000173368	0.001620348881	0.0000000001208093237	0.0000000001426088494	0.002572225519	0.000000000143375336
0.00000870174	0.00001737014	0.001623458955	0.0000000001210412034	0.0000000001428825708	0.002577172107	0.0000000001436510578
0.00000871841	0.00001742015	0.00162656903	0.0000000001212730831	0.0000000001431562922	0.002584591988	0.0000000001440646405
0.00000873508	0.00001745349	0.001629679104	0.0000000001215049628	0.0000000001434300136	0.002589538576	0.0000000001443403623
0.00000875175	0.00001747016	0.001632789179	0.0000000001217368425	0.000000000143703735	0.002592011869	0.0000000001444782232
0.00000876842	0.0000175035	0.001635899254	0.0000000001219687222	0.0000000001439774564	0.002596958457	0.000000000144753945
0.00000878509	0.00001753684	0.001639009328	0.0000000001222006019	0.0000000001442511778	0.002601905045	0.0000000001450296668
0.00000880176	0.00001757018	0.001642119403	0.0000000001224324816	0.0000000001445248992	0.002606851632	0.0000000001453053886
0.00000881843	0.00001762019	0.001645229478	0.0000000001226643613	0.0000000001447986206	0.002614271513	0.0000000001457189713
0.0000088351	0.00001765353	0.001648339552	0.000000000122896241	0.000000000145072342	0.002619218101	0.0000000001459946931
0.00000885177	0.00001768687	0.001651449627	0.0000000001231281207	0.0000000001453460634	0.002624164688	0.0000000001462704149
0.00000886844	0.00001772021	0.001654559701	0.0000000001233600004	0.0000000001456197848	0.002629111276	0.0000000001465461367
0.00000888511	0.00001773688	0.001657669776	0.0000000001235918801	0.0000000001458935062	0.00263158457	0.0000000001466839976
0.00000890178	0.00001777022	0.001660779851	0.0000000001238237598	0.0000000001461672276	0.002636531157	0.0000000001469597194
0.00000891845	0.00001782023	0.001663889925	0.0000000001240556395	0.000000000146440949	0.002643951039	0.0000000001473733021
0.00000893512	0.0000178369	0.001667	0.0000000001242875192	0.0000000001467146704	0.002646424332	0.000000000147511163
0.00000895179	0.00001788691	0.001670110075	0.0000000001245193989	0.0000000001469883918	0.002653844214	0.0000000001479247457
0.00000896846	0.00001790358	0.001673220149	0.0000000001247512786	0.0000000001472621132	0.002656317507	0.0000000001480626066
0.00000898513	0.00001795359	0.001676330224	0.0000000001249831583	0.0000000001475358346	0.002663737389	0.0000000001484761893
0.0000090018	0.00001797026	0.001679440299	0.000000000125215038	0.000000000147809556	0.002666210682	0.0000000001486140502
0.00000901847	0.0000180036	0.001682550373	0.0000000001254469177	0.0000000001480832774	0.00267115727	0.000000000148889772
0.00000903514	0.00001805361	0.001685660448	0.0000000001256787974	0.0000000001483569988	0.002678577151	0.0000000001493033547
0.00000905181	0.00001808695	0.001688770522	0.0000000001259106771	0.0000000001486307202	0.002683523739	0.0000000001495790765
0.00000906848	0.00001810362	0.001691880597	0.0000000001261425568	0.0000000001489044416	0.002685997033	0.0000000001497169374
0.00000908515	0.00001815363	0.001694990672	0.0000000001263744365	0.000000000149178163	0.002693416914	0.0000000001501305201
0.00000910182	0.0000181703	0.001698100746	0.0000000001266063162	0.0000000001494518844	0.002695890208	0.000000000150268381
0.00000911849	0.00001820364	0.001701210821	0.0000000001268381959	0.0000000001497256058	0.002700836795	0.0000000001505441028
0.00000913516	0.00001823698	0.001704320896	0.0000000001270700756	0.0000000001499993272	0.002705783383	0.0000000001508198246
0.00000915183	0.00001828699	0.00170743097	0.0000000001273019553	0.0000000001502730486	0.002713203264	0.0000000001512334073
0.0000091685	0.00001832033	0.001710541045	0.000000000127533835	0.00000000015054677	0.002718149852	0.0000000001515091291
0.00000918517	0.000018337	0.001713651119	0.0000000001277657147	0.0000000001508204914	0.002720623145	0.00000000015164699
0.00000920184	0.00001837034	0.001716761194	0.0000000001279975944	0.0000000001510942128	0.002725569733	0.0000000001519227118
0.00000921851	0.00001842035	0.001719871269	0.0000000001282294741	0.0000000001513679342	0.002732989614	0.0000000001523362945
0.00000923518	0.00001845369	0.001722981343	0.0000000001284613538	0.0000000001516416556	0.002737936202	0.0000000001526120163
0.00000925185	0.00001847036	0.001726091418	0.0000000001286932335	0.000000000151915377	0.002740409496	0.0000000001527498772
0.00000926852	0.0000185037	0.001729201493	0.0000000001289251132	0.0000000001521890984	0.002745356083	0.000000000153025599
0.00000928519	0.00001853704	0.001732311567	0.0000000001291569929	0.0000000001524628198	0.002750302671	0.0000000001533013208
0.00000930186	0.00001857038	0.001735421642	0.0000000001293888726	0.0000000001527365412	0.002755249258	0.0000000001535770426
0.00000931853	0.00001862039	0.001738531716	0.0000000001296207523	0.0000000001530102626	0.002762669139	0.0000000001539906253
0.0000093352	0.00001865373	0.001741641791	0.000000000129852632	0.000000000153283984	0.002767615727	0.0000000001542663471

0.00000935187	0.0000186704	0.001744751866	0.0000000001300845117	0.0000000001535577054	0.002770089021	0.000000000154404208
0.00000936854	0.00001872041	0.00174786194	0.0000000001303163914	0.0000000001538314268	0.002777508902	0.0000000001548177907
0.00000938521	0.00001875375	0.001750972015	0.0000000001305482711	0.0000000001541051482	0.00278245549	0.0000000001550935125
0.00000940188	0.00001878709	0.00175408209	0.0000000001307801508	0.0000000001543788696	0.002787402077	0.0000000001553692343
0.00000941855	0.00001882043	0.001757192164	0.0000000001310120305	0.000000000154652591	0.002792348665	0.0000000001556449561
0.00000943522	0.00001885377	0.001760302239	0.0000000001312439102	0.0000000001549263124	0.002797295252	0.0000000001559206779
0.00000945189	0.00001888711	0.001763412313	0.0000000001314757899	0.0000000001552000338	0.00280224184	0.0000000001561963997
0.00000946856	0.00001892045	0.001766522388	0.0000000001317076696	0.0000000001554737552	0.002807188427	0.0000000001564721215
0.00000948523	0.00001893712	0.001769632463	0.0000000001319395493	0.0000000001557474766	0.002809661721	0.0000000001566099824
0.0000095019	0.00001897046	0.001772742537	0.000000000132171429	0.000000000156021198	0.002814608309	0.0000000001568857042
0.00000951857	0.0000190038	0.001775852612	0.0000000001324033087	0.0000000001562949194	0.002819554896	0.000000000157161426
0.00000953524	0.00001902047	0.001778962687	0.0000000001326351884	0.0000000001565686408	0.00282202819	0.0000000001572992869
0.00000955191	0.00001907048	0.001782072761	0.0000000001328670681	0.0000000001568423622	0.002829448071	0.0000000001577128696
0.00000956858	0.00001908715	0.001785182836	0.0000000001330989478	0.0000000001571160836	0.002831921365	0.0000000001578507305
0.00000958525	0.00001913716	0.00178829291	0.0000000001333308275	0.000000000157389805	0.002839341246	0.0000000001582643132
0.00000960192	0.0000191705	0.001791402985	0.0000000001335627072	0.0000000001576635264	0.002844287834	0.000000000158540035
0.00000961859	0.00001920384	0.00179451306	0.0000000001337945869	0.0000000001579372478	0.002849234421	0.0000000001588157568
0.00000963526	0.00001925385	0.001797623134	0.0000000001340264666	0.0000000001582109692	0.002856654303	0.0000000001592293395
0.00000965193	0.00001927052	0.001800733209	0.0000000001342583463	0.0000000001584846906	0.002859127596	0.0000000001593672004
0.0000096686	0.00001932053	0.001803843284	0.000000000134490226	0.000000000158758412	0.002866547478	0.0000000001597807831
0.00000968527	0.0000193372	0.001806953358	0.0000000001347221057	0.0000000001590321334	0.002869020772	0.000000000159918644
0.00000970194	0.00001938721	0.001810063433	0.0000000001349539854	0.0000000001593058548	0.002876440653	0.0000000001603322267
0.00000971861	0.00001942055	0.001813173507	0.0000000001351858651	0.0000000001595795762	0.00288138724	0.0000000001606079485
0.00000973528	0.00001943722	0.001816283582	0.0000000001354177448	0.0000000001598532976	0.002883860534	0.0000000001607458094
0.00000975195	0.00001948723	0.001819393657	0.0000000001356496245	0.000000000160127019	0.002891280415	0.0000000001611593921
0.00000976862	0.0000195039	0.001822503731	0.0000000001358815042	0.0000000001604007404	0.002893753709	0.000000000161297253
0.00000978529	0.00001953724	0.001825613806	0.0000000001361133839	0.0000000001606744618	0.002898700297	0.0000000001615729748
0.00000980196	0.00001958725	0.001828723881	0.0000000001363452636	0.0000000001609481832	0.002906120178	0.0000000001619865575
0.00000981863	0.00001960392	0.001831833955	0.0000000001365771433	0.0000000001612219046	0.002908593472	0.0000000001621244184
0.0000098353	0.00001965393	0.00183494403	0.000000000136809023	0.000000000161495626	0.002916013353	0.0000000001625380011
0.00000985197	0.0000196706	0.001838054104	0.0000000001370409027	0.0000000001617693474	0.002918486647	0.000000000162675862
0.00000986864	0.00001970394	0.001841164179	0.0000000001372727824	0.0000000001620430688	0.002923433234	0.0000000001629515838
0.00000988531	0.00001975395	0.001844274254	0.0000000001375046621	0.0000000001623167902	0.002930853116	0.0000000001633651665
0.00000990198	0.00001977062	0.001847384328	0.0000000001377365418	0.0000000001625905116	0.002933326409	0.0000000001635030274
0.00000991865	0.00001978729	0.001850494403	0.0000000001379684215	0.000000000162864233	0.002935799703	0.0000000001636408883
0.00000993532	0.00001982063	0.001853604478	0.0000000001382003012	0.0000000001631379544	0.002940746291	0.0000000001639166101
0.00000995199	0.00001987064	0.001856714552	0.0000000001384321809	0.0000000001634116758	0.002948166172	0.0000000001643301928
0.00000996866	0.00001992065	0.001859824627	0.0000000001386640606	0.0000000001636853972	0.002955586053	0.0000000001647437755
0.00000998533	0.00001995399	0.001862934701	0.0000000001388959403	0.0000000001639591186	0.002960532641	0.0000000001650194973
0.000010002	0.00001998733	0.001866044776	0.00000000013912782	0.00000000016423284	0.002965479228	0.0000000001652952191

0.00001001867	0.00002002067	0.001869154851	0.0000000001393596997	0.0000000001645065614	0.002970425816	0.0000000001655709409
0.00001003534	0.00002005401	0.001872264925	0.0000000001395915794	0.0000000001647802828	0.002975372404	0.0000000001658466627
0.00001005201	0.00002007068	0.001875375	0.0000000001398234591	0.0000000001650540042	0.002977845697	0.0000000001659845236
0.00001006868	0.00002010402	0.001878485075	0.0000000001400553388	0.0000000001653277256	0.002982792285	0.0000000001662602454
0.00001008535	0.00002015403	0.001881595149	0.0000000001402872185	0.000000000165601447	0.002990212166	0.0000000001666738281
0.00001010202	0.0000201707	0.001884705224	0.0000000001405190982	0.0000000001658751684	0.00299268546	0.000000000166811689
0.00001011869	0.00002020404	0.001887815299	0.0000000001407509779	0.0000000001661488898	0.002997632047	0.0000000001670874108
0.00001013536	0.00002023738	0.001890925373	0.0000000001409828576	0.0000000001664226112	0.003002578635	0.0000000001673631326
0.00001015203	0.00002028739	0.001894035448	0.0000000001412147373	0.0000000001666963326	0.003009998516	0.0000000001677767153
0.0000101687	0.00002030406	0.001897145522	0.000000000141446617	0.000000000166970054	0.00301247181	0.0000000001679145762
0.00001018537	0.0000203374	0.001900255597	0.0000000001416784967	0.0000000001672437754	0.003017418398	0.000000000168190298
0.00001020204	0.00002038741	0.001903365672	0.0000000001419103764	0.0000000001675174968	0.003024838279	0.0000000001686038807
0.00001021871	0.00002040408	0.001906475746	0.0000000001421422561	0.0000000001677912182	0.003027311573	0.0000000001687417416
0.00001023538	0.00002043742	0.001909585821	0.0000000001423741358	0.0000000001680649396	0.00303225816	0.0000000001690174634
0.00001025205	0.00002048743	0.001912695896	0.0000000001426060155	0.000000000168338661	0.003039678042	0.0000000001694310461
0.00001026872	0.0000205041	0.00191580597	0.0000000001428378952	0.0000000001686123824	0.003042151335	0.000000000169568907
0.00001028539	0.00002053744	0.001918916045	0.0000000001430697749	0.0000000001688861038	0.003047097923	0.0000000001698446288
0.00001030206	0.00002058745	0.001922026119	0.0000000001433016546	0.0000000001691598252	0.003054517804	0.0000000001702582115
0.00001031873	0.00002062079	0.001925136194	0.0000000001435335343	0.0000000001694335466	0.003059464392	0.0000000001705339333
0.0000103354	0.00002065413	0.001928246269	0.000000000143765414	0.000000000169707268	0.003064410979	0.0000000001708096551
0.00001035207	0.00002068747	0.001931356343	0.0000000001439972937	0.0000000001699809894	0.003069357567	0.0000000001710853769
0.00001036874	0.00002070414	0.001934466418	0.0000000001442291734	0.0000000001702547108	0.003071830861	0.0000000001712232378
0.00001038541	0.00002073748	0.001937576493	0.0000000001444610531	0.0000000001705284322	0.003076777448	0.0000000001714989596
0.00001040208	0.00002078749	0.001940686567	0.0000000001446929328	0.0000000001708021536	0.003084197329	0.0000000001719125423
0.00001041875	0.00002080416	0.001943796642	0.0000000001449248125	0.000000000171075875	0.003086670623	0.0000000001720504032
0.00001043542	0.00002085417	0.001946906716	0.0000000001451566922	0.0000000001713495964	0.003094090504	0.0000000001724639859
0.00001045209	0.00002088751	0.001950016791	0.0000000001453885719	0.0000000001716233178	0.003099037092	0.0000000001727397077
0.00001046876	0.00002090418	0.001953126866	0.0000000001456204516	0.0000000001718970392	0.003101510386	0.0000000001728775686
0.00001048543	0.00002093752	0.00195623694	0.0000000001458523313	0.0000000001721707606	0.003106456973	0.0000000001731532904
0.0000105021	0.00002098753	0.001959347015	0.000000000146084211	0.000000000172444482	0.003113876855	0.0000000001735668731
0.00001051877	0.00002102087	0.00196245709	0.0000000001463160907	0.0000000001727182034	0.003118823442	0.0000000001738425949
0.00001053544	0.00002103754	0.001965567164	0.0000000001465479704	0.0000000001729919248	0.003121296736	0.0000000001739804558
0.00001055211	0.00002108755	0.001968677239	0.0000000001467798501	0.0000000001732656462	0.003128716617	0.0000000001743940385
0.00001056878	0.00002110422	0.001971787313	0.0000000001470117298	0.0000000001735393676	0.003131189911	0.0000000001745318994
0.00001058545	0.00002115423	0.001974897388	0.0000000001472436095	0.000000000173813089	0.003138609792	0.0000000001749454821
0.00001060212	0.0000211709	0.001978007463	0.0000000001474754892	0.0000000001740868104	0.003141083086	0.000000000175083343
0.00001061879	0.00002122091	0.001981117537	0.0000000001477073689	0.0000000001743605318	0.003148502967	0.0000000001754969257
0.00001063546	0.00002123758	0.001984227612	0.0000000001479392486	0.0000000001746342532	0.003150976261	0.0000000001756347866
0.00001065213	0.00002128759	0.001987337687	0.0000000001481711283	0.0000000001749079746	0.003158396142	0.0000000001760483693
0.0000106688	0.00002132093	0.001990447761	0.000000000148403008	0.000000000175181696	0.00316334273	0.0000000001763240911

0.00001068547	0.00002135427	0.001993557836	0.0000000001486348877	0.0000000001754554174	0.003168289318	0.0000000001765998129
0.00001070214	0.00002138761	0.00199666791	0.0000000001488667674	0.0000000001757291388	0.003173235905	0.0000000001768755347
0.00001071881	0.00002140428	0.001999777985	0.0000000001490986471	0.0000000001760028602	0.003175709199	0.0000000001770133956
0.00001073548	0.00002145429	0.00200288806	0.0000000001493305268	0.0000000001762765816	0.00318312908	0.0000000001774269783
0.00001075215	0.00002147096	0.002005998134	0.0000000001495624065	0.000000000176550303	0.003185602374	0.0000000001775648392
0.00001076882	0.00002152097	0.002009108209	0.0000000001497942862	0.0000000001768240244	0.003193022255	0.0000000001779784219
0.00001078549	0.00002153764	0.002012218284	0.0000000001500261659	0.0000000001770977458	0.003195495549	0.0000000001781162828
0.00001080216	0.00002157098	0.002015328358	0.0000000001502580456	0.0000000001773714672	0.003200442136	0.0000000001783920046
0.00001081883	0.00002162099	0.002018438433	0.0000000001504899253	0.0000000001776451886	0.003207862018	0.0000000001788055873
0.0000108355	0.00002163766	0.002021548507	0.000000000150721805	0.00000000017791891	0.003210335312	0.0000000001789434482
0.00001085217	0.00002168767	0.002024658582	0.0000000001509536847	0.0000000001781926314	0.003217755193	0.0000000001793570309
0.00001086884	0.00002170434	0.002027768657	0.0000000001511855644	0.0000000001784663528	0.003220228487	0.0000000001794948918
0.00001088551	0.00002172101	0.002030878731	0.0000000001514174441	0.0000000001787400742	0.00322270178	0.0000000001796327527
0.00001090218	0.00002175435	0.002033988806	0.0000000001516493238	0.0000000001790137956	0.003227648368	0.0000000001799084745
0.00001091885	0.00002180436	0.002037098881	0.0000000001518812035	0.000000000179287517	0.003235068249	0.0000000001803220572
0.00001093552	0.0000218377	0.002040208955	0.0000000001521130832	0.0000000001795612384	0.003240014837	0.000000000180597779
0.00001095219	0.00002187104	0.00204331903	0.0000000001523449629	0.0000000001798349598	0.003244961424	0.0000000001808735008
0.00001096886	0.00002190438	0.002046429104	0.0000000001525768426	0.0000000001801086812	0.003249908012	0.0000000001811492226
0.00001098553	0.00002195439	0.002049539179	0.0000000001528087223	0.0000000001803824026	0.003257327893	0.0000000001815628053
0.0000110022	0.00002197106	0.002052649254	0.000000000153040602	0.000000000180656124	0.003259801187	0.0000000001817006662
0.00001101887	0.00002202107	0.002055759328	0.0000000001532724817	0.0000000001809298454	0.003267221068	0.0000000001821142489
0.00001103554	0.00002205441	0.002058869403	0.0000000001535043614	0.0000000001812035668	0.003272167656	0.0000000001823899707
0.00001105221	0.00002208775	0.002061979478	0.0000000001537362411	0.0000000001814772882	0.003277114243	0.0000000001826656925
0.00001106888	0.00002210442	0.002065089552	0.0000000001539681208	0.0000000001817510096	0.003279587537	0.0000000001828035534
0.00001108555	0.00002213776	0.002068199627	0.0000000001542000005	0.000000000182024731	0.003284534125	0.0000000001830792752
0.00001110222	0.00002215443	0.002071309701	0.0000000001544318802	0.0000000001822984524	0.003287007418	0.0000000001832171361
0.00001111889	0.00002220444	0.002074419776	0.0000000001546637599	0.0000000001825721738	0.0032944273	0.0000000001836307188
0.00001113556	0.00002222111	0.002077529851	0.0000000001548956396	0.0000000001828458952	0.003296900593	0.0000000001837685797
0.00001115223	0.00002227112	0.002080639925	0.0000000001551275193	0.0000000001831196166	0.003304320475	0.0000000001841821624
0.0000111689	0.00002228779	0.00208375	0.000000000155359399	0.000000000183393338	0.003306793769	0.0000000001843200233
0.00001118557	0.00002230446	0.002086860075	0.0000000001555912787	0.0000000001836670594	0.003309267062	0.0000000001844578842
0.00001120224	0.0000223378	0.002089970149	0.0000000001558231584	0.0000000001839407808	0.00331421365	0.000000000184733606
0.00001121891	0.00002235447	0.002093080224	0.0000000001560550381	0.0000000001842145022	0.003316686944	0.0000000001848714669
0.00001123558	0.00002240448	0.002096190299	0.0000000001562869178	0.0000000001844882236	0.003324106825	0.0000000001852850496
0.00001125225	0.00002245449	0.002099300373	0.0000000001565187975	0.000000000184761945	0.003331526706	0.0000000001856986323
0.00001126892	0.00002248783	0.002102410448	0.0000000001567506772	0.0000000001850356664	0.003336473294	0.0000000001859743541
0.00001128559	0.0000225045	0.002105520522	0.0000000001569825569	0.0000000001853093878	0.003338946588	0.000000000186112215
0.00001130226	0.00002257118	0.002108630597	0.0000000001572144366	0.0000000001855831092	0.003348839763	0.0000000001866636586
0.00001131893	0.00002258785	0.002111740672	0.0000000001574463163	0.0000000001858568306	0.003351313056	0.0000000001868015195
0.0000113356	0.00002263786	0.002114850746	0.000000000157678196	0.000000000186130552	0.003358732938	0.0000000001872151022

0.00001135227	0.00002268787	0.002117960821	0.0000000001579100757	0.0000000001864042734	0.003366152819	0.0000000001876286849
0.00001136894	0.00002272121	0.002121070896	0.0000000001581419554	0.0000000001866779948	0.003371099407	0.0000000001879044067
0.00001138561	0.00002275455	0.00212418097	0.0000000001583738351	0.0000000001869517162	0.003376045994	0.0000000001881801285
0.00001140228	0.00002277122	0.002127291045	0.0000000001586057148	0.0000000001872254376	0.003378519288	0.0000000001883179894
0.00001141895	0.00002282123	0.002130401119	0.0000000001588375945	0.000000000187499159	0.003385939169	0.0000000001887315721
0.00001143562	0.0000228379	0.002133511194	0.0000000001590694742	0.0000000001877728804	0.003388412463	0.000000000188869433
0.00001145229	0.00002285457	0.002136621269	0.0000000001593013539	0.0000000001880466018	0.003390885757	0.0000000001890072939
0.00001146896	0.00002288791	0.002139731343	0.0000000001595332336	0.0000000001883203232	0.003395832344	0.0000000001892830157
0.00001148563	0.00002292125	0.002142841418	0.0000000001597651133	0.0000000001885940446	0.003400778932	0.0000000001895587375
0.0000115023	0.00002297126	0.002145951493	0.000000000159996993	0.000000000188867766	0.003408198813	0.0000000001899723202
0.00001151897	0.00002298793	0.002149061567	0.0000000001602288727	0.0000000001891414874	0.003410672107	0.000000000190110181
0.00001153564	0.00002302127	0.002152171642	0.0000000001604607524	0.0000000001894152088	0.003415618694	0.0000000001903859029
0.00001155231	0.00002305461	0.002155281716	0.0000000001606926321	0.0000000001896889302	0.003420565282	0.0000000001906616247
0.00001156898	0.00002310462	0.002158391791	0.0000000001609245118	0.0000000001899626516	0.003427985163	0.0000000001910752074
0.00001158565	0.00002313796	0.002161501866	0.0000000001611563915	0.000000000190236373	0.003432931751	0.0000000001913509292
0.00001160232	0.00002315463	0.00216461194	0.0000000001613882712	0.0000000001905100944	0.003435405045	0.0000000001914887901
0.00001161899	0.00002320464	0.002167722015	0.0000000001616201509	0.0000000001907838158	0.003442824926	0.0000000001919023728
0.00001163566	0.00002325465	0.00217083209	0.0000000001618520306	0.0000000001910575372	0.003450244807	0.0000000001923159555
0.00001165233	0.00002328799	0.002173942164	0.0000000001620839103	0.0000000001913312586	0.003455191395	0.0000000001925916773
0.000011669	0.00002332133	0.002177052239	0.00000000016231579	0.00000000019160498	0.003460137982	0.0000000001928673991
0.00001168567	0.000023338	0.002180162313	0.0000000001625476697	0.0000000001918787014	0.003462611276	0.00000000019300526
0.00001170234	0.00002335467	0.002183272388	0.0000000001627795494	0.0000000001921524228	0.00346508457	0.0000000001931431209
0.00001171901	0.00002338801	0.002186382463	0.0000000001630114291	0.0000000001924261442	0.003470031157	0.0000000001934188427
0.00001173568	0.00002342135	0.002189492537	0.0000000001632433088	0.0000000001926998656	0.003474977745	0.0000000001936945645
0.00001175235	0.00002343802	0.002192602612	0.0000000001634751885	0.000000000192973587	0.003477451039	0.0000000001938324254
0.00001176902	0.00002347136	0.002195712687	0.0000000001637070682	0.0000000001932473084	0.003482397626	0.0000000001941081472
0.00001178569	0.0000235047	0.002198822761	0.0000000001639389479	0.0000000001935210298	0.003487344214	0.000000000194383869
0.00001180236	0.00002353804	0.002201932836	0.0000000001641708276	0.0000000001937947512	0.003492290801	0.0000000001946595908
0.00001181903	0.00002357138	0.00220504291	0.0000000001644027073	0.0000000001940684726	0.003497237389	0.0000000001949353126
0.0000118357	0.00002362139	0.002208152985	0.000000000164634587	0.000000000194342194	0.00350465727	0.0000000001953488953
0.00001185237	0.00002365473	0.00221126306	0.0000000001648664667	0.0000000001946159154	0.003509603858	0.0000000001956246171
0.00001186904	0.00002368807	0.002214373134	0.0000000001650983464	0.0000000001948896368	0.003514550445	0.0000000001959003389
0.00001188571	0.00002372141	0.002217483209	0.0000000001653302261	0.0000000001951633582	0.003519497033	0.0000000001961760607
0.00001190238	0.00002375475	0.002220593284	0.0000000001655621058	0.0000000001954370796	0.00352444362	0.0000000001964517825
0.00001191905	0.00002378809	0.002223703358	0.0000000001657939855	0.000000000195710801	0.003529390208	0.0000000001967275043
0.00001193572	0.00002380476	0.002226813433	0.0000000001660258652	0.0000000001959845224	0.003531863501	0.0000000001968653652
0.00001195239	0.0000238381	0.002229923507	0.0000000001662577449	0.0000000001962582438	0.003536810089	0.000000000197141087
0.00001196906	0.00002387144	0.002233033582	0.0000000001664896246	0.0000000001965319652	0.003541756677	0.0000000001974168088
0.00001198573	0.00002390478	0.002236143657	0.0000000001667215043	0.0000000001968056866	0.003546703264	0.0000000001976925306
0.0000120024	0.00002395479	0.002239253731	0.000000000166953384	0.000000000197079408	0.003554123145	0.0000000001981061133

0.00001201907	0.00002397146	0.002242363806	0.0000000001671852637	0.0000000001973531294	0.003556596439	0.0000000001982439742
0.00001203574	0.0000240048	0.002245473881	0.0000000001674171434	0.0000000001976268508	0.003561543027	0.000000000198519696
0.00001205241	0.00002405481	0.002248583955	0.0000000001676490231	0.0000000001979005722	0.003568962908	0.0000000001989332787
0.00001206908	0.00002408815	0.00225169403	0.0000000001678809028	0.0000000001981742936	0.003573909496	0.0000000001992090005
0.00001208575	0.00002413816	0.002254804104	0.0000000001681127825	0.000000000198448015	0.003581329377	0.0000000001996225832
0.00001210242	0.00002415483	0.002257914179	0.0000000001683446622	0.0000000001987217364	0.003583802671	0.0000000001997604441
0.00001211909	0.0000241715	0.002261024254	0.0000000001685765419	0.0000000001989954578	0.003586275964	0.000000000199898305
0.00001213576	0.00002420484	0.002264134328	0.0000000001688084216	0.0000000001992691792	0.003591222552	0.0000000002001740268
0.00001215243	0.00002423818	0.002267244403	0.0000000001690403013	0.0000000001995429006	0.003596169139	0.0000000002004497486
0.0000121691	0.00002427152	0.002270354478	0.000000000169272181	0.000000000199816622	0.003601115727	0.0000000002007254704
0.00001218577	0.00002430486	0.002273464552	0.0000000001695040607	0.0000000002000903434	0.003606062315	0.0000000002010011922
0.00001220244	0.00002435487	0.002276574627	0.0000000001697359404	0.0000000002003640648	0.003613482196	0.0000000002014147749
0.00001221911	0.00002438821	0.002279684701	0.0000000001699678201	0.0000000002006377862	0.003618428783	0.0000000002016904967
0.00001223578	0.00002443822	0.002282794776	0.0000000001701996998	0.0000000002009115076	0.003625848665	0.0000000002021040794
0.00001225245	0.00002445489	0.002285904851	0.0000000001704315795	0.000000000201185229	0.003628321958	0.0000000002022419403
0.00001226912	0.00002448823	0.002289014925	0.0000000001706634592	0.0000000002014589504	0.003633268546	0.0000000002025176621
0.00001228579	0.0000245049	0.002292125	0.0000000001708953389	0.0000000002017326718	0.00363574184	0.000000000202655523
0.00001230246	0.00002455491	0.002295235075	0.0000000001711272186	0.0000000002020063932	0.003643161721	0.0000000002030691057
0.00001231913	0.00002458825	0.002298345149	0.0000000001713590983	0.0000000002022801146	0.003648108309	0.0000000002033448275
0.0000123358	0.00002463826	0.002301455224	0.000000000171590978	0.000000000202553836	0.00365552819	0.0000000002037584102
0.00001235247	0.0000246716	0.002304565299	0.0000000001718228577	0.0000000002028275574	0.003660474777	0.000000000204034132
0.00001236914	0.00002470494	0.002307675373	0.0000000001720547374	0.0000000002031012788	0.003665421365	0.0000000002043098538
0.00001238581	0.00002472161	0.002310785448	0.0000000001722866171	0.0000000002033750002	0.003667894659	0.0000000002044477147
0.00001240248	0.00002475495	0.002313895522	0.0000000001725184968	0.0000000002036487216	0.003672841246	0.0000000002047234365
0.00001241915	0.00002480496	0.002317005597	0.0000000001727503765	0.000000000203922443	0.003680261128	0.0000000002051370192
0.00001243582	0.0000248383	0.002320115672	0.0000000001729822562	0.0000000002041961644	0.003685207715	0.000000000205412741
0.00001245249	0.00002487164	0.002323225746	0.0000000001732141359	0.0000000002044698858	0.003690154303	0.0000000002056884628
0.00001246916	0.00002488831	0.002326335821	0.0000000001734460156	0.0000000002047436072	0.003692627596	0.0000000002058263237
0.00001248583	0.00002492165	0.002329445896	0.0000000001736778953	0.0000000002050173286	0.003697574184	0.0000000002061020455
0.0000125025	0.00002498833	0.00233255597	0.000000000173909775	0.00000000020529105	0.003707467359	0.0000000002066534891
0.00001251917	0.000025005	0.002335666045	0.0000000001741416547	0.0000000002055647714	0.003709940653	0.00000000020679135
0.00001253584	0.00002502167	0.002338776119	0.0000000001743735344	0.0000000002058384928	0.003712413947	0.0000000002069292109
0.00001255251	0.00002507168	0.002341886194	0.0000000001746054141	0.0000000002061122142	0.003719833828	0.0000000002073427936
0.00001256918	0.00002512169	0.002344996269	0.0000000001748372938	0.0000000002063859356	0.003727253709	0.0000000002077563763
0.00001258585	0.00002513836	0.002348106343	0.0000000001750691735	0.000000000206659657	0.003729727003	0.0000000002078942372
0.00001260252	0.0000251717	0.002351216418	0.0000000001753010532	0.0000000002069333784	0.003734673591	0.000000000208169959
0.00001261919	0.00002520504	0.002354326493	0.0000000001755329329	0.0000000002072070998	0.003739620178	0.0000000002084456808
0.00001263586	0.00002523838	0.002357436567	0.0000000001757648126	0.0000000002074808212	0.003744566766	0.0000000002087214026
0.00001265253	0.00002528839	0.002360546642	0.0000000001759966923	0.0000000002077545426	0.003751986647	0.0000000002091349853
0.0000126692	0.00002532173	0.002363656716	0.000000000176228572	0.000000000208028264	0.003756933234	0.0000000002094107071

0.00001268587	0.00002535507	0.002366766791	0.0000000001764604517	0.0000000002083019854	0.003761879822	0.0000000002096864289
0.00001270254	0.00002538841	0.002369876866	0.0000000001766923314	0.0000000002085757068	0.003766826409	0.0000000002099621507
0.00001271921	0.00002542175	0.00237298694	0.0000000001769242111	0.0000000002088494282	0.003771772997	0.0000000002102378725
0.00001273588	0.00002543842	0.002376097015	0.0000000001771560908	0.0000000002091231496	0.003774246291	0.0000000002103757334
0.00001275255	0.00002548843	0.00237920709	0.0000000001773879705	0.000000000209396871	0.003781666172	0.0000000002107893161
0.00001276922	0.0000255051	0.002382317164	0.0000000001776198502	0.0000000002096705924	0.003784139466	0.000000000210927177
0.00001278589	0.00002552177	0.002385427239	0.0000000001778517299	0.0000000002099443138	0.00378661276	0.0000000002110650379
0.00001280256	0.00002553844	0.002388537313	0.0000000001780836096	0.0000000002102180352	0.003789086053	0.0000000002112028988
0.00001281923	0.00002558845	0.002391647388	0.0000000001783154893	0.0000000002104917566	0.003796505935	0.0000000002116164815
0.0000128359	0.00002563846	0.002394757463	0.000000000178547369	0.000000000210765478	0.003803925816	0.0000000002120300642
0.00001285257	0.00002565513	0.002397867537	0.0000000001787792487	0.0000000002110391994	0.00380639911	0.0000000002121679251
0.00001286924	0.00002568847	0.002400977612	0.0000000001790111284	0.0000000002113129208	0.003811345697	0.0000000002124436469
0.00001288591	0.00002573848	0.002404087687	0.0000000001792430081	0.0000000002115866422	0.003818765579	0.0000000002128572296
0.00001290258	0.00002578849	0.002407197761	0.0000000001794748878	0.0000000002118603636	0.00382618546	0.0000000002132708123
0.00001291925	0.00002580516	0.002410307836	0.0000000001797067675	0.000000000212134085	0.003828658754	0.0000000002134086732
0.00001293592	0.00002585517	0.00241341791	0.0000000001799386472	0.0000000002124078064	0.003836078635	0.0000000002138222559
0.00001295259	0.00002588851	0.002416527985	0.0000000001801705269	0.0000000002126815278	0.003841025223	0.0000000002140979777
0.00001296926	0.00002590518	0.00241963806	0.0000000001804024066	0.0000000002129552492	0.003843498516	0.0000000002142358386
0.00001298593	0.00002592185	0.002422748134	0.0000000001806342863	0.0000000002132289706	0.00384597181	0.0000000002143736995
0.0000130026	0.00002595519	0.002425858209	0.000000000180866166	0.000000000213502692	0.003850918398	0.0000000002146494213
0.00001301927	0.00002598853	0.002428968284	0.0000000001810980457	0.0000000002137764134	0.003855864985	0.0000000002149251431
0.00001303594	0.00002602187	0.002432078358	0.0000000001813299254	0.0000000002140501348	0.003860811573	0.0000000002152008649
0.00001305261	0.00002603854	0.002435188433	0.0000000001815618051	0.0000000002143238562	0.003863284866	0.0000000002153387258
0.00001306928	0.00002610522	0.002438298507	0.0000000001817936848	0.0000000002145975776	0.003873178042	0.0000000002158901694
0.00001308595	0.00002613856	0.002441408582	0.0000000001820255645	0.000000000214871299	0.003878124629	0.0000000002161658912
0.00001310262	0.00002615523	0.002444518657	0.0000000001822574442	0.0000000002151450204	0.003880597923	0.0000000002163037521
0.00001311929	0.00002620524	0.002447628731	0.0000000001824893239	0.0000000002154187418	0.003888017804	0.0000000002167173348
0.00001313596	0.00002622191	0.002450738806	0.0000000001827212036	0.0000000002156924632	0.003890491098	0.0000000002168551957
0.00001315263	0.00002625525	0.002453848881	0.0000000001829530833	0.0000000002159661846	0.003895437685	0.0000000002171309175
0.0000131693	0.00002628859	0.002456958955	0.000000000183184963	0.000000000216239906	0.003900384273	0.0000000002174066393
0.00001318597	0.00002630526	0.00246006903	0.0000000001834168427	0.0000000002165136274	0.003902857567	0.0000000002175445002
0.00001320264	0.0000263386	0.002463179104	0.0000000001836487224	0.0000000002167873488	0.003907804154	0.000000000217820222
0.00001321931	0.00002638861	0.002466289179	0.0000000001838806021	0.0000000002170610702	0.003915224036	0.0000000002182338047
0.00001323598	0.00002645529	0.002469399254	0.0000000001841124818	0.0000000002173347916	0.003925117211	0.0000000002187852483
0.00001325265	0.00002648863	0.002472509328	0.0000000001843443615	0.000000000217608513	0.003930063798	0.0000000002190609701
0.00001326932	0.00002652197	0.002475619403	0.0000000001845762412	0.0000000002178822344	0.003935010386	0.0000000002193366919
0.00001328599	0.00002653864	0.002478729478	0.0000000001848081209	0.0000000002181559558	0.00393748368	0.0000000002194745528
0.00001330266	0.00002657198	0.002481839552	0.0000000001850400006	0.0000000002184296772	0.003942430267	0.0000000002197502746
0.00001331933	0.00002660532	0.002484949627	0.0000000001852718803	0.0000000002187033986	0.003947376855	0.0000000002200259964
0.000013336	0.00002663866	0.002488059701	0.00000000018550376	0.00000000021897712	0.003952323442	0.0000000002203017182

0.00001335267	0.00002665533	0.002491169776	0.0000000001857356397	0.0000000002192508414	0.003954796736	0.0000000002204395791
0.00001336934	0.000026672	0.002494279851	0.0000000001859675194	0.0000000002195245628	0.00395727003	0.00000000022057744
0.00001338601	0.00002670534	0.002497389925	0.0000000001861993991	0.0000000002197982842	0.003962216617	0.0000000002208531618
0.00001340268	0.00002675535	0.0025005	0.0000000001864312788	0.0000000002200720056	0.003969636499	0.0000000002212667445
0.00001341935	0.00002678869	0.002503610075	0.0000000001866631585	0.000000000220345727	0.003974583086	0.0000000002215424663
0.00001343602	0.00002682203	0.002506720149	0.0000000001868950382	0.0000000002206194484	0.003979529674	0.0000000002218181881
0.00001345269	0.00002688871	0.002509830224	0.0000000001871269179	0.0000000002208931698	0.003989422849	0.0000000002223696317
0.00001346936	0.00002690538	0.002512940299	0.0000000001873587976	0.0000000002211668912	0.003991896142	0.0000000002225074926
0.00001348603	0.00002692205	0.002516050373	0.0000000001875906773	0.0000000002214406126	0.003994369436	0.0000000002226453535
0.0000135027	0.00002697206	0.002519160448	0.000000000187822557	0.000000000221714334	0.004001789318	0.0000000002230589362
0.00001351937	0.0000270054	0.002522270522	0.0000000001880544367	0.0000000002219880554	0.004006735905	0.000000000223334658
0.00001353604	0.00002705541	0.002525380597	0.0000000001882863164	0.0000000002222617768	0.004014155786	0.0000000002237482407
0.00001355271	0.00002723878	0.002528490672	0.0000000001885181961	0.0000000002225354982	0.004041362018	0.0000000002252647106
0.00001356938	0.00002710542	0.002531600746	0.0000000001887500758	0.0000000002228092196	0.004021575668	0.0000000002241618234
0.00001358605	0.00002713876	0.002534710821	0.0000000001889819555	0.000000000223082941	0.004026522255	0.0000000002244375452
0.00001360272	0.0000271721	0.002537820896	0.0000000001892138352	0.0000000002233566624	0.004031468843	0.000000000224713267
0.00001361939	0.00002718877	0.00254093097	0.0000000001894457149	0.0000000002236303838	0.004033942136	0.0000000002248511279
0.00001363606	0.00002723878	0.002544041045	0.0000000001896775946	0.0000000002239041052	0.004041362018	0.0000000002252647106
0.00001365273	0.00002727212	0.002547151119	0.0000000001899094743	0.0000000002241778266	0.004046308605	0.0000000002255404324
0.0000136694	0.00002730546	0.002550261194	0.000000000190141354	0.000000000224451548	0.004051255193	0.0000000002258161542
0.00001368607	0.0000273388	0.002553371269	0.0000000001903732337	0.0000000002247252694	0.00405620178	0.000000000226091876
0.00001370274	0.00002737214	0.002556481343	0.0000000001906051134	0.0000000002249989908	0.004061148368	0.0000000002263675978
0.00001371941	0.00002740548	0.002559591418	0.0000000001908369931	0.0000000002252727122	0.004066094955	0.0000000002266433196
0.00001373608	0.00002743882	0.002562701493	0.0000000001910688728	0.0000000002255464336	0.004071041543	0.0000000002269190414
0.00001375275	0.00002747216	0.002565811567	0.0000000001913007525	0.000000000225820155	0.004075988131	0.0000000002271947632
0.00001376942	0.00002752217	0.002568921642	0.0000000001915326322	0.0000000002260938764	0.004083408012	0.0000000002276083459
0.00001378609	0.00002755551	0.002572031716	0.0000000001917645119	0.0000000002263675978	0.004088354599	0.0000000002278840677
0.00001380276	0.00002758885	0.002575141791	0.0000000001919963916	0.0000000002266413192	0.004093301187	0.0000000002281597895
0.00001381943	0.00002762219	0.002578251866	0.0000000001922282713	0.0000000002269150406	0.004098247774	0.0000000002284355113
0.0000138361	0.00002765553	0.00258136194	0.000000000192460151	0.000000000227188762	0.004103194362	0.0000000002287112331
0.00001385277	0.00002768887	0.002584472015	0.0000000001926920307	0.0000000002274624834	0.00410814095	0.0000000002289869549
0.00001386944	0.00002772221	0.00258758209	0.0000000001929239104	0.0000000002277362048	0.004113087537	0.0000000002292626767
0.00001388611	0.00002775555	0.002590692164	0.0000000001931557901	0.0000000002280099262	0.004118034125	0.0000000002295383985
0.00001390278	0.00002777222	0.002593802239	0.0000000001933876698	0.0000000002282836476	0.004120507418	0.0000000002296762594
0.00001391945	0.00002782223	0.002596912313	0.0000000001936195495	0.000000000228557369	0.0041279273	0.0000000002300898421
0.00001393612	0.00002785557	0.002600022388	0.0000000001938514292	0.0000000002288310904	0.004132873887	0.0000000002303655639
0.00001395279	0.00002787224	0.002603132463	0.0000000001940833089	0.0000000002291048118	0.004135347181	0.0000000002305034248
0.00001396946	0.00002790558	0.002606242537	0.0000000001943151886	0.0000000002293785332	0.004140293769	0.0000000002307791466
0.00001398613	0.00002795559	0.002609352612	0.0000000001945470683	0.0000000002296522546	0.00414771365	0.0000000002311927293
0.0000140028	0.00002797226	0.002612462687	0.000000000194778948	0.000000000229925976	0.004150186944	0.0000000002313305902

0.00001401947	0.0000280056	0.002615572761	0.0000000001950108277	0.0000000002301996974	0.004155133531	0.000000000231606312
0.00001403614	0.00002803894	0.002618682836	0.0000000001952427074	0.0000000002304734188	0.004160080119	0.0000000002318820338
0.00001405281	0.00002808895	0.00262179291	0.0000000001954745871	0.0000000002307471402	0.0041675	0.0000000002322956165
0.00001406948	0.00002812229	0.002624902985	0.0000000001957064668	0.0000000002310208616	0.004172446588	0.0000000002325713383
0.00001408615	0.00002813896	0.00262801306	0.0000000001959383465	0.000000000231294583	0.004174919881	0.0000000002327091992
0.00001410282	0.00002818897	0.002631123134	0.0000000001961702262	0.0000000002315683044	0.004182339763	0.0000000002331227819
0.00001411949	0.00002820564	0.002634233209	0.0000000001964021059	0.0000000002318420258	0.004184813056	0.0000000002332606428
0.00001413616	0.00002825565	0.002637343284	0.0000000001966339856	0.0000000002321157472	0.004192232938	0.0000000002336742255
0.00001415283	0.00002827232	0.002640453358	0.0000000001968658653	0.0000000002323894686	0.004194706231	0.0000000002338120864
0.0000141695	0.00002832233	0.002643563433	0.000000000197097745	0.00000000023266319	0.004202126113	0.0000000002342256691
0.00001418617	0.00002835567	0.002646673507	0.0000000001973296247	0.0000000002329369114	0.0042070727	0.0000000002345013909
0.00001420284	0.00002837234	0.002649783582	0.0000000001975615044	0.0000000002332106328	0.004209545994	0.0000000002346392518
0.00001421951	0.00002842235	0.002652893657	0.0000000001977933841	0.0000000002334843542	0.004216965875	0.0000000002350528345
0.00001423618	0.00002845569	0.002656003731	0.0000000001980252638	0.0000000002337580756	0.004221912463	0.0000000002353285563
0.00001425285	0.00002847236	0.002659113806	0.0000000001982571435	0.000000000234031797	0.004224385757	0.0000000002354664172
0.00001426952	0.00002852237	0.002662223881	0.0000000001984890232	0.0000000002343055184	0.004231805638	0.0000000002358799999
0.00001428619	0.00002855571	0.002665333955	0.0000000001987209029	0.0000000002345792398	0.004236752226	0.0000000002361557217
0.00001430286	0.00002858905	0.00266844403	0.0000000001989527826	0.0000000002348529612	0.004241698813	0.0000000002364314435
0.00001431953	0.00002860572	0.002671554104	0.0000000001991846623	0.0000000002351266826	0.004244172107	0.0000000002365693044
0.0000143362	0.00002863906	0.002674664179	0.000000000199416542	0.000000000235400404	0.004249118694	0.0000000002368450262
0.00001435287	0.0000286724	0.002677774254	0.0000000001996484217	0.0000000002356741254	0.004254065282	0.000000000237120748
0.00001436954	0.00002872241	0.002680884328	0.0000000001998803014	0.0000000002359478468	0.004261485163	0.0000000002375343307
0.00001438621	0.00002873908	0.002683994403	0.000000000200112181	0.0000000002362215682	0.004263958457	0.0000000002376721916
0.00001440288	0.00002878909	0.002687104478	0.0000000002003440608	0.0000000002364952896	0.004271378338	0.0000000002380857743
0.00001441955	0.00002882243	0.002690214552	0.0000000002005759405	0.000000000236769011	0.004276324926	0.0000000002383614961
0.00001443622	0.0000288391	0.002693324627	0.0000000002008078202	0.0000000002370427324	0.00427879822	0.000000000238499357
0.00001445289	0.00002888911	0.002696434701	0.0000000002010396999	0.0000000002373164538	0.004286218101	0.0000000002389129397
0.00001446956	0.00002890578	0.002699544776	0.0000000002012715796	0.0000000002375901752	0.004288691395	0.0000000002390508006
0.00001448623	0.00002893912	0.002702654851	0.0000000002015034593	0.0000000002378638966	0.004293637982	0.0000000002393265224
0.0000145029	0.00002897246	0.002705764925	0.000000000201735339	0.000000000238137618	0.00429858457	0.0000000002396022442
0.00001451957	0.00002898913	0.002708875	0.0000000002019672187	0.0000000002384113394	0.004301057864	0.0000000002397401051
0.00001453624	0.00002903914	0.002711985075	0.0000000002021990984	0.0000000002386850608	0.004308477745	0.0000000002401536878
0.00001455291	0.00002905581	0.002715095149	0.0000000002024309781	0.0000000002389587822	0.004310951039	0.0000000002402915487
0.00001456958	0.00002910582	0.002718205224	0.0000000002026628578	0.0000000002392325036	0.00431837092	0.0000000002407051314
0.00001458625	0.00002913916	0.002721315299	0.0000000002028947375	0.000000000239506225	0.004323317507	0.0000000002409808532
0.00001460292	0.0000291725	0.002724425373	0.0000000002031266172	0.0000000002397799464	0.004328264095	0.000000000241256575
0.00001461959	0.00002920584	0.002727535448	0.0000000002033584969	0.0000000002400536678	0.004333210682	0.0000000002415322968
0.00001463626	0.00002925585	0.002730645522	0.0000000002035903766	0.0000000002403273892	0.004340630564	0.0000000002419458795
0.00001465293	0.00002927252	0.002733755597	0.0000000002038222563	0.0000000002406011106	0.004343103858	0.0000000002420837404
0.0000146696	0.00002932253	0.002736865672	0.000000000204054136	0.000000000240874832	0.004350523739	0.0000000002424973231

0.00001468627	0.00002935587	0.002739975746	0.0000000002042860157	0.0000000002411485534	0.004355470326	0.0000000002427730449
0.00001470294	0.00002938921	0.002743085821	0.0000000002045178954	0.0000000002414222748	0.004360416914	0.0000000002430487667
0.00001471961	0.00002940588	0.002746195896	0.0000000002047497751	0.0000000002416959962	0.004362890208	0.0000000002431866276
0.00001473628	0.00002945589	0.00274930597	0.0000000002049816548	0.0000000002419697176	0.004370310089	0.0000000002436002103
0.00001475295	0.00002947256	0.002752416045	0.0000000002052135345	0.000000000242243439	0.004372783383	0.0000000002437380712
0.00001476962	0.00002952257	0.002755526119	0.0000000002054454142	0.0000000002425171604	0.004380203264	0.0000000002441516539
0.00001478629	0.00002953924	0.002758636194	0.0000000002056772939	0.0000000002427908818	0.004382676558	0.0000000002442895148
0.00001480296	0.00002958925	0.002761746269	0.0000000002059091736	0.0000000002430646032	0.004390096439	0.0000000002447030975
0.00001481963	0.00002962259	0.002764856343	0.0000000002061410533	0.0000000002433383246	0.004395043027	0.0000000002449788193
0.0000148363	0.00002965593	0.002767966418	0.000000000206372933	0.000000000243612046	0.004399989614	0.0000000002452545411
0.00001485297	0.00002968927	0.002771076493	0.0000000002066048127	0.0000000002438857674	0.004404936202	0.0000000002455302629
0.00001486964	0.00002972261	0.002774186567	0.0000000002068366924	0.0000000002441594888	0.004409882789	0.0000000002458059847
0.00001488631	0.00002975595	0.002777296642	0.0000000002070685721	0.0000000002444332102	0.004414829377	0.0000000002460817065
0.00001490298	0.00002977262	0.002780406716	0.0000000002073004518	0.0000000002447069316	0.004417302671	0.0000000002462195674
0.00001491965	0.00002980596	0.002783516791	0.0000000002075323315	0.000000000244980653	0.004422249258	0.0000000002464952892
0.00001493632	0.0000298393	0.002786626866	0.0000000002077642112	0.0000000002452543744	0.004427195846	0.000000000246771011
0.00001495299	0.00002985597	0.00278973694	0.0000000002079960909	0.0000000002455280958	0.004429669139	0.0000000002469088719
0.00001496966	0.00002988931	0.002792847015	0.0000000002082279706	0.0000000002458018172	0.004434615727	0.0000000002471845937
0.00001498633	0.00002993932	0.00279595709	0.0000000002084598503	0.0000000002460755386	0.004442035608	0.0000000002475981764
0.000015003	0.00002997266	0.002799067164	0.00000000020869173	0.00000000024634926	0.004446982196	0.0000000002478738982
0.00001501967	0.00003002267	0.002802177239	0.0000000002089236097	0.0000000002466229814	0.004454402077	0.0000000002482874809
0.00001503634	0.00003005601	0.002805287313	0.0000000002091554894	0.0000000002468967028	0.004459348665	0.0000000002485632027
0.00001505301	0.00003007268	0.002808397388	0.0000000002093873691	0.0000000002471704242	0.004461821958	0.0000000002487010636
0.00001506968	0.00003010602	0.002811507463	0.0000000002096192488	0.0000000002474441456	0.004466768546	0.0000000002489767854
0.00001508635	0.00003015603	0.002814617537	0.0000000002098511285	0.000000000247717867	0.004474188427	0.0000000002493903681
0.00001510302	0.0000301727	0.002817727612	0.0000000002100830082	0.0000000002479915884	0.004476661721	0.000000000249528229
0.00001511969	0.00003018937	0.002820837687	0.0000000002103148879	0.0000000002482653098	0.004479135015	0.0000000002496660899
0.00001513636	0.00003020604	0.002823947761	0.0000000002105467676	0.0000000002485390312	0.004481608309	0.0000000002498039508
0.00001515303	0.00003025605	0.002827057836	0.0000000002107786473	0.0000000002488127526	0.00448902819	0.0000000002502175335
0.0000151697	0.00003030606	0.00283016791	0.000000000211010527	0.000000000249086474	0.004496448071	0.0000000002506311162
0.00001518637	0.00003032273	0.002833277985	0.0000000002112424067	0.0000000002493601954	0.004498921365	0.0000000002507689771
0.00001520304	0.00003037274	0.00283638806	0.0000000002114742864	0.0000000002496339168	0.004506341246	0.0000000002511825598
0.00001521971	0.00003042275	0.002839498134	0.0000000002117061661	0.0000000002499076382	0.004513761128	0.0000000002515961425
0.00001523638	0.00003045609	0.002842608209	0.0000000002119380458	0.0000000002501813596	0.004518707715	0.0000000002518718643
0.00001525305	0.00003048943	0.002845718284	0.0000000002121699255	0.000000000250455081	0.004523654303	0.0000000002521475861
0.00001526972	0.00003052277	0.002848828358	0.0000000002124018052	0.0000000002507288024	0.00452860089	0.0000000002524233079
0.00001528639	0.00003055611	0.002851938433	0.0000000002126336849	0.0000000002510025238	0.004533547478	0.0000000002526990297
0.00001530306	0.00003058945	0.002855048507	0.0000000002128655646	0.0000000002512762452	0.004538494065	0.0000000002529747515
0.00001531973	0.00003062279	0.002858158582	0.0000000002130974443	0.0000000002515499666	0.004543440653	0.0000000002532504733
0.0000153364	0.00003065613	0.002861268657	0.000000000213329324	0.000000000251823688	0.00454838724	0.0000000002535261951

0.00001535307	0.00003068947	0.002864378731	0.0000000002135612037	0.0000000002520974094	0.004553333828	0.0000000002538019169
0.00001536974	0.00003070614	0.002867488806	0.0000000002137930834	0.0000000002523711308	0.004555807122	0.0000000002539397778
0.00001538641	0.00003073948	0.002870598881	0.0000000002140249631	0.0000000002526448522	0.004560753709	0.0000000002542154996
0.00001540308	0.00003077282	0.002873708955	0.0000000002142568428	0.0000000002529185736	0.004565700297	0.0000000002544912214
0.00001541975	0.00003082283	0.00287681903	0.0000000002144887225	0.000000000253192295	0.004573120178	0.0000000002549048041
0.00001543642	0.0000308395	0.002879929104	0.0000000002147206022	0.0000000002534660164	0.004575593472	0.000000000255042665
0.00001545309	0.00003085617	0.002883039179	0.0000000002149524819	0.0000000002537397378	0.004578066766	0.0000000002551805259
0.00001546976	0.00003090618	0.002886149254	0.0000000002151843616	0.0000000002540134592	0.004585486647	0.0000000002555941086
0.00001548643	0.00003095619	0.002889259328	0.0000000002154162413	0.0000000002542871806	0.004592906528	0.0000000002560076913
0.0000155031	0.00003098953	0.002892369403	0.000000000215648121	0.000000000254560902	0.004597853116	0.0000000002562834131
0.00001551977	0.00003102287	0.002895479478	0.0000000002158800007	0.0000000002548346234	0.004602799703	0.0000000002565591349
0.00001553644	0.00003103954	0.002898589552	0.0000000002161118804	0.0000000002551083448	0.004605272997	0.0000000002566969958
0.00001555311	0.00003107288	0.002901699627	0.0000000002163437601	0.0000000002553820662	0.004610219585	0.0000000002569727176
0.00001556978	0.00003108955	0.002904809701	0.0000000002165756398	0.0000000002556557876	0.004612692878	0.0000000002571105785
0.00001558645	0.00003113956	0.002907919776	0.0000000002168075195	0.000000000255929509	0.00462011276	0.0000000002575241612
0.00001560312	0.00003115623	0.002911029851	0.0000000002170393992	0.0000000002562032304	0.004622586053	0.0000000002576620221
0.00001561979	0.00003120624	0.002914139925	0.0000000002172712789	0.0000000002564769518	0.004630005935	0.0000000002580756048
0.00001563646	0.00003123958	0.00291725	0.0000000002175031586	0.0000000002567506732	0.004634952522	0.0000000002583513266
0.00001565313	0.00003127292	0.002920360075	0.0000000002177350383	0.0000000002570243946	0.00463989911	0.0000000002586270484
0.0000156698	0.00003132293	0.002923470149	0.000000000217966918	0.000000000257298116	0.004647318991	0.0000000002590406311
0.00001568647	0.0000313396	0.002926580224	0.0000000002181987977	0.0000000002575718374	0.004649792285	0.000000000259178492
0.00001570314	0.00003135627	0.002929690299	0.0000000002184306774	0.0000000002578455588	0.004652265579	0.0000000002593163529
0.00001571981	0.00003138961	0.002932800373	0.0000000002186625571	0.0000000002581192802	0.004657212166	0.0000000002595920747
0.00001573648	0.00003143962	0.002935910448	0.0000000002188944368	0.0000000002583930016	0.004664632047	0.0000000002600056574
0.00001575315	0.00003147296	0.002939020522	0.0000000002191263165	0.000000000258666723	0.004669578635	0.0000000002602813792
0.00001576982	0.0000315063	0.002942130597	0.0000000002193581962	0.0000000002589404444	0.004674525223	0.000000000260557101
0.00001578649	0.00003153964	0.002945240672	0.0000000002195900759	0.0000000002592141658	0.00467947181	0.0000000002608328228
0.00001580316	0.00003158965	0.002948350746	0.0000000002198219556	0.0000000002594878872	0.004686891691	0.0000000002612464055
0.00001581983	0.00003160632	0.002951460821	0.0000000002200538353	0.0000000002597616086	0.004689364985	0.0000000002613842664
0.0000158365	0.00003165633	0.002954570896	0.000000000220285715	0.00000000026003533	0.004696784866	0.0000000002617978491
0.00001585317	0.000031673	0.00295768097	0.0000000002205175947	0.0000000002603090514	0.00469925816	0.00000000026193571
0.00001586984	0.00003170634	0.002960791045	0.0000000002207494744	0.0000000002605827728	0.004704204748	0.0000000002622114318
0.00001588651	0.00003173968	0.002963901119	0.0000000002209813541	0.0000000002608564942	0.004709151335	0.0000000002624871536
0.00001590318	0.00003178969	0.002967011194	0.0000000002212132338	0.0000000002611302156	0.004716571217	0.0000000002629007363
0.00001591985	0.00003180636	0.002970121269	0.0000000002214451135	0.000000000261403937	0.00471904451	0.0000000002630385972
0.00001593652	0.00003185637	0.002973231343	0.0000000002216769932	0.0000000002616776584	0.004726464392	0.0000000002634521799
0.00001595319	0.00003187304	0.002976341418	0.0000000002219088729	0.0000000002619513798	0.004728937685	0.0000000002635900408
0.00001596986	0.00003192305	0.002979451493	0.0000000002221407526	0.0000000002622251012	0.004736357567	0.0000000002640036235
0.00001598653	0.00003193972	0.002982561567	0.0000000002223726323	0.0000000002624988226	0.004738830861	0.0000000002641414844
0.0000160032	0.00003197306	0.002985671642	0.000000000222604512	0.000000000262772544	0.004743777448	0.0000000002644172062

0.00001601987	0.0000320064	0.002988781716	0.0000000002228363917	0.0000000002630462654	0.004748724036	0.000000000264692928
0.00001603654	0.00003203974	0.002991891791	0.0000000002230682714	0.0000000002633199868	0.004753670623	0.0000000002649686498
0.00001605321	0.00003207308	0.002995001866	0.0000000002233001511	0.0000000002635937082	0.004758617211	0.0000000002652443716
0.00001606988	0.00003210642	0.00299811194	0.0000000002235320308	0.0000000002638674296	0.004763563798	0.0000000002655200934
0.00001608655	0.00003212309	0.003001222015	0.0000000002237639105	0.000000000264141151	0.004766037092	0.0000000002656579543
0.00001610322	0.00003215643	0.00300433209	0.0000000002239957902	0.0000000002644148724	0.00477098368	0.0000000002659336761
0.00001611989	0.00003220644	0.003007442164	0.0000000002242276699	0.0000000002646885938	0.004778403561	0.0000000002663472588
0.00001613656	0.00003225645	0.003010552239	0.0000000002244595496	0.0000000002649623152	0.004785823442	0.0000000002667608415
0.00001615323	0.00003227312	0.003013662313	0.0000000002246914293	0.0000000002652360366	0.004788296736	0.0000000002668987024
0.0000161699	0.00003230646	0.003016772388	0.000000000224923309	0.000000000265509758	0.004793243323	0.0000000002671744242
0.00001618657	0.0000323398	0.003019882463	0.0000000002251551887	0.0000000002657834794	0.004798189911	0.000000000267450146
0.00001620324	0.00003235647	0.003022992537	0.0000000002253870684	0.0000000002660572008	0.004800663205	0.0000000002675880069
0.00001621991	0.00003238981	0.003026102612	0.0000000002256189481	0.0000000002663309222	0.004805609792	0.0000000002678637287
0.00001623658	0.00003243982	0.003029212687	0.0000000002258508278	0.0000000002666046436	0.004813029674	0.0000000002682773114
0.00001625325	0.00003248983	0.003032322761	0.0000000002260827075	0.000000000266878365	0.004820449555	0.0000000002686908941
0.00001626992	0.00003252317	0.003035432836	0.0000000002263145872	0.0000000002671520864	0.004825396142	0.0000000002689666159
0.00001628659	0.00003255651	0.00303854291	0.0000000002265464669	0.0000000002674258078	0.00483034273	0.0000000002692423377
0.00001630326	0.00003258985	0.003041652985	0.0000000002267783466	0.0000000002676995292	0.004835289318	0.0000000002695180595
0.00001631993	0.00003260652	0.00304476306	0.0000000002270102263	0.0000000002679732506	0.004837762611	0.0000000002696559204
0.0000163366	0.00003265653	0.003047873134	0.000000000227242106	0.000000000268246972	0.004845182493	0.0000000002700695031
0.00001635327	0.0000326732	0.003050983209	0.0000000002274739857	0.0000000002685206934	0.004847655786	0.000000000270207364
0.00001636994	0.00003270654	0.003054093284	0.0000000002277058654	0.0000000002687944148	0.004852602374	0.0000000002704830858
0.00001638661	0.00003273988	0.003057203358	0.0000000002279377451	0.0000000002690681362	0.004857548961	0.0000000002707588076
0.00001640328	0.00003278989	0.003060313433	0.0000000002281696248	0.0000000002693418576	0.004864968843	0.0000000002711723903
0.00001641995	0.00003282323	0.003063423507	0.0000000002284015045	0.000000000269615579	0.00486991543	0.0000000002714481121
0.00001643662	0.0000328399	0.003066533582	0.0000000002286333842	0.0000000002698893004	0.004872388724	0.000000000271585973
0.00001645329	0.00003288991	0.003069643657	0.0000000002288652639	0.0000000002701630218	0.004879808605	0.0000000002719995557
0.00001646996	0.00003292325	0.003072753731	0.0000000002290971436	0.0000000002704367432	0.004884755193	0.0000000002722752775
0.00001648663	0.00003293992	0.003075863806	0.0000000002293290233	0.0000000002707104646	0.004887228487	0.0000000002724131384
0.0000165033	0.00003297326	0.003078973881	0.000000000229560903	0.000000000270984186	0.004892175074	0.0000000002726888602
0.00001651997	0.00003302327	0.003082083955	0.0000000002297927827	0.0000000002712579074	0.004899594955	0.0000000002731024429
0.00001653664	0.00003305661	0.00308519403	0.0000000002300246624	0.0000000002715316288	0.004904541543	0.0000000002733781647
0.00001655331	0.00003307328	0.003088304104	0.0000000002302565421	0.0000000002718053502	0.004907014837	0.0000000002735160256
0.00001656998	0.00003312329	0.003091414179	0.0000000002304884218	0.0000000002720790716	0.004914434718	0.0000000002739296083
0.00001658665	0.00003315663	0.003094524254	0.0000000002307203015	0.000000000272352793	0.004919381306	0.0000000002742053301
0.00001660332	0.0000331733	0.003097634328	0.0000000002309521812	0.0000000002726265144	0.004921854599	0.000000000274343191
0.00001661999	0.00003322331	0.003100744403	0.0000000002311840609	0.0000000002729002358	0.004929274481	0.0000000002747567737
0.00001663666	0.00003325665	0.003103854478	0.0000000002314159406	0.0000000002731739572	0.004934221068	0.0000000002750324955
0.00001665333	0.00003328999	0.003106964552	0.0000000002316478203	0.0000000002734476786	0.004939167656	0.0000000002753082173
0.00001667	0.00003332333	0.003110074627	0.0000000002318797	0.0000000002737214	0.004944114243	0.0000000002755839391

0.00001668667	0.00003335667	0.003113184701	0.0000000002321115797	0.0000000002739951214	0.004949060831	0.0000000002758596609
0.00001670334	0.00003339001	0.003116294776	0.0000000002323434594	0.0000000002742688428	0.004954007418	0.0000000002761353827
0.00001672001	0.00003340668	0.003119404851	0.0000000002325753391	0.0000000002745425642	0.004956480712	0.0000000002762732436
0.00001673668	0.00003344002	0.003122514925	0.0000000002328072188	0.0000000002748162856	0.0049614273	0.0000000002765489654
0.00001675335	0.00003349003	0.003125625	0.0000000002330390985	0.000000000275090007	0.004968847181	0.0000000002769625481
0.00001677002	0.00003352337	0.003128735075	0.0000000002332709782	0.0000000002753637284	0.004973793769	0.0000000002772382699
0.00001678669	0.00003354004	0.003131845149	0.0000000002335028579	0.0000000002756374498	0.004976267062	0.0000000002773761308
0.00001680336	0.00003355671	0.003134955224	0.0000000002337347376	0.0000000002759111712	0.004978740356	0.0000000002775139917
0.00001682003	0.00003357338	0.003138065299	0.0000000002339666173	0.0000000002761848926	0.00498121365	0.0000000002776518526
0.0000168367	0.00003360672	0.003141175373	0.000000000234198497	0.000000000276458614	0.004986160237	0.0000000002779275744
0.00001685337	0.00003364006	0.003144285448	0.0000000002344303767	0.0000000002767323354	0.004991106825	0.0000000002782032962
0.00001687004	0.00003365673	0.003147395522	0.0000000002346622564	0.0000000002770060568	0.004993580119	0.0000000002783411571
0.00001688671	0.0000336734	0.003150505597	0.0000000002348941361	0.0000000002772797782	0.004996053412	0.000000000278479018
0.00001690338	0.00003370674	0.003153615672	0.0000000002351260158	0.0000000002775534996	0.005001	0.0000000002787547398
0.00001692005	0.00003372341	0.003156725746	0.0000000002353578955	0.000000000277827221	0.005003473294	0.0000000002788926007
0.00001693672	0.00003375675	0.003159835821	0.0000000002355897752	0.0000000002781009424	0.005008419881	0.0000000002791683225
0.00001695339	0.00003377342	0.003162945896	0.0000000002358216549	0.0000000002783746638	0.005010893175	0.0000000002793061834
0.00001697006	0.00003379009	0.00316605597	0.0000000002360535346	0.0000000002786483852	0.005013366469	0.0000000002794440443
0.00001698673	0.00003380676	0.003169166045	0.0000000002362854143	0.0000000002789221066	0.005015839763	0.0000000002795819052
0.0000170034	0.0000338401	0.003172276119	0.000000000236517294	0.000000000279195828	0.00502078635	0.000000000279857627
0.00001702007	0.00003387344	0.003175386194	0.0000000002367491737	0.0000000002794695494	0.005025732938	0.0000000002801333488
0.00001703674	0.00003389011	0.003178496269	0.0000000002369810534	0.0000000002797432708	0.005028206231	0.0000000002802712097
0.00001705341	0.00003392345	0.003181606343	0.0000000002372129331	0.0000000002800169922	0.005033152819	0.0000000002805469315
0.00001707008	0.00003399013	0.003184716418	0.0000000002374448128	0.0000000002802907136	0.005043045994	0.0000000002810983751
0.00001708675	0.0000340068	0.003187826493	0.0000000002376766925	0.000000000280564435	0.005045519288	0.000000000281236236
0.00001710342	0.00003404014	0.003190936567	0.0000000002379085722	0.0000000002808381564	0.005050465875	0.0000000002815119578
0.00001712009	0.00003407348	0.003194046642	0.0000000002381404519	0.0000000002811118778	0.005055412463	0.0000000002817876796
0.00001713676	0.00003409015	0.003197156716	0.0000000002383723316	0.0000000002813855992	0.005057885757	0.0000000002819255405
0.00001715343	0.00003412349	0.003200266791	0.0000000002386042113	0.0000000002816593206	0.005062832344	0.0000000002822012623
0.0000171701	0.00003414016	0.003203376866	0.000000000238836091	0.000000000281933042	0.005065305638	0.0000000002823391232
0.00001718677	0.00003419017	0.00320648694	0.0000000002390679707	0.0000000002822067634	0.005072725519	0.0000000002827527059
0.00001720344	0.00003422351	0.003209597015	0.0000000002392998504	0.0000000002824804848	0.005077672107	0.0000000002830284277
0.00001722011	0.00003424018	0.00321270709	0.0000000002395317301	0.0000000002827542062	0.005080145401	0.0000000002831662886
0.00001723678	0.00003427352	0.003215817164	0.0000000002397636098	0.0000000002830279276	0.005085091988	0.0000000002834420104
0.00001725345	0.00003430686	0.003218927239	0.0000000002399954895	0.000000000283301649	0.005090038576	0.0000000002837177322
0.00001727012	0.00003435687	0.003222037313	0.0000000002402273692	0.0000000002835753704	0.005097458457	0.0000000002841313149
0.00001728679	0.00003440688	0.003225147388	0.0000000002404592489	0.0000000002838490918	0.005104878338	0.0000000002845448976
0.00001730346	0.00003442355	0.003228257463	0.0000000002406911286	0.0000000002841228132	0.005107351632	0.0000000002846827585
0.00001732013	0.00003444022	0.003231367537	0.0000000002409230083	0.0000000002843965346	0.005109824926	0.0000000002848206194
0.0000173368	0.00003447356	0.003234477612	0.000000000241154888	0.000000000284670256	0.005114771513	0.0000000002850963412

0.00001735347	0.0000345069	0.003237587687	0.0000000002413867677	0.0000000002849439774	0.005119718101	0.000000000285372063
0.00001737014	0.00003452357	0.003240697761	0.0000000002416186474	0.0000000002852176988	0.005122191395	0.0000000002855099239
0.00001738681	0.00003457358	0.003243807836	0.0000000002418505271	0.0000000002854914202	0.005129611276	0.0000000002859235066
0.00001740348	0.00003460692	0.00324691791	0.0000000002420824068	0.0000000002857651416	0.005134557864	0.0000000002861992284
0.00001742015	0.00003462359	0.003250027985	0.0000000002423142865	0.000000000286038863	0.005137031157	0.0000000002863370893
0.00001743682	0.0000346736	0.00325313806	0.0000000002425461662	0.0000000002863125844	0.005144451039	0.000000000286750672
0.00001745349	0.00003469027	0.003256248134	0.0000000002427780459	0.0000000002865863058	0.005146924332	0.0000000002868885329
0.00001747016	0.00003474028	0.003259358209	0.0000000002430099256	0.0000000002868600272	0.005154344214	0.0000000002873021156
0.00001748683	0.00003477362	0.003262468284	0.0000000002432418053	0.0000000002871337486	0.005159290801	0.0000000002875778374
0.0000175035	0.00003480696	0.003265578358	0.000000000243473685	0.00000000028740747	0.005164237389	0.0000000002878535592
0.00001752017	0.0000348403	0.003268688433	0.0000000002437055647	0.0000000002876811914	0.005169183976	0.000000000288129281
0.00001753684	0.00003489031	0.003271798507	0.0000000002439374444	0.0000000002879549128	0.005176603858	0.0000000002885428637
0.00001755351	0.00003492365	0.003274908582	0.0000000002441693241	0.0000000002882286342	0.005181550445	0.0000000002888185855
0.00001757018	0.00003495699	0.003278018657	0.0000000002444012038	0.0000000002885023556	0.005186497033	0.0000000002890943073
0.00001758685	0.00003499033	0.003281128731	0.0000000002446330835	0.000000000288776077	0.00519144362	0.0000000002893700291
0.00001760352	0.00003352337	0.003284238806	0.0000000002448649632	0.0000000002890497984	0.004973793769	0.0000000002772382699
0.00001762019	0.00003355671	0.003287348881	0.0000000002450968429	0.0000000002893235198	0.004978740356	0.0000000002775139917
0.00001763686	0.00003360672	0.003290458955	0.0000000002453287226	0.0000000002895972412	0.004986160237	0.0000000002779275744
0.00001765353	0.00003365673	0.00329356903	0.0000000002455606023	0.0000000002898709626	0.004993580119	0.0000000002783411571
0.0000176702	0.00003369007	0.003296679104	0.000000000245792482	0.000000000290144684	0.004998526706	0.0000000002786168789
0.00001768687	0.00003375675	0.003299789179	0.0000000002460243617	0.0000000002904184054	0.005008419881	0.0000000002791683225
0.00001770354	0.00003380676	0.003302899254	0.0000000002462562414	0.0000000002906921268	0.005015839763	0.0000000002795819052
0.00001772021	0.00003385677	0.003306009328	0.0000000002464881211	0.0000000002909658482	0.005023259644	0.0000000002799954879
0.00001773688	0.00003394012	0.003309119403	0.0000000002467200008	0.0000000002912395696	0.005035626113	0.0000000002806847924
0.00001775355	0.00003389011	0.003312229478	0.0000000002469518805	0.000000000291513291	0.005028206231	0.0000000002802712097
0.00001777022	0.00003395679	0.003315339552	0.0000000002471837602	0.0000000002917870124	0.005038099407	0.0000000002808226533
0.00001778689	0.00003407348	0.003318449627	0.0000000002474156399	0.0000000002920607338	0.005055412463	0.0000000002817876796
0.00001780356	0.00003414016	0.003321559701	0.0000000002476475196	0.0000000002923344552	0.005065305638	0.0000000002823391232
0.00001782023	0.00003422351	0.003324669776	0.0000000002478793993	0.0000000002926081766	0.005077672107	0.0000000002830284277
0.0000178369	0.00003429019	0.003327779851	0.000000000248111279	0.000000000292881898	0.005087565282	0.0000000002835798713
0.00001785357	0.00003432353	0.003330889925	0.0000000002483431587	0.0000000002931556194	0.005092511869	0.0000000002838555931
0.00001787024	0.00003440688	0.003334	0.0000000002485750384	0.0000000002934293408	0.005104878338	0.0000000002845448976
0.00001788691	0.00003437354	0.003337110075	0.0000000002488069181	0.0000000002937030622	0.005099931751	0.0000000002842691758
0.00001790358	0.00003439021	0.003340220149	0.0000000002490387978	0.0000000002939767836	0.005102405045	0.0000000002844070367
0.00001792025	0.00003454024	0.003343330224	0.0000000002492706775	0.000000000294250505	0.005124664688	0.0000000002856477848
0.00001793692	0.00003449023	0.003346440299	0.0000000002495025572	0.0000000002945242264	0.005117244807	0.0000000002852342021
0.00001795359	0.00003444022	0.003349550373	0.0000000002497344369	0.0000000002947979478	0.005109824926	0.0000000002848206194
0.00001797026	0.00003455691	0.003352660448	0.0000000002499663166	0.0000000002950716692	0.005127137982	0.0000000002857856457
0.00001798693	0.00003459025	0.003355770522	0.0000000002501981963	0.0000000002953453906	0.00513208457	0.0000000002860613675
0.0000180036	0.00003445689	0.003358880597	0.000000000250430076	0.000000000295619112	0.00511229822	0.0000000002849584803

0.00001802027	0.0000345069	0.003361990672	0.0000000002506619557	0.0000000002958928334	0.005119718101	0.000000000285372063
0.00001803694	0.00003452357	0.003365100746	0.0000000002508938354	0.0000000002961665548	0.005122191395	0.0000000002855099239
0.00001805361	0.00003447356	0.003368210821	0.0000000002511257151	0.0000000002964402762	0.005114771513	0.0000000002850963412
0.00001807028	0.00003460692	0.003371320896	0.0000000002513575948	0.0000000002967139976	0.005134557864	0.0000000002861992284
0.00001808695	0.00003457358	0.00337443097	0.0000000002515894745	0.000000000296987719	0.005129611276	0.0000000002859235066
0.00001810362	0.00003442355	0.003377541045	0.0000000002518213542	0.0000000002972614404	0.005107351632	0.0000000002846827585
0.00001812029	0.00003462359	0.003380651119	0.0000000002520532339	0.0000000002975351618	0.005137031157	0.0000000002863370893
0.00001813696	0.00003449023	0.003383761194	0.0000000002522851136	0.0000000002978088832	0.005117244807	0.0000000002852342021
0.00001815363	0.00003464026	0.003386871269	0.0000000002525169933	0.0000000002980826046	0.005139504451	0.0000000002864749502
0.0000181703	0.00003465693	0.003389981343	0.000000000252748873	0.000000000298356326	0.005141977745	0.0000000002866128111
0.00001818697	0.00003469027	0.003393091418	0.0000000002529807527	0.0000000002986300474	0.005146924332	0.0000000002868885329
0.00001820364	0.0000346736	0.003396201493	0.0000000002532126324	0.0000000002989037688	0.005144451039	0.000000000286750672
0.00001822031	0.00003477362	0.003399311567	0.0000000002534445121	0.0000000002991774902	0.005159290801	0.0000000002875778374
0.00001823698	0.00003472361	0.003402421642	0.0000000002536763918	0.0000000002994512116	0.00515187092	0.0000000002871642547
0.00001825365	0.00003470694	0.003405531716	0.0000000002539082715	0.000000000299724933	0.005149397626	0.0000000002870263938
0.00001827032	0.00003479029	0.003408641791	0.0000000002541401512	0.0000000002999986544	0.005161764095	0.0000000002877156983
0.00001828699	0.00003485697	0.003411751866	0.0000000002543720309	0.0000000003002723758	0.00517165727	0.0000000002882671419
0.00001830366	0.00003482363	0.00341486194	0.0000000002546039106	0.0000000003005460972	0.005166710682	0.0000000002879914201
0.00001832033	0.00003474028	0.003417972015	0.0000000002548357903	0.0000000003008198186	0.005154344214	0.0000000002873021156
0.000018337	0.00003490698	0.00342108209	0.00000000025506767	0.00000000030109354	0.005179077151	0.0000000002886807246
0.0000185037	0.0000348403	0.003452182836	0.000000000257386467	0.000000000303830754	0.005169183976	0.000000000288129281
0.0000186704	0.0000351737	0.003483283582	0.000000000259705264	0.000000000306567968	0.005218649852	0.000000000290886499
0.0000188371	0.0000355071	0.003514384328	0.000000000262024061	0.000000000309305182	0.005268115727	0.000000000293643717
0.0000190038	0.000035007	0.003545485075	0.000000000264342858	0.000000000312042396	0.005193916914	0.00000000028950789
0.0000191705	0.0000360072	0.003576585821	0.000000000266661655	0.00000000031477961	0.00534231454	0.000000000297779544
0.0000193372	0.0000353404	0.003607686567	0.000000000268980452	0.000000000317516824	0.005243382789	0.000000000292265108
0.0000195039	0.0000363406	0.003638787313	0.000000000271299249	0.000000000320254038	0.005391780415	0.000000000300536762
0.0000196706	0.0000365073	0.00366988806	0.000000000273618046	0.000000000322991252	0.005416513353	0.000000000301915371
0.0000198373	0.0000361739	0.003700988806	0.000000000275936843	0.000000000325728466	0.005367047478	0.000000000299158153
0.000020004	0.000036674	0.003732089552	0.00000000027825564	0.00000000032846568	0.005441246291	0.00000000030329398
0.0000201707	0.0000368407	0.003763190299	0.000000000280574437	0.000000000331202894	0.005465979228	0.000000000304672589
0.0000203374	0.0000371741	0.003794291045	0.000000000282893234	0.000000000333940108	0.005515445104	0.000000000307429807
0.0000205041	0.0000373408	0.003825391791	0.000000000285212031	0.000000000336677322	0.005540178042	0.000000000308808416
0.0000206708	0.0000375075	0.003856492537	0.000000000287530828	0.000000000339414536	0.005564910979	0.000000000310187025
0.0000208375	0.0000380076	0.003887593284	0.000000000289849625	0.00000000034215175	0.005639109792	0.000000000314322852
0.0000210042	0.0000378409	0.00391869403	0.000000000292168422	0.000000000344888964	0.005614376855	0.000000000312944243
0.0000211709	0.0000385077	0.003949794776	0.000000000294487219	0.000000000347626178	0.005713308605	0.000000000318458679
0.0000213376	0.0000381743	0.003980895522	0.000000000296806016	0.000000000350363392	0.00566384273	0.000000000315701461
0.0000215043	0.000038341	0.004011996269	0.000000000299124813	0.000000000353100606	0.005688575668	0.00000000031708007
0.000021671	0.0000393412	0.004043097015	0.00000000030144361	0.00000000035583782	0.005836973294	0.000000000325351724

0.0000218377	0.0000395079	0.004074197761	0.000000000303762407	0.000000000358575034	0.005861706231	0.000000000326730333
0.0000220044	0.0000396746	0.004105298507	0.000000000306081204	0.000000000361312248	0.005886439169	0.000000000328108942
0.0000223378	0.0000401747	0.0041675	0.000000000310718798	0.000000000366786676	0.005960637982	0.000000000332244769
0.0000225045	0.0000406748	0.004198600746	0.000000000313037595	0.00000000036952389	0.006034836795	0.000000000336380596
0.0000226712	0.000040008	0.004229701493	0.000000000315356392	0.000000000372261104	0.005935905045	0.00000000033086616
0.0000228379	0.0000398413	0.004260802239	0.000000000317675189	0.000000000374998318	0.005911172107	0.000000000329487551
0.0000230046	0.0000411749	0.004291902985	0.000000000319993986	0.000000000377735532	0.006109035608	0.000000000340516423
0.0000231713	0.0000408415	0.004323003731	0.000000000322312783	0.000000000380472746	0.006059569733	0.000000000337759205
0.000023338	0.0000415083	0.004354104478	0.00000000032463158	0.00000000038320996	0.006158501484	0.000000000343273641
0.0000235047	0.0000418417	0.004385205224	0.000000000326950377	0.000000000385947174	0.006207967359	0.000000000346030859
0.0000236714	0.0000421751	0.00441630597	0.000000000329269174	0.000000000388684388	0.006257433234	0.000000000348788077
0.0000238381	0.0000431753	0.004447406716	0.000000000331587971	0.000000000391421602	0.006405830861	0.000000000357059731
0.0000240048	0.000041675	0.004478507463	0.000000000333906768	0.000000000394158816	0.006183234421	0.00000000034465225
0.0000241715	0.0000425085	0.004509608209	0.000000000336225565	0.00000000039689603	0.00630689911	0.000000000351545295
0.0000243382	0.0000440088	0.004540708955	0.000000000338544362	0.000000000399633244	0.006529495549	0.000000000363952776
0.0000245049	0.0000446756	0.004571809701	0.000000000340863159	0.000000000402370458	0.0066284273	0.000000000369467212
0.0000246716	0.0000441755	0.004602910448	0.000000000343181956	0.000000000405107672	0.006554228487	0.000000000365331385
0.0000248383	0.0000451757	0.004634011194	0.000000000345500753	0.000000000407844886	0.006702626113	0.000000000373603039
0.000025005	0.0000456758	0.00466511194	0.00000000034781955	0.0000000004105821	0.006776824926	0.000000000377738866
0.0000251717	0.0000461759	0.004696212687	0.000000000350138347	0.000000000413319314	0.006851023739	0.000000000381874693
0.0000253384	0.0000465093	0.004727313433	0.000000000352457144	0.000000000416056528	0.006900489614	0.000000000384631911
0.0000255051	0.0000471761	0.004758414179	0.000000000354775941	0.000000000418793742	0.006999421365	0.000000000390146347
0.0000256718	0.0000468427	0.004789514925	0.000000000357094738	0.000000000421530956	0.00694995549	0.000000000387389129
0.0000258385	0.0000476762	0.004820615672	0.000000000359413535	0.00000000042426817	0.007073620178	0.000000000394282174
0.0000260052	0.0000481763	0.004851716418	0.000000000361732332	0.000000000427005384	0.007147818991	0.000000000398418001
0.0000261719	0.0000490098	0.004882817164	0.000000000364051129	0.000000000429742598	0.00727148368	0.000000000405311046
0.0000263386	0.0000485097	0.00491391791	0.000000000366369926	0.000000000432479812	0.007197284866	0.000000000401175219
0.0000265053	0.0000495099	0.004945018657	0.000000000368688723	0.000000000435217026	0.007345682493	0.000000000409446873
0.000026672	0.0000498433	0.004976119403	0.00000000037100752	0.00000000043795424	0.007395148368	0.000000000412204091
0.0000268387	0.0000491765	0.005007220149	0.000000000373326317	0.000000000440691454	0.007296216617	0.000000000406689655
0.0000270054	0.0000501767	0.005038320896	0.000000000375645114	0.000000000443428668	0.007444614243	0.000000000414961309
0.0000271721	0.0000508435	0.005069421642	0.000000000377963911	0.000000000446165882	0.007543545994	0.000000000420475745
0.0000273388	0.0000505101	0.005100522388	0.000000000380282708	0.000000000448903096	0.007494080119	0.000000000417718527
0.0000275055	0.0000506768	0.005131623134	0.000000000382601505	0.00000000045164031	0.007518813056	0.000000000419097136
0.0000276722	0.0000515103	0.005162723881	0.000000000384920302	0.000000000454377524	0.007642477745	0.000000000425990181
0.0000278389	0.0000511769	0.005193824627	0.000000000387239099	0.000000000457114738	0.007593011869	0.000000000423232963
0.0000280056	0.0000520104	0.005224925373	0.000000000389557896	0.000000000459851952	0.007716676558	0.000000000430126008
0.0000281723	0.0000518437	0.005256026119	0.000000000391876693	0.000000000462589166	0.00769194362	0.000000000428747399
0.000028339	0.0000528439	0.005287126866	0.00000000039419549	0.00000000046532638	0.007840341246	0.000000000437019053
0.0000285057	0.0000525105	0.005318227612	0.000000000396514287	0.000000000468063594	0.007790875371	0.000000000434261835

0.0000286724	0.0000531773	0.005349328358	0.000000000398833084	0.000000000470800808	0.007889807122	0.000000000439776271
0.0000288391	0.0000526772	0.005380429104	0.000000000401151881	0.000000000473538022	0.007815608309	0.000000000435640444
0.0000290058	0.0000540108	0.005411529851	0.000000000403470678	0.000000000476275236	0.00801347181	0.000000000446669316
0.0000291725	0.0000545109	0.005442630597	0.000000000405789475	0.00000000047901245	0.008087670623	0.000000000450805143
0.0000293392	0.0000535107	0.005473731343	0.000000000408108272	0.000000000481749664	0.007939272997	0.000000000442533489
0.0000295059	0.0000551777	0.00550483209	0.000000000410427069	0.000000000484486878	0.008186602374	0.000000000456319579
0.0000296726	0.0000548443	0.005535932836	0.000000000412745866	0.000000000487224092	0.008137136499	0.000000000453562361
0.0000298393	0.0000546776	0.005567033582	0.000000000415064663	0.000000000489961306	0.008112403561	0.000000000452183752
0.000030006	0.0000555111	0.005598134328	0.00000000041738346	0.00000000049269852	0.008236068249	0.000000000459076797
0.0000301727	0.0000561779	0.005629235075	0.000000000419702257	0.000000000495435734	0.008335	0.000000000464591233
0.0000303394	0.0000558445	0.005660335821	0.000000000422021054	0.000000000498172948	0.008285534125	0.000000000461834015
0.0000305061	0.000056678	0.005691436567	0.000000000424339851	0.000000000500910162	0.008409198813	0.00000000046872706
0.0000306728	0.0000565113	0.005722537313	0.000000000426658648	0.000000000503647376	0.008384465875	0.000000000467348451
0.0000308395	0.0000575115	0.00575363806	0.000000000428977445	0.00000000050638459	0.008532863501	0.000000000475620105
0.0000310062	0.0000571781	0.005784738806	0.000000000431296242	0.000000000509121804	0.008483397626	0.000000000472862887
0.0000311729	0.0000578449	0.005815839552	0.000000000433615039	0.000000000511859018	0.008582329377	0.000000000478377323
0.0000313396	0.0000585117	0.005846940299	0.000000000435933836	0.000000000514596232	0.008681261128	0.000000000483891759
0.0000315063	0.0000576782	0.005878041045	0.000000000438252633	0.000000000517333446	0.008557596439	0.000000000476998714
0.000031673	0.0000580116	0.005909141791	0.00000000044057143	0.00000000052007066	0.008607062315	0.000000000479755932
0.0000318397	0.0000593452	0.005940242537	0.000000000442890227	0.000000000522807874	0.008804925816	0.000000000490784804
0.0000320064	0.0000598453	0.005971343284	0.000000000445209024	0.000000000525545088	0.008879124629	0.000000000494920631
0.0000321731	0.0000608455	0.00600244403	0.000000000447527821	0.000000000528282302	0.009027522255	0.000000000503192285
0.0000323398	0.0000603454	0.006033544776	0.000000000449846618	0.000000000531019516	0.008953323442	0.000000000499056458
0.0000325065	0.0000613456	0.006064645522	0.000000000452165415	0.00000000053375673	0.009101721068	0.000000000507328112
0.0000326732	0.000061679	0.006095746269	0.000000000454484212	0.000000000536493944	0.009151186944	0.00000000051008533
0.0000328399	0.0000605121	0.006126847015	0.000000000456803009	0.000000000539231158	0.00897805638	0.000000000500435067
0.0000330066	0.0000621791	0.006157947761	0.000000000459121806	0.000000000541968372	0.009225385757	0.000000000514221157
0.0000331733	0.0000618457	0.006189048507	0.000000000461440603	0.000000000544705586	0.009175919881	0.000000000511463939
0.00003334	0.0000623458	0.006220149254	0.0000000004637594	0.0000000005474428	0.009250118694	0.000000000515599766
0.0000335067	0.0000626792	0.00625125	0.000000000466078197	0.000000000550180014	0.00929958457	0.000000000518356984
0.0000336734	0.0000625125	0.006282350746	0.000000000468396994	0.000000000552917228	0.009274851632	0.000000000516978375
0.0000338401	0.0000635127	0.006313451493	0.000000000470715791	0.000000000555654442	0.009423249258	0.000000000525250029
0.0000340068	0.0000631793	0.006344552239	0.000000000473034588	0.000000000558391656	0.009373783383	0.000000000522492811
0.0000341735	0.0000638461	0.006375652985	0.000000000475353385	0.00000000056112887	0.009472715134	0.000000000528007247
0.0000343402	0.0000646796	0.006406753731	0.000000000477672182	0.000000000563866084	0.009596379822	0.000000000534900292
0.0000345069	0.0000651797	0.006437854478	0.000000000479990979	0.000000000566603298	0.009670578635	0.000000000539036119
0.0000346736	0.0000643462	0.006468955224	0.000000000482309776	0.000000000569340512	0.009546913947	0.000000000532143074
0.0000348403	0.0000655131	0.00650005597	0.000000000484628573	0.000000000572077726	0.00972004451	0.000000000541793337
0.000035007	0.0000661799	0.006531156716	0.00000000048694737	0.00000000057481494	0.009818976261	0.000000000547307773
0.0000351737	0.0000658465	0.006562257463	0.000000000489266167	0.000000000577552154	0.009769510386	0.000000000544550555

0.0000353404	0.0000663466	0.006593358209	0.000000000491584964	0.000000000580289368	0.009843709199	0.000000000548686382
0.0000355071	0.0000668467	0.006624458955	0.000000000493903761	0.000000000583026582	0.009917908012	0.000000000552822209
0.0000356738	0.0000665133	0.006655559701	0.000000000496222558	0.000000000585763796	0.009868442136	0.000000000550064991
0.0000358405	0.0000673468	0.006686660448	0.000000000498541355	0.00000000058850101	0.009992106825	0.000000000556958036
0.0000360072	0.0000678469	0.006717761194	0.000000000500860152	0.000000000591238224	0.01006630564	0.000000000561093863
0.0000361739	0.0000681803	0.00674886194	0.000000000503178949	0.000000000593975438	0.01011577151	0.000000000563851081
0.0000363406	0.0000676802	0.006779962687	0.000000000505497746	0.000000000596712652	0.0100415727	0.000000000559715254
0.0000365073	0.0000688471	0.006811063433	0.000000000507816543	0.000000000599449866	0.01021470326	0.000000000569365517
0.000036674	0.0000695139	0.006842164179	0.00000000051013534	0.00000000060218708	0.01031363501	0.000000000574879953
0.000000001667	0.0000000030006	0.0000003110074627	0	0	0.0000004451928783	0
0.000000016503	0.0000000278389	0.000003078973881	0	0	0.000004130400593	0
0.000000016336	0.0000000301727	0.000003047873134	0	0	0.000004476661721	0
0.000000016169	0.0000000315063	0.000003016772388	0	0	0.000004674525223	0
0.000000016003	0.0000000296726	0.000002985671642	0	0	0.000004402462908	0
0.000000015836	0.000000028339	0.000002954570896	0	0	0.000004204599407	0
0.000000015669	0.0000000293392	0.000002923470149	0	0	0.000004352997033	0
0.000000015503	0.0000000276722	0.000002892369403	0	0	0.000004105667656	0
0.000000015336	0.0000000298393	0.000002861268657	0	0	0.000004427195846	0
0.000000015169	0.0000000270054	0.00000283016791	0	0	0.000004006735905	0
0.000000015003	0.0000000261719	0.000002799067164	0	0	0.000003883071217	0
0.000000014836	0.0000000255051	0.000002767966418	0	0	0.000003784139466	0
0.000000014669	0.0000000288391	0.000002736865672	0	0	0.00000427879822	0
0.000000014502	0.0000000241715	0.000002705764925	0	0	0.000003586275964	0
0.000000014336	0.0000000225045	0.000002674664179	0	0	0.000003338946588	0
0.000000014169	0.0000000218377	0.000002643563433	0	0	0.000003240014837	0
0.000000014002	0.0000000255051	0.000002612462687	0	0	0.000003784139466	0
0.000000013836	0.000000026672	0.00000258136194	0	0	0.00000395727003	0
0.000000013669	0.0000000238381	0.000002550261194	0	0	0.000003536810089	0
0.000000013502	0.0000000210042	0.000002519160448	0	0	0.000003116350148	0
0.000000013336	0.0000000226712	0.000002488059701	0	0	0.000003363679525	0
0.000000013169	0.0000000240048	0.000002456958955	0	0	0.000003561543027	0
0.000000013002	0.0000000206708	0.000002425858209	0	0	0.000003066884273	0
0.000000012835	0.0000000301727	0.000002394757463	0	0	0.000004476661721	0
0.000000012669	0.0000000185037	0.000002363656716	0	0	0.000002745356083	0
0.000000012502	0.00000001667	0.00000233255597	0	0	0.000002473293769	0
0.000000012335	0.0000000230046	0.000002301455224	0	0	0.000003413145401	0
0.000000012169	0.0000000168367	0.000002270354478	0	0	0.000002498026706	0
0.000000012002	0.0000000203374	0.000002239253731	0	0	0.000003017418398	0
0.000000011835	0.0000000151697	0.000002208152985	0	0	0.000002250697329	0
0.000000011669	0.0000000135027	0.000002177052239	0	0	0.000002003367953	0

0.000000011502	0.0000000145029	0.000002145951493	0	0	0.000002151765579	0
0.000000011335	0.0000000165033	0.000002114850746	0	0	0.000002448560831	0
0.0000000111689	0.0000000128359	0.00000208375	0	0	0.000001904436202	0
0.000000011002	0.0000000123358	0.000002052649254	0	0	0.000001830237389	0
0.000000010835	0.0000000158365	0.000002021548507	0	0	0.00000234962908	0
0.000000010668	0.0000000155031	0.000001990447761	0	0	0.000002300163205	0
0.000000010502	0.0000000148363	0.000001959347015	0	0	0.000002201231454	0
0.000000010335	0.000000020004	0.000001928246269	0	0	0.000002967952522	0
0.000000010168	0.000000026672	0.000001897145522	0	0	0.00000395727003	0
0.000000010002	0.0000000138361	0.000001866044776	0	0	0.000002052833828	0
0.000000009835	0.0000000143362	0.00000183494403	0	0	0.000002127032641	0
0.000000009668	0.000000011669	0.000001803843284	0	0	0.000001731305638	0
0.000000009501	0.0000000161699	0.000001772742537	0	0	0.000002399094955	0
0.000000009335	0.000000015003	0.000001741641791	0	0	0.000002225964392	0
0.000000009168	0.0000000131693	0.000001710541045	0	0	0.000001953902077	0
0.000000009001	0.0000000125025	0.000001679440299	0	0	0.000001854970326	0
0.000000008835	0.0000000121691	0.000001648339552	0	0	0.000001805504451	0
0.000000008668	0.0000000118357	0.000001617238806	0	0	0.000001756038576	0
0.000000008501	0.0000000111689	0.00000158613806	0	0	0.000001657106825	0
0.000000008335	0.0000000101687	0.000001555037313	0	0	0.000001508709199	0
0.000000008168	0.0000000085017	0.000001523936567	0	0	0.000001261379822	0
0.000000008001	0.0000000098353	0.000001492835821	0	0	0.000001459243323	0
0.000000007834	0.0000000105021	0.000001461735075	0	0	0.000001558175074	0
0.000000007668	0.000000008335	0.000001430634328	0	0	0.000001236646884	0
0.000000007501	0.0000000126692	0.000001399533582	0	0	0.000001879703264	0
0.000000007334	0.0000000135027	0.000001368432836	0	0	0.000002003367953	0
0.000000007168	0.0000000103354	0.00000133733209	0	0	0.000001533442136	0
0.000000007001	0.0000000130026	0.000001306231343	0	0	0.000001929169139	0
0.000000006834	0.0000000126692	0.000001275130597	0	0	0.000001879703264	0
0.000000006668	0.0000000130026	0.000001244029851	0	0	0.000001929169139	0
0.000000006501	0.0000000096686	0.000001212929104	0	0	0.000001434510386	0
0.000000006334	0.0000000110022	0.000001181828358	0	0	0.000001632373887	0
0.000000006167	0.0000000106688	0.000001150727612	0	0	0.000001582908012	0
0.000000006001	0.0000000086684	0.000001119626866	0	0	0.00000128611276	0
0.000000005834	0.0000000091685	0.000001088526119	0	0	0.000001360311573	0
0.000000005667	0.0000000080016	0.000001057425373	0	0	0.000001187181009	0
0.000000005501	0.0000000078349	0.000001026324627	0	0	0.000001162448071	0
0.000000005334	0.0000000081683	0.0000009952238806	0	0	0.000001211913947	0
0.000000005167	0.0000000073348	0.0000009641231343	0	0	0.000001088249258	0
0.000000005001	0.0000000088351	0.0000009330223881	0	0	0.000001310845697	0

0.000000004834	0.0000000060012	0.0000009019216418	0	0	0.0000008903857567	0
0.000000004667	0.0000000068347	0.0000008708208955	0	0	0.000001014050445	0
0.000000004500	0.000000005001	0.0000008397201493	0	0	0.0000007419881306	0
0.000000004334	0.0000000065013	0.000000808619403	0	0	0.0000009645845697	0
0.000000004167	0.0000000053344	0.0000007775186567	0	0	0.0000007914540059	0
0.000000004000	0.0000000043342	0.0000007464179104	0	0	0.0000006430563798	0
0.000000003834	0.0000000075015	0.0000007153171642	0	0	0.000001112982196	0
0.000000003667	0.0000000051677	0.0000006842164179	0	0	0.0000007667210682	0
0.000000003500	0.0000000058345	0.0000006531156716	0	0	0.000000865652819	0
0.000000003334	0.0000000038341	0.0000006220149254	0	0	0.0000005688575668	0
0.000000003167	0.0000000055011	0.0000005909141791	0	0	0.0000008161869436	0
0.000000003000	0.0000000036674	0.0000005598134328	0	0	0.0000005441246291	0
0.000000002833	0.0000000031673	0.0000005287126866	0	0	0.000000469925816	0
0.000000002667	0.0000000035007	0.0000004976119403	0	0	0.0000005193916914	0
0.000000002500	0.0000000028339	0.000000466511194	0	0	0.0000004204599407	0
0.000000002333	0.0000000045009	0.0000004354104478	0	0	0.0000006677893175	0
0.000000002167	0.0000000025005	0.0000004043097015	0	0	0.0000003709940653	0
0.000000002000	0.0000000030006	0.0000003732089552	0	0	0.0000004451928783	0
0.000000001833	0.000000003334	0.000000342108209	0	0	0.0000004946587537	0
0.000000000166	0.00000000026672	0.00000003110074627	0	0	0.0000000395727003	0
0.000000001650	0.00000000230046	0.0000003078973881	0	0	0.0000003413145401	0
0.000000001633	0.00000000291725	0.0000003047873134	0	0	0.0000004328264095	0
0.000000001616	0.00000000188371	0.0000003016772388	0	0	0.0000002794821958	0
0.000000001600	0.00000000276722	0.0000002985671642	0	0	0.0000004105667656	0
0.000000001583	0.00000000303394	0.0000002954570896	0	0	0.0000004501394659	0
0.000000001566	0.0000000015003	0.0000002923470149	0	0	0.0000002225964392	0
0.000000001550	0.00000000290058	0.0000002892369403	0	0	0.0000004303531157	0
0.000000001533	0.00000000123358	0.0000002861268657	0	0	0.0000001830237389	0
0.000000001516	0.00000000138361	0.000000283016791	0	0	0.0000002052833828	0
0.000000001500	0.00000000021671	0.0000002799067164	0	0	0.00000003215281899	0
0.000000001483	0.00000000168367	0.0000002767966418	0	0	0.0000002498026706	0
0.000000001466	0.00000000096686	0.0000002736865672	0	0	0.0000001434510386	0
0.000000001450	0.00000000240048	0.0000002705764925	0	0	0.0000003561543027	0
0.000000001433	0.00000000258385	0.0000002674664179	0	0	0.0000003833605341	0
0.000000001416	0.00000000196706	0.0000002643563433	0	0	0.0000002918486647	0
0.000000001400	0.00000000221711	0.0000002612462687	0	0	0.0000003289480712	0
0.000000001383	0.00000000228379	0.000000258136194	0	0	0.0000003388412463	0
0.000000001366	0.00000000205041	0.0000002550261194	0	0	0.0000003042151335	0
0.000000001350	0.00000000251717	0.0000002519160448	0	0	0.0000003734673591	0
0.000000001333	0.00000000198373	0.0000002488059701	0	0	0.0000002943219585	0

0.000000001316	0.00000000238381	0.0000002456958955	0	0	0.0000003536810089		0
0.000000001300	0.00000000215043	0.0000002425858209	0	0	0.0000003190548961		0
0.000000001283	0.00000000195039	0.0000002394757463	0	0	0.0000002893753709		0
0.000000001266	0.00000000248383	0.0000002363656716	0	0	0.0000003685207715		0
0.000000001250	0.00000000181703	0.000000233255597	0	0	0.0000002695890208		0
0.000000001233	0.00000000175035	0.0000002301455224	0	0	0.0000002596958457		0
0.000000001216	0.00000000230046	0.0000002270354478	0	0	0.0000003413145401		0
0.000000001200	0.00000000173368	0.0000002239253731	0	0	0.0000002572225519		0
0.000000001183	0.0000000020004	0.0000002208152985	0	0	0.0000002967952522		0
0.000000001166	0.00000000121691	0.0000002177052239	0	0	0.0000001805504451		0
0.000000001150	0.00000000156698	0.0000002145951493	0	0	0.0000002324896142		0
0.000000001133	0.000000001388611	0.0000002114850746	0	0	0.0000002060253709		0
0.000000001116	0.0000000013336	0.000000208375	0	0	0.0000001978635015		0
0.000000001100	0.000000001156898	0.0000002052649254	0	0	0.0000001716465875		0
0.000000001083	0.000000001111889	0.0000002021548507	0	0	0.0000001649686944		0
0.000000001066	0.000000000926852	0.0000001990447761	0	0	0.0000001375151335		0
0.000000001050	0.000000000740148	0.0000001959347015	0	0	0.0000001098142433		0
0.000000001033	0.000000000838501	0.0000001928246269	0	0	0.0000001244066766		0
0.000000001016	0.000000000670134	0.0000001897145522	0	0	0.0000000994264095		0
0.000000001000	0.000000000556778	0.0000001866044776	0	0	0.00000008260801187		0
0.000000000983	0.000000000490098	0.000000183494403	0	0	0.0000000727148368		0
0.000000000966	0.000000000420084	0.0000001803843284	0	0	0.00000006232700297		0
0.000000000950	0.000000000636794	0.0000001772742537	0	0	0.00000009447982196		0
0.000000000933	0.000000000370074	0.0000001741641791	0	0	0.00000005490712166		0
0.000000000916	0.000000000588451	0.0000001710541045	0	0	0.00000008730727003		0
0.000000000900	0.000000000323398	0.0000001679440299	0	0	0.00000004798189911		0
0.000000000883	0.000000000286724	0.0000001648339552	0	0	0.00000004254065282		0
0.000000000866	0.000000000610122	0.0000001617238806	0	0	0.00000009052255193		0
0.000000000850	0.000000000703474	0.000000158613806	0	0	0.000000104372997		0
0.000000000833	0.000000000686804	0.0000001555037313	0	0	0.0000001018997033		0
0.000000000816	0.00000000030006	0.0000001523936567	0	0	0.00000004451928783		0
0.000000000800	0.00000000023338	0.0000001492835821	0	0	0.00000003462611276		0
0.000000000783	0.00000000018337	0.0000001461735075	0	0	0.00000002720623145		0
0.000000000766	0.000000000273388	0.0000001430634328	0	0	0.0000000405620178		0
0.000000000750	0.000000000218377	0.0000001399533582	0	0	0.00000003240014837		0
0.000000000733	0.000000000168367	0.0000001368432836	0	0	0.00000002498026706		0
0.000000000716	0.000000000203374	0.000000133733209	0	0	0.00000003017418398		0
0.000000000700	0.000000000206708	0.0000001306231343	0	0	0.00000003066884273		0
0.000000000683	0.00000000015003	0.0000001275130597	0	0	0.00000002225964392		0
0.000000000666	0.000000000146696	0.0000001244029851	0	0	0.00000002176498516		0

0.000000000650	0.000000000138361	0.0000001212929104	0	0	0.00000002052833828	0
0.000000000633	0.000000000123358	0.0000001181828358	0	0	0.00000001830237389	0
0.000000000616	0	0.0000001150727612	0	0	0.00000001261379822	0
0.000000000600	0.000000000106688	0.0000001119626866	0	0	0.00000001582908012	0
0.000000000583	0	0.0000001088526119	0	0	0.000000008161869436	0
0.000000000566	0	0.0000001057425373	0	0	0.000000007172551929	0
0.000000000550	0	0.0000001026324627	0	0	0.000000003462611276	0
0.000000000533	0	0.00000009952238806	0	0	0.000000002225964392	0
0.000000000516	0	0.00000009641231343	0	0	0.000000001978635015	0
0.000000000500	0	0.00000009330223881	0	0	0.000000001483976261	0
0.000000000483	0	0.00000009019216418	0	0	0.000000001731305638	0
0.000000000466	0	0.00000008708208955	0	0	0.0000000009893175074	0
0.000000000450	0	0.00000008397201493	0	0	0.0000000004946587537	0
0.000000000433	0	0.0000000808619403	0	0	0.000000001236646884	0
0.000000000416	0	0.00000007775186567	0	0	0.0000000007419881306	0
0.000000000400	0	0.00000007464179104	0	0	0.0000000004946587537	0
0.000000000383	0	0.00000007153171642	0	0	0.0000000002473293769	0
0.000000000366	0.00000000056678	0.00000006842164179	0	0	0.00000008409198813	0
0.000000000350	0.00000000061679	0.00000006531156716	0	0	0.0000000915118694 4	0
0.000000000333	0.00000000048343	0.00000006220149254	0	0	0.00000007172551929	0
0.000000000316	0.00000000030006	0.00000005909141791	0	0	0.00000004451928783	0
0.000000000300	0.00000000053344	0.00000005598134328	0	0	0.00000007914540059	0
0.000000000283	0.00000000035007	0.00000005287126866	0	0	0.00000005193916914	0
0.000000000266	0.00000000031673	0.00000004976119403	0	0	0.0000000469925816	0
0.000000000250	0.00000000020004	0.0000000466511194	0	0	0.00000002967952522	0
0.000000000233	0.00000000018337	0.00000004354104478	0	0	0.00000002720623145	0
0.000000000216	0.00000000025005	0.00000004043097015	0	0	0.00000003709940653	0
0.000000000200	0.00000000013336	0.00000003732089552	0	0	0.00000001978635015	0
0.000000000183	0.00000000035007	0.0000000342108209	0	0	0.00000005193916914	0
0.000000000166	0.00000000028339	0.00000003110074627	0	0	0.00000004204599407	0
0.000000000150	0.00000000026672	0.00000002799067164	0	0	0.0000000395727003	0
0.000000000133	0.00000000023338	0.00000002488059701	0	0	0.00000003462611276	0
0.000000000116	0.0000000001667	0.00000002177052239	0	0	0.00000002473293769	0
0.000000000100	0.00000000011669	0.00000001866044776	0	0	0.00000001731305638	0
0	0	0.00000001555037313	0	0	0.00000001236646884	0
0	0.00000000015003	0.00000001244029851	0	0	0.00000002225964392	0
0	0	0.000000009330223881	0	0	0.000000009893175074	0
0	0	0.000000006220149254	0	0	0.000000004946587537	0
0	0	0.000000003110074627	0	0	0.000000002473293769	0
0.0000368407	0.0000685137	0.006873264925	0.000000000512454137	0.000000000604924294	0.01016523739	0.000000000566608299

0.0000370074	0.0000705141	0.006904365672	0.000000000514772934	0.000000000607661508	0.01046203264	0.000000000583151607
0.0000371741	0.0000691805	0.006935466418	0.000000000517091731	0.000000000610398722	0.01026416914	0.000000000572122735
0.0000373408	0.0000708475	0.006966567164	0.000000000519410528	0.000000000613135936	0.01051149852	0.000000000585908825
0.0000375075	0.0000691805	0.00699766791	0.000000000521729325	0.00000000061587315	0.01026416914	0.000000000572122735
0.0000376742	0.0000710142	0.007028768657	0.000000000524048122	0.000000000618610364	0.01053623145	0.000000000587287434
0.0000378409	0.0000715143	0.007059869403	0.000000000526366919	0.000000000621347578	0.01061043027	0.000000000591423261
0.0000380076	0.0000718477	0.007090970149	0.000000000528685716	0.000000000624084792	0.01065989614	0.000000000594180479
0.0000381743	0.000070014	0.007122070896	0.000000000531004513	0.000000000626822006	0.01038783383	0.00000000057901578
0.000038341	0.0000721811	0.007153171642	0.00000000053332331	0.00000000062955922	0.01070936202	0.000000000596937697
0.0000385077	0.0000703474	0.007184272388	0.000000000535642107	0.000000000632296434	0.0104372997	0.000000000581772998
0.0000386744	0.0000728479	0.007215373134	0.000000000537960904	0.000000000635033648	0.01080829377	0.000000000602452133
0.0000388411	0.0000735147	0.007246473881	0.000000000540279701	0.000000000637770862	0.01090722552	0.000000000607966569
0.0000390078	0.0000741815	0.007277574627	0.000000000542598498	0.000000000640508076	0.01100615727	0.000000000613481005
0.0000391745	0.0000745149	0.007308675373	0.000000000544917295	0.00000000064324529	0.01105562315	0.000000000616238223
0.0000393412	0.0000748483	0.007339776119	0.000000000547236092	0.000000000645982504	0.01110508902	0.000000000618995441
0.0000395079	0.0000731813	0.007370876866	0.000000000549554889	0.000000000648719718	0.01085775964	0.000000000605209351
0.0000396746	0.0000765153	0.007401977612	0.000000000551873686	0.000000000651456932	0.0113524184	0.000000000632781531
0.0000398413	0.0000758485	0.007433078358	0.000000000554192483	0.000000000654194146	0.01125348665	0.000000000627267095
0.000040008	0.000076682	0.007464179104	0.00000000055651128	0.00000000065693136	0.01137715134	0.00000000063416014
0.0000401747	0.0000776822	0.007495279851	0.000000000558830077	0.000000000659668574	0.01152554896	0.000000000642431794
0.0000403414	0.0000781823	0.007526380597	0.000000000561148874	0.000000000662405788	0.01159974777	0.000000000646567621
0.0000405081	0.000078349	0.007557481343	0.000000000563467671	0.000000000665143002	0.01162448071	0.00000000064794623
0.0000406748	0.0000786824	0.00758858209	0.000000000565786468	0.000000000667880216	0.01167394659	0.000000000650703448
0.0000408415	0.0000798493	0.007619682836	0.000000000568105265	0.00000000067061743	0.01184707715	0.000000000660353711
0.0000410082	0.0000790158	0.007650783582	0.000000000570424062	0.000000000673354644	0.01172341246	0.000000000653460666
0.0000411749	0.0000795159	0.007681884328	0.000000000572742859	0.000000000676091858	0.01179761128	0.000000000657596493
0.0000413416	0.0000801827	0.007712985075	0.000000000575061656	0.000000000678829072	0.01189654303	0.000000000663110929
0.0000415083	0.0000815163	0.007744085821	0.000000000577380453	0.000000000681566286	0.01209440653	0.000000000674139801
0.000041675	0.0000811829	0.007775186567	0.00000000057969925	0.0000000006843035	0.01204494065	0.000000000671382583
0.0000418417	0.000081683	0.007806287313	0.000000000582018047	0.000000000687040714	0.01211913947	0.00000000067551841
0.0000420084	0.0000820164	0.00783738806	0.000000000584336844	0.000000000689777928	0.01216860534	0.000000000678275628
0.0000421751	0.0000821831	0.007868488806	0.000000000586655641	0.000000000692515142	0.01219333828	0.000000000679654237
0.0000423418	0.0000825165	0.007899589552	0.000000000588974438	0.000000000695252356	0.01224280415	0.000000000682411455
0.0000425085	0.0000806828	0.007930690299	0.000000000591293235	0.00000000069798957	0.01197074184	0.000000000667246756
0.0000426752	0.0000826832	0.007961791045	0.000000000593612032	0.000000000700726784	0.01226753709	0.000000000683790064
0.0000428419	0.0000830166	0.007992891791	0.000000000595930829	0.000000000703463998	0.01231700297	0.000000000686547282
0.0000430086	0.0000835167	0.008023992537	0.000000000598249626	0.000000000706201212	0.01239120178	0.000000000690683109
0.0000431753	0.0000841835	0.008055093284	0.000000000600568423	0.000000000708938426	0.01249013353	0.000000000696197545
0.000043342	0.0000845169	0.00808619403	0.00000000060288722	0.00000000071167564	0.01253959941	0.000000000698954763
0.0000435087	0.0000838501	0.008117294776	0.000000000605206017	0.000000000714412854	0.01244066766	0.000000000693440327

0.0000436754	0.0000846836	0.008148395522	0.000000000607524814	0.000000000717150068	0.01256433234	0.000000000700333372
0.0000438421	0.0000851837	0.008179496269	0.000000000609843611	0.000000000719887282	0.01263853116	0.000000000704469199
0.0000440088	0.0000855171	0.008210597015	0.000000000612162408	0.000000000722624496	0.01268799703	0.000000000707226417
0.0000441755	0.0000858505	0.008241697761	0.000000000614481205	0.00000000072536171	0.01273746291	0.000000000709983635
0.0000443422	0.0000860172	0.008272798507	0.000000000616800002	0.000000000728098924	0.01276219585	0.000000000711362244
0.0000445089	0.0000865173	0.008303899254	0.000000000619118799	0.000000000730836138	0.01283639466	0.000000000715498071
0.0000446756	0.0000868507	0.008335	0.000000000621437596	0.000000000733573352	0.01288586053	0.000000000718255289
0.0000448423	0.0000873508	0.008366100746	0.000000000623756393	0.000000000736310566	0.01296005935	0.000000000722391116
0.000045009	0.0000875175	0.008397201493	0.00000000062607519	0.00000000073904778	0.01298479228	0.000000000723769725
0.0000451757	0.0000878509	0.008428302239	0.000000000628393987	0.000000000741784994	0.01303425816	0.000000000726526943
0.0000453424	0.0000848503	0.008459402985	0.000000000630712784	0.000000000744522208	0.01258906528	0.000000000701711981
0.0000455091	0.0000888511	0.008490503731	0.000000000633031581	0.000000000747259422	0.01318265579	0.000000000734798597
0.0000456758	0.0000895179	0.008521604478	0.000000000635350378	0.000000000749996636	0.01328158754	0.000000000740313033
0.0000458425	0.000090018	0.008552705224	0.000000000637669175	0.00000000075273385	0.01335578635	0.00000000074444886
0.0000460092	0.0000903514	0.00858380597	0.000000000639987972	0.000000000755471064	0.01340525223	0.000000000747206078
0.0000461759	0.0000915183	0.008614906716	0.000000000642306769	0.000000000758208278	0.01357838279	0.000000000756856341
0.0000463426	0.0000925185	0.008646007463	0.000000000644625566	0.000000000760945492	0.01372678042	0.000000000765127995
0.0000465093	0.0000910182	0.008677108209	0.000000000646944363	0.000000000763682706	0.01350418398	0.000000000752720514
0.000046676	0.0000921851	0.008708208955	0.00000000064926316	0.00000000076641992	0.01367731454	0.000000000762370777
0.0000468427	0.0000891845	0.008739309701	0.000000000651581957	0.000000000769157134	0.01323212166	0.000000000737555815
0.0000470094	0.0000881843	0.008770410448	0.000000000653900754	0.000000000771894348	0.01308372404	0.000000000729284161
0.0000471761	0.0000906848	0.008801511194	0.000000000656219551	0.000000000774631562	0.0134547181	0.000000000749963296
0.0000473428	0.0000885177	0.00883261194	0.000000000658538348	0.000000000777368776	0.01313318991	0.000000000732041379
0.0000475095	0.0000935187	0.008863712687	0.000000000660857145	0.00000000078010599	0.01387517804	0.000000000773399649
0.0000476762	0.0000941855	0.008894813433	0.000000000663175942	0.000000000782843204	0.01397410979	0.000000000778914085
0.0000478429	0.0000945189	0.008925914179	0.000000000665494739	0.000000000785580418	0.01402357567	0.000000000781671303
0.0000480096	0.0000946856	0.008957014925	0.000000000667813536	0.000000000788317632	0.01404830861	0.000000000783049912
0.0000481763	0.0000948523	0.008988115672	0.000000000670132333	0.000000000791054846	0.01407304154	0.000000000784428521
0.000048343	0.0000955191	0.009019216418	0.00000000067245113	0.00000000079379206	0.01417197329	0.000000000789942957
0.0000485097	0.0000936854	0.009050317164	0.000000000674769927	0.000000000796529274	0.01389991098	0.000000000774778258
0.0000486764	0.0000956858	0.00908141791	0.000000000677088724	0.000000000799266488	0.01419670623	0.000000000791321566
0.0000488431	0.0000960192	0.009112518657	0.000000000679407521	0.000000000802003702	0.01424617211	0.000000000794078784
0.0000490098	0.0000961859	0.009143619403	0.000000000681726318	0.000000000804740916	0.01427090504	0.000000000795457393
0.0000491765	0.0000963526	0.009174720149	0.000000000684045115	0.00000000080747813	0.01429563798	0.000000000796836002
0.0000493432	0.0000973528	0.009205820896	0.000000000686363912	0.000000000810215344	0.01444403561	0.000000000805107656
0.0000495099	0.0000968527	0.009236921642	0.000000000688682709	0.000000000812952558	0.0143698368	0.000000000800971829
0.0000496766	0.0000951857	0.009268022388	0.000000000691001506	0.000000000815689772	0.01412250742	0.000000000787185739
0.0000498433	0.0000981863	0.009299123134	0.000000000693320303	0.000000000818426986	0.0145677003	0.000000000812000701
0.00005001	0.0000978529	0.009330223881	0.0000000006956391	0.0000000008211642	0.01451823442	0.000000000809243483
0.0000501767	0.0000993532	0.009361324627	0.000000000697957897	0.000000000823901414	0.01474083086	0.000000000821650964

0.0000503434	0.0000931853	0.009392425373	0.000000000700276694	0.000000000826638628	0.01382571217	0.000000000770642431
0.0000505101	0.0000995199	0.009423526119	0.000000000702595491	0.000000000829375842	0.0147655638	0.000000000823029573
0.0000506768	0.0000988531	0.009454626866	0.000000000704914288	0.000000000832113056	0.01466663205	0.000000000817515137
0.0000508435	0.0000985197	0.009485727612	0.000000000707233085	0.00000000083485027	0.01461716617	0.000000000814757919
0.0000510102	0.0000998533	0.009516828358	0.000000000709551882	0.000000000837587484	0.01481502967	0.000000000825786791
0.0000511769	0.0000991865	0.009547929104	0.000000000711870679	0.000000000840324698	0.01471609792	0.000000000820272355
0.0000513436	0.0001005201	0.009579029851	0.000000000714189476	0.000000000843061912	0.01491396142	0.000000000831301227
0.0000515103	0.0001008535	0.009610130597	0.000000000716508273	0.000000000845799126	0.0149634273	0.000000000834058445
0.000051677	0.0001013536	0.009641231343	0.00000000071882707	0.00000000084853634	0.01503762611	0.000000000838194272
0.0000518437	0.000101687	0.00967233209	0.000000000721145867	0.000000000851273554	0.01508709199	0.00000000084095149
0.0000520104	0.0001001867	0.009703432836	0.000000000723464664	0.000000000854010768	0.01486449555	0.000000000828544009
0.0000521771	0.0001018537	0.009734533582	0.000000000725783461	0.000000000856747982	0.01511182493	0.000000000842330099
0.0000523438	0.0001021871	0.009765634328	0.000000000728102258	0.000000000859485196	0.0151612908	0.000000000845087317
0.0000525105	0.0001023538	0.009796735075	0.000000000730421055	0.00000000086222241	0.01518602374	0.000000000846465926
0.0000526772	0.0001011869	0.009827835821	0.000000000732739852	0.000000000864959624	0.01501289318	0.000000000836815663
0.0000528439	0.0001030206	0.009858936567	0.000000000735058649	0.000000000867696838	0.01528495549	0.000000000851980362
0.0000530106	0.0001035207	0.009890037313	0.000000000737377446	0.000000000870434052	0.0153591543	0.000000000856116189
0.0000531773	0.0001038541	0.00992113806	0.000000000739696243	0.000000000873171266	0.01540862018	0.000000000858873407
0.000053344	0.0001046876	0.009952238806	0.00000000074201504	0.00000000087590848	0.01553228487	0.000000000865766452
0.0000535107	0.0001026872	0.009983339552	0.000000000744333837	0.000000000878645694	0.01523548961	0.000000000849223144
0.0000536774	0.0001041875	0.0100144403	0.000000000746652634	0.000000000881382908	0.01545808605	0.000000000861630625
0.0000538441	0.000105021	0.01004554104	0.000000000748971431	0.000000000884120122	0.01558175074	0.00000000086852367
0.0000540108	0.0001053544	0.01007664179	0.000000000751290228	0.000000000886857336	0.01563121662	0.000000000871280888
0.0000541775	0.0001055211	0.01010774254	0.000000000753609025	0.00000000088959455	0.01565594955	0.000000000872659497
0.0000543442	0.0001065213	0.01013884328	0.000000000755927822	0.000000000892331764	0.01580434718	0.000000000880931151
0.0000545109	0.0001061879	0.01016994403	0.000000000758246619	0.000000000895068978	0.01575488131	0.000000000878173933
0.0000546776	0.0001070214	0.01020104478	0.000000000760565416	0.000000000897806192	0.01587854599	0.000000000885066978
0.0000548443	0.0001073548	0.01023214552	0.000000000762884213	0.000000000900543406	0.01592801187	0.000000000887824196
0.000055011	0.0001068547	0.01026324627	0.00000000076520301	0.00000000090328062	0.01585381306	0.000000000883688369
0.0000551777	0.0001058545	0.01029434701	0.000000000767521807	0.000000000906017834	0.01570541543	0.000000000875416715
0.0000553444	0.0001085217	0.01032544776	0.000000000769840604	0.000000000908755048	0.01610114243	0.000000000897474459
0.0000555111	0.0001076882	0.01035654851	0.000000000772159401	0.000000000911492262	0.01597747774	0.000000000890581414
0.0000556778	0.0001081883	0.01038764925	0.000000000774478198	0.000000000914229476	0.01605167656	0.000000000894717241
0.0000558445	0.0001090218	0.01041875	0.000000000776796995	0.00000000091696669	0.01617534125	0.000000000901610286
0.0000560112	0.0001093552	0.01044985075	0.000000000779115792	0.000000000919703904	0.01622480712	0.000000000904367504
0.0000561779	0.0001101887	0.01048095149	0.000000000781434589	0.000000000922441118	0.01634847181	0.000000000911260549
0.0000563446	0.0001106888	0.01051205224	0.000000000783753386	0.000000000925178332	0.01642267062	0.000000000915396376
0.0000565113	0.0001108555	0.01054315299	0.000000000786072183	0.000000000927915546	0.01644740356	0.000000000916774985
0.000056678	0.0001096886	0.01057425373	0.00000000078839098	0.00000000093065276	0.016274273	0.000000000907124722
0.0000568447	0.000111689	0.01060535448	0.000000000790709777	0.000000000933389974	0.01657106825	0.00000000092366803

0.0000570114	0.0001113556	0.01063645522	0.000000000793028574	0.000000000936127188	0.01652160237	0.000000000920910812
0.0000571781	0.0001125225	0.01066755597	0.000000000795347371	0.000000000938864402	0.01669473294	0.000000000930561075
0.0000573448	0.0001121891	0.01069865672	0.000000000797666168	0.000000000941601616	0.01664526706	0.000000000927803857
0.0000575115	0.0001128559	0.01072975746	0.000000000799984965	0.00000000094433883	0.01674419881	0.000000000933318293
0.0000576782	0.0001135227	0.01076085821	0.000000000802303762	0.000000000947076044	0.01684313056	0.000000000938832729
0.0000578449	0.0001143562	0.01079195896	0.000000000804622559	0.000000000949813258	0.01696679525	0.000000000945725774
0.0000580116	0.0001138561	0.0108230597	0.000000000806941356	0.000000000952550472	0.01689259644	0.000000000941589947
0.0000581783	0.0001140228	0.01085416045	0.000000000809260153	0.000000000955287686	0.01691732938	0.000000000942968556
0.000058345	0.0001098553	0.01088526119	0.00000000081157895	0.0000000009580249	0.01629900593	0.000000000908503331
0.0000585117	0.0001151897	0.01091636194	0.000000000813897747	0.000000000960762114	0.01709045994	0.000000000952618819
0.0000586784	0.0001161899	0.01094746269	0.000000000816216544	0.000000000963499328	0.01723885757	0.000000000960890473
0.0000588451	0.0001156898	0.01097856343	0.000000000818535341	0.000000000966236542	0.01716465875	0.000000000956754646
0.0000590118	0.0001148563	0.01100966418	0.000000000820854138	0.000000000968973756	0.01704099407	0.000000000949861601
0.0000591785	0.0001165233	0.01104076493	0.000000000823172935	0.00000000097171097	0.01728832344	0.000000000963647691
0.0000593452	0.0001155231	0.01107186567	0.000000000825491732	0.000000000974448184	0.01713992582	0.000000000955376037
0.0000595119	0.0001136894	0.01110296642	0.000000000827810529	0.000000000977185398	0.0168678635	0.000000000940211338
0.0000596786	0.0001118557	0.01113406716	0.000000000830129326	0.000000000979922612	0.01659580119	0.000000000925046639
0.0000598453	0.0001143562	0.01116516791	0.000000000832448123	0.000000000982659826	0.01696679525	0.000000000945725774
0.000060012	0.0001163566	0.01119626866	0.00000000083476692	0.00000000098539704	0.0172635905	0.000000000962269082
0.0000601787	0.0001168567	0.0112273694	0.000000000837085717	0.000000000988134254	0.01733778932	0.000000000966404909
0.0000603454	0.0001180236	0.01125847015	0.000000000839404514	0.000000000990871468	0.01751091988	0.000000000976055172
0.0000605121	0.0001171901	0.0112895709	0.000000000841723311	0.000000000993608682	0.01738725519	0.000000000969162127
0.0000606788	0.0001185237	0.01132067164	0.000000000844042108	0.000000000996345896	0.01758511869	0.000000000980190999
0.0000608455	0.0001193572	0.01135177239	0.000000000846360905	0.00000000099908311	0.01770878338	0.000000000987084044
0.0000610122	0.0001188571	0.01138287313	0.000000000848679702	0.000000001001820324	0.01763458457	0.000000000982948217
0.0000611789	0.0001191905	0.01141397388	0.000000000850998499	0.000000001004557538	0.01768405045	0.000000000985705435
0.0000613456	0.0001198573	0.01144507463	0.000000000853317296	0.000000001007294752	0.0177829822	0.000000000991219871
0.0000615123	0.0001203574	0.01147617537	0.000000000855636093	0.000000001010031966	0.01785718101	0.000000000995355698
0.000061679	0.0001210242	0.01150727612	0.00000000085795489	0.00000000101276918	0.01795611276	0.000000001000870134
0.0000618457	0.0001213576	0.01153837687	0.000000000860273687	0.000000001015506394	0.01800557864	0.000000001003627352
0.0000620124	0.0001218577	0.01156947761	0.000000000862592484	0.000000001018243608	0.01807977745	0.000000001007763179
0.0000621791	0.0001220244	0.01160057836	0.000000000864911281	0.000000001020980822	0.01810451039	0.000000001009141788
0.0000623458	0.0001206908	0.0116316791	0.000000000867230078	0.000000001023718036	0.01790664688	0.000000000998112916
0.0000625125	0.0001226912	0.01166277985	0.000000000869548875	0.00000000102645525	0.01820344214	0.000000001014656224
0.0000626792	0.0001231913	0.0116938806	0.000000000871867672	0.000000001029192464	0.01827764095	0.000000001018792051
0.0000628459	0.0001235247	0.01172498134	0.000000000874186469	0.000000001031929678	0.01832710682	0.000000001021549269
0.0000630126	0.0001240248	0.01175608209	0.000000000876505266	0.000000001034666892	0.01840130564	0.000000001025685096
0.0000631793	0.0001245249	0.01178718284	0.000000000878824063	0.000000001037404106	0.01847550445	0.000000001029820923
0.000063346	0.000125025	0.01181828358	0.00000000088114286	0.00000000104014132	0.01854970326	0.00000000103395675
0.0000635127	0.0001248583	0.01184938433	0.000000000883461657	0.000000001042878534	0.01852497033	0.000000001032578141

0.0000636794	0.0001255251	0.01188048507	0.000000000885780454	0.000000001045615748	0.01862390208	0.000000001038092577
0.0000638461	0.0001256918	0.01191158582	0.000000000888099251	0.000000001048352962	0.01864863501	0.000000001039471186
0.0000640128	0.0001236914	0.01194268657	0.000000000890418048	0.000000001051090176	0.01835183976	0.000000001022927878
0.0000641795	0.0001263586	0.01197378731	0.000000000892736845	0.00000000105382739	0.01874756677	0.000000001044985622
0.0000643462	0.0001268587	0.01200488806	0.000000000895055642	0.000000001056564604	0.01882176558	0.000000001049121449
0.0000645129	0.0001238581	0.01203598881	0.000000000897374439	0.000000001059301818	0.0183765727	0.000000001024306487
0.0000646796	0.0001265253	0.01206708955	0.000000000899693236	0.000000001062039032	0.0187722997	0.000000001046364231
0.0000648463	0.0001230246	0.0120981903	0.000000000902012033	0.000000001064776246	0.01825290801	0.000000001017413442
0.000065013	0.0001275255	0.01212929104	0.00000000090433083	0.00000000106751346	0.01892069733	0.000000001054635885
0.0000651797	0.0001278589	0.01216039179	0.000000000906649627	0.000000001070250674	0.0189701632	0.000000001057393103
0.0000653464	0.0001293592	0.01219149254	0.000000000908968424	0.000000001072987888	0.01919275964	0.000000001069800584
0.0000655131	0.0001285257	0.01222259328	0.000000000911287221	0.000000001075725102	0.01906909496	0.000000001062907539
0.0000656798	0.0001298593	0.01225369403	0.000000000913606018	0.000000001078462316	0.01926695846	0.000000001073936411
0.0000658465	0.0001258585	0.01228479478	0.000000000915924815	0.00000000108119953	0.01867336795	0.000000001040849795
0.0000660132	0.0001303594	0.01231589552	0.000000000918243612	0.000000001083936744	0.01934115727	0.000000001078072238
0.0000661799	0.0001313596	0.01234699627	0.000000000920562409	0.000000001086673958	0.0194895549	0.000000001086343892
0.0000663466	0.0001308595	0.01237809701	0.000000000922881206	0.000000001089411172	0.01941535608	0.000000001082208065
0.0000665133	0.0001315263	0.01240919776	0.000000000925200003	0.000000001092148386	0.01951428783	0.000000001087722501
0.00006668	0.000131693	0.01244029851	0.0000000009275188	0.0000000010948856	0.01953902077	0.00000000108910111
0.0000668467	0.0001320264	0.01247139925	0.000000000929837597	0.000000001097622814	0.01958848665	0.000000001091858328
0.0000670134	0.0001326932	0.0125025	0.000000000932156394	0.000000001100360028	0.0196874184	0.000000001097372764
0.0000671801	0.0001281923	0.01253360075	0.000000000934475191	0.000000001103097242	0.01901962908	0.000000001060150321
0.0000673468	0.0001306928	0.01256470149	0.000000000936793988	0.000000001105834456	0.01939062315	0.000000001080829456
0.0000675135	0.0001331933	0.01259580224	0.000000000939112785	0.00000000110857167	0.01976161721	0.000000001101508591
0.0000676802	0.0001323598	0.01262690299	0.000000000941431582	0.000000001111308884	0.01963795252	0.000000001094615546
0.0000678469	0.00013336	0.01265800373	0.000000000943750379	0.000000001114046098	0.01978635015	0.0000000011028872
0.0000680136	0.0001338601	0.01268910448	0.000000000946069176	0.000000001116783312	0.01986054896	0.000000001107023027
0.0000681803	0.0001341935	0.01272020522	0.000000000948387973	0.000000001119520526	0.01991001484	0.000000001109780245
0.000068347	0.0001343602	0.01275130597	0.00000000095070677	0.00000000112225774	0.01993474777	0.000000001111158854
0.0000685137	0.0001348603	0.01278240672	0.000000000953025567	0.000000001124994954	0.02000894659	0.000000001115294681
0.0000686804	0.0001351937	0.01281350746	0.000000000955344364	0.000000001127732168	0.02005841246	0.000000001118051899
0.0000688471	0.0001358605	0.01284460821	0.000000000957663161	0.000000001130469382	0.02015734421	0.000000001123566335
0.0000690138	0.0001355271	0.01287570896	0.000000000959981958	0.000000001133206596	0.02010787834	0.000000001120809117
0.0000691805	0.0001365273	0.0129068097	0.000000000962300755	0.00000000113594381	0.02025627596	0.000000001129080771
0.0000693472	0.0001356938	0.01293791045	0.000000000964619552	0.000000001138681024	0.02013261128	0.000000001122187726
0.0000695139	0.0001371941	0.01296901119	0.000000000966938349	0.000000001141418238	0.02035520772	0.000000001134595207
0.0000696806	0.0001380276	0.01300011194	0.000000000969257146	0.000000001144155452	0.0204788724	0.000000001141488252
0.0000698473	0.000138361	0.01303121269	0.000000000971575943	0.000000001146892666	0.02052833828	0.00000000114424547
0.000070014	0.0001376942	0.01306231343	0.00000000097389474	0.00000000114962988	0.02042940653	0.000000001138731034
